# Country specific hybridization of honey bees from lineage M

**DOI:** 10.1093/gigascience/giag093

**Published:** 2026-09-15

**Authors:** Julita Machlowska, Dariusz Gerula, Andrzej Oleksa, Henk Kok, Grace P McCormack, Alexandra Valentine, Lars Kirkerud, Per Kryger, Laima Blažytė-Čereškienė, Martin Hasselmann, Teweldemedhn Gebretinsae Hailu, Benjamin Rutschmann, Adam Tofilski

**Affiliations:** Department of Zoology and Animal Welfare, University of Agriculture in Krakow, 29 Listopada 56, 31-425 Kraków, Poland; Apiculture Division, National Institute of Horticultural Research, 24-100 Pulawy, Poland; Department of Genetics, Kazimierz Wielki University, Powstańców Wielkopolskich 10, 85-090 Bydgoszcz, Poland; Nederlandse Bijenhoudersvereniging, Stationsweg 94A, 6711 PW, Ede, Netherlands; Molecular Evolution and Systematics Laboratory, University of Galway, University Rd., H91 TK33 Galway, Ireland; Molecular Evolution and Systematics Laboratory, University of Galway, University Rd., H91 TK33 Galway, Ireland; Norsk Brunbielag, Dampsagveien 14, 2004 Lillestrøm, Norway; Department of Agroecology—Agricultural biodiversity, Aarhus University, Forsøgsvej 1, 4200 Slagelse, Denmark; Nature Research Centre, Akademijos St. 2, 08412 Vilnius, Lithuania; Department of Population Genomics, Institute of Animal Science, University of Hohenheim, Garbenstr. 17, 70599 Stuttgart, Germany; Department of Animal Breeding and Husbandry in the Tropics and Subtropics, University of Hohenheim, Garbenstr. 17, 70599 Stuttgart, Germany; Agroecology and Environment, Agroscope, Reckenholzstrasse 191, 8046 Zurich, Switzerland; Department of Animal Ecology and Tropical Biology, Biocenter, University of Würzburg, Am Hubland, 97074 Würzburg, Germany; Department of Zoology and Animal Welfare, University of Agriculture in Krakow, 29 Listopada 56, 31-425 Kraków, Poland

**Keywords:** *Apis mellifera*, wing, geometric morphometrics, M-lineage bees, northwestern Europe

## Abstract

**Background:**

Honey bees are essential pollinators supporting agricultural production and wild plant diversity. In evolutionary lineage M, some populations are threatened by genetic erosion caused by the widespread introduction of commercially bred queens. To assess this risk, wing images from existing and new datasets were used to assign them to 4 evolutionary lineages (A, C, M, and O). The new dataset consisted of 29,043 wing images representing 1,342 colony samples from 10 countries.

**Results:**

Overall, 63.7% of colonies belonged to lineage M, whereas 27.5% were classified as A, 7.9% as C, and 0.8% as O. Lineage M remains prevalent in unprotected populations in Portugal, Spain, and Ireland, as well as in protected populations elsewhere. In contrast, a pronounced decline was observed in unprotected populations in northeastern Poland.

**Conclusions:**

These findings reveal strong regional differences in the persistence of lineage M and underscore the need for coordinated conservation efforts throughout Europe. The data provided in this study should allow for more accurate discrimination between native and introduced phenotypes.

## Introduction

Honey bees (*Apis mellifera*, NCBI:txid7460) are valuable pollinators that contribute significantly to agricultural productivity and the maintenance of wild plant diversity. In agriculture, their pollination services are essential for many fruit, vegetable, and nut crops. The global annual economic value directly attributable to animal pollination is estimated to be in the hundreds of billions of US dollars [1]. Beyond agriculture, honey bees contribute to the pollination of a vast array of wild plants, supporting ecosystem function and the reproduction of non-crop flora, which is essential for broader biodiversity [2]. The resilience of these pollination services is crucially linked to genetic diversity within honey bee populations. Subspecies and ecotypes adapted to specific climatic and floral conditions often have superior survival rates in their native ranges compared to non-adapted, commercially imported stocks [3, 4]. Managed honey bee populations in the USA and parts of Europe have experienced significant declines, often categorized as colony collapse disorder [5, 6], and evidence also indicates declines in wild-living honey bee populations across much of Europe [7]. Apart from primary drivers such as pathogens and agrochemicals, the loss of population genetic variability and the erosion of adaptation to the local environment have been suggested as causes of the decline [8–10]. Therefore, preserving the intraspecific biodiversity of honey bees is a conservation imperative and a practical necessity for sustaining robust pollination services and agricultural security in the face of environmental change [11, 12].

The natural distribution of *A. mellifera* includes Africa, Europe, and Western Asia; however, this species is found worldwide due to multiple introductions [13, 14]. Approximately 30 honey bee subspecies have been identified [15–18]. Four major evolutionary lineages are recognized within the species: the African lineage (A), the Central and Eastern European lineage (C), the Western and Northern European lineage (M), and the Middle Eastern and Central Asian lineage (O) [16, 19]. Recently, honey bees from Ethiopia, originally classified as lineage A, were reclassified as lineage Y using molecular and wing morphometric analyses [20]. Another lineage, lineage Z, was identified in Syria and Lebanon [21, 22].

Lineage M’s natural distribution extends from Ireland and Portugal in the west to the Ural Mountains in Russia in the east [16]. Recently, it was suggested that lineage M also occurs naturally in western China [23]. The northernmost limit of the natural distribution of *A. mellifera* and its M lineage is southern Scandinavia, around 60°N [24]. The southernmost limits of lineage M extend along the Mediterranean coast of Spain and France (including Corsica) and the northern slopes of the Alps. Further east, there was a hybrid zone between lineages M and C, which was less clearly defined. It was assumed that lineage M occurred in northern Austria, the Czech Republic, most of Poland, and the northern part of Ukraine [16, 25]. Within this distribution, 3 subspecies were distinguished: *A. mellifera iberiensis* in the Iberian Peninsula; *A. mellifera mellifera* in the rest of the European distribution of lineage M; and *A. mellifera sinisxinyuan* in western China [23, 26, 27]. Geographic variation within the subspecies is represented by distinct ecotypes [4, 11, 25, 27, 28].

In some parts of Europe, native honey bee populations were significantly influenced by human management, including the importation of queens, transhumance movements, and breeding programs [9, 11, 29, 30]. Since the early 20th century, commercial beekeeping has favored non-native subspecies from lineage C, particularly *A. mellifera carnica* and *A. mellifera ligustica*, as well as their hybrids. Beekeepers widely perceived these subspecies as more suitable for apiculture than the native *A. m. mellifera* or found that hybrids between their original M-lineage bees and introduced bees were more productive due to short-term heterosis effects [31, 32]. The extensive gene flow between native populations and commercially propagated bees has led to the disappearance or significant genetic erosion of indigenous honey bees in many European regions [33]. In Germany, for instance, the large-scale introduction of non-native queens led to the widespread replacement of *A. m. mellifera* with *A. m. carnica* [34, 35]. Similar patterns of replacement or strong introgression have been documented in the Czech Republic [36, 37], Denmark [38], and parts of Poland [39, 40]. Currently, in many regions of Europe, most professional and amateur beekeepers maintain *A. m. ligustica, A. m. carnica*, or their hybrids. Consequently, the natural distribution range of lineage M, particularly *A. m. mellifera*, has substantially decreased in recent decades [10, 41], and it continues to be threatened by further introgression.

The degree of genetic introgression varies considerably across northwestern Europe. While many regions exhibit high levels of admixture with C-lineage bees, several areas with relatively low levels of introgression have been identified. These regions include the Iberian Peninsula [42–46], Ireland [47–50], the Inner Hebrides [51, 52], northeastern Poland [53], and some protected areas in the Netherlands, Norway, France, Denmark, and Switzerland [25, 51, 52, 54, 55]. These regions are therefore considered important genetic refuges for lineage M.

Conserving honey bees is crucial for maintaining their genetic diversity and local adaptations to different environments. Global concern over the loss of honey bee genetic resources has grown in recent years [41], prompting the development of multiple conservation initiatives across Europe [11, 56]. One of the most prominent efforts is the International Association for the Conservation of the European Dark Honey Bee (SICAMM), which was established in 1994. SICAMM unites beekeepers, researchers, and conservationists in support of preserving and promoting *A. m. mellifera* across its historical range [57]. Effective conservation management requires reliable, cost-effective, and practical tools to detect introgression between honey bee subspecies. These methods are essential for monitoring genetic integrity, guiding breeding programs, and preventing further introgression in conservation areas. Contemporary methodologies for identifying honey bee subspecies are based on 2 contrasting approaches: molecular markers and morphometry [27]. Molecular markers, such as microsatellites [58] and, more recently, single nucleotide polymorphisms [28, 51, 59], constitute a powerful identification toolkit. However, beekeepers’ use of these methods is severely limited due to the need for specialized personnel, costly equipment and reagents, and the lack of rapid accessibility, which makes routine colony analysis unaffordable. In contrast, morphometric identification based on wing venation [16, 27, 60] is a highly cost-effective alternative that can be more easily implemented in apiaries. This approach requires only a microscope with an attached camera to deliver a practical, affordable tool for beekeepers. Morphometric identification of honey bee subspecies is relatively easy using freely available software [57, 61]; however, it requires reference datasets for comparison. Unfortunately, access to publicly available reference data is limited. Paradoxically, there is much more reference data for *A. m. iberiensis* [62]—a well-protected subspecies—than for *A. m. mellifera* [61], an endangered subspecies.

We provide a large amount of data on wing variation in northwestern Europe that can be used to monitor local populations in the future. Furthermore, we demonstrate that the hybridization process of *A. m. mellifera* has accelerated recently, and urgent action is required to prevent the complete extinction of this subspecies.

## Methods

### Material

**Figure 1:**
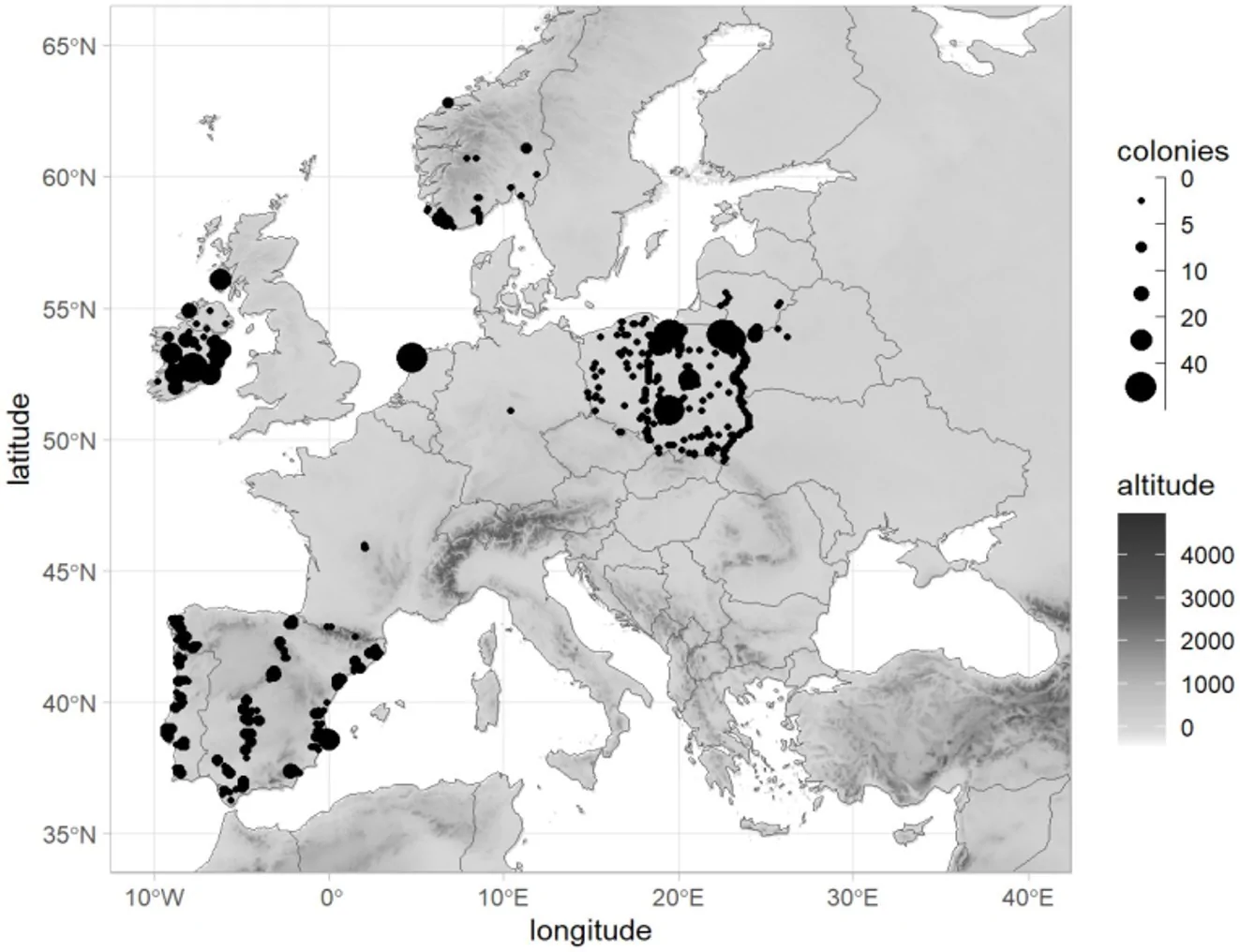
Locations from which honey bee samples were collected. Map created using the rnaturalearth R package.

In this study, we provide 29,043 images of honey bee worker wings in a public repository, which is a new contribution. These images represent 1,342 samples from 10 countries. Some of these samples were related to previous studies: samples 1–4 from Germany [63]; samples 517–537 from Spain [46]; samples 1–304 from Ireland [50]; samples 640–679 and 680–922 from Poland [64, 65]. Material from Belarus, France, the UK, Lithuania, the Netherlands, and Norway, and most material from Poland was not presented in earlier publications. Samples from Belarus were collected in 2016 from log hives in an unprotected population. Samples from France were collected from both log hives and managed hives, which originated from an unprotected population. Samples from the UK were collected from the protected population on Colonsay Island in 2014. Samples from Lithuania were collected from small, traditionally managed apiaries situated in forested regions and remote from apiaries practicing modern beekeeping. Samples from the Netherlands were collected in 2024 from the protected population on Texel Island. Samples from Norway were collected from the Flekkefjord conservation area (29 colonies) and other unprotected parts of the country (55 colonies). Most samples from Poland originated from protected populations, which are maintained by honey bee breeders using instrumental insemination. The number of workers per sample in the new dataset ranged from 5 to 69, with an average of 21.6. We also remeasured 449 wings representing 9 colonies from Cuba [66, 67] and used data from earlier studies related to Poland, Portugal, and Spain [62, 68]. In total, we used 38,990 wing images, representing 2,312 samples and 12 countries, in the analysis (Table 1, Fig. 1).

**Table 1:** Sample size of honey bee wings used in this study

Country	Number of wings	Number of samples
Belarus	171	5
Cuba	449	9
Germany	60	4
Spain	3,482	573
France	164	11
UK	425	29
Ireland	3,498	304
Lithuania	1,358	76
Netherlands	3,555	125
Norway	2,274	84
Poland	22,574	900
Portugal	960	192

**Figure 2:**
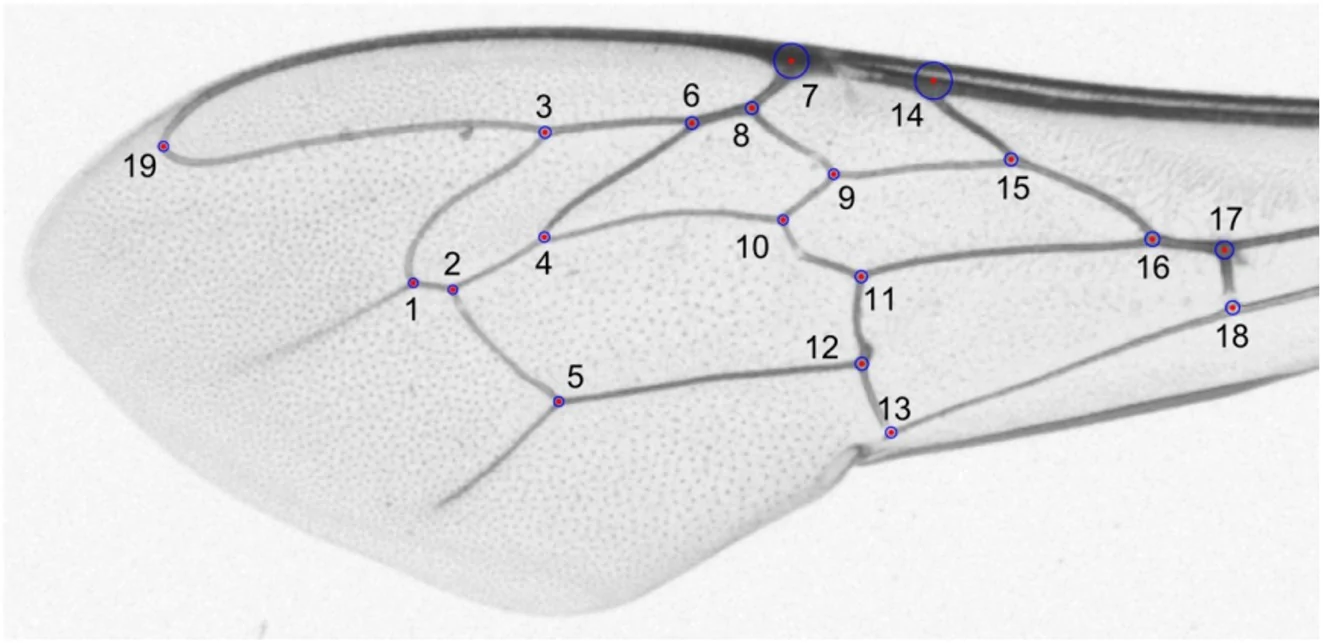
Position of landmarks on the forewing of a honey bee worker. The numbered dots indicate the landmarks. Circles surrounding the dots should be tangent to edges of surrounding venation.

The workers were stored until preparation in ethanol or in freezer. The wings were detached from the bees’ bodies, and most samples were dry mounted between 2 microscopic slides. Samples from the Netherlands were mounted using Entellan mounting medium. Measurements were taken from both the left and right forewings. If the side was known, it was indicated in the file name with the letters L and R for left and right wings, respectively. The differences between the left and right wings are relatively small, and their influence on geographic variation is negligible [69]. We used a variety of photographic equipment to acquire the images. Each wing was saved as a separate PNG file. Using the freely available IdentiFly software [61], the coordinates of 19 landmarks were determined in each wing image (Fig. 2). The configuration of landmarks is consistent with that used in earlier studies related to the identification of honey bee lineages and geographic variation in Europe and Asia [68, 70]. This enabled us to classify the samples as lineages and extend our dataset with existing data. These landmarks were saved within each wing image file and can be viewed and edited using IdentiFly. The complete dataset, including wing images, landmark coordinates, geographic coordinates of the sampling locations, and supplementary information, is available on the Zenodo website [71].

### Statistical analysis

A statistical analysis was performed using the R statistical computing environment (version 4.5.0) and the RStudio integrated development environment (version 2025.04.11). The landmarks were analyzed using geometric morphometric methodology. The raw landmark coordinates were aligned using the generalized Procrustes method, which was implemented in the geomorph package (version 4.0.10; [72]). The aligned coordinates were averaged within colonies, and these averages were used in subsequent analyses. Principal component analysis (PCA) was used to reduce the dimensionality of the wing shape data by extracting the first 34 principal components. Because PCA is an unsupervised method, it identifies directions of maximum overall variance without considering group membership. The principal component scores were subsequently used as input for linear discriminant analysis (LDA) implemented in the Morpho package (v. 2.13) [73]. In contrast to PCA, LDA is a supervised method that uses predefined group labels and identifies linear combinations of variables that reveal differences among groups. To evaluate the ability of the model to discriminate among groups while minimizing the risk of overfitting, classification performance was assessed using leave-one-out cross-validation. In this procedure, each specimen was excluded from the dataset in turn, the linear discriminant model was fitted using all remaining specimens, and the excluded specimen was subsequently classified using the resulting model. The differences in wing shape between countries were described using Mahalanobis distance. The samples were classified as evolutionary lineages using the IdentiFlyR package (v. 0.1.1) [74]. Data from Nawrocka et al. [61] were used as reference samples for the lineage. A supplementary document containing the full details of the statistical analysis is available on the GitHub website.

## Results

**Figure 3:**
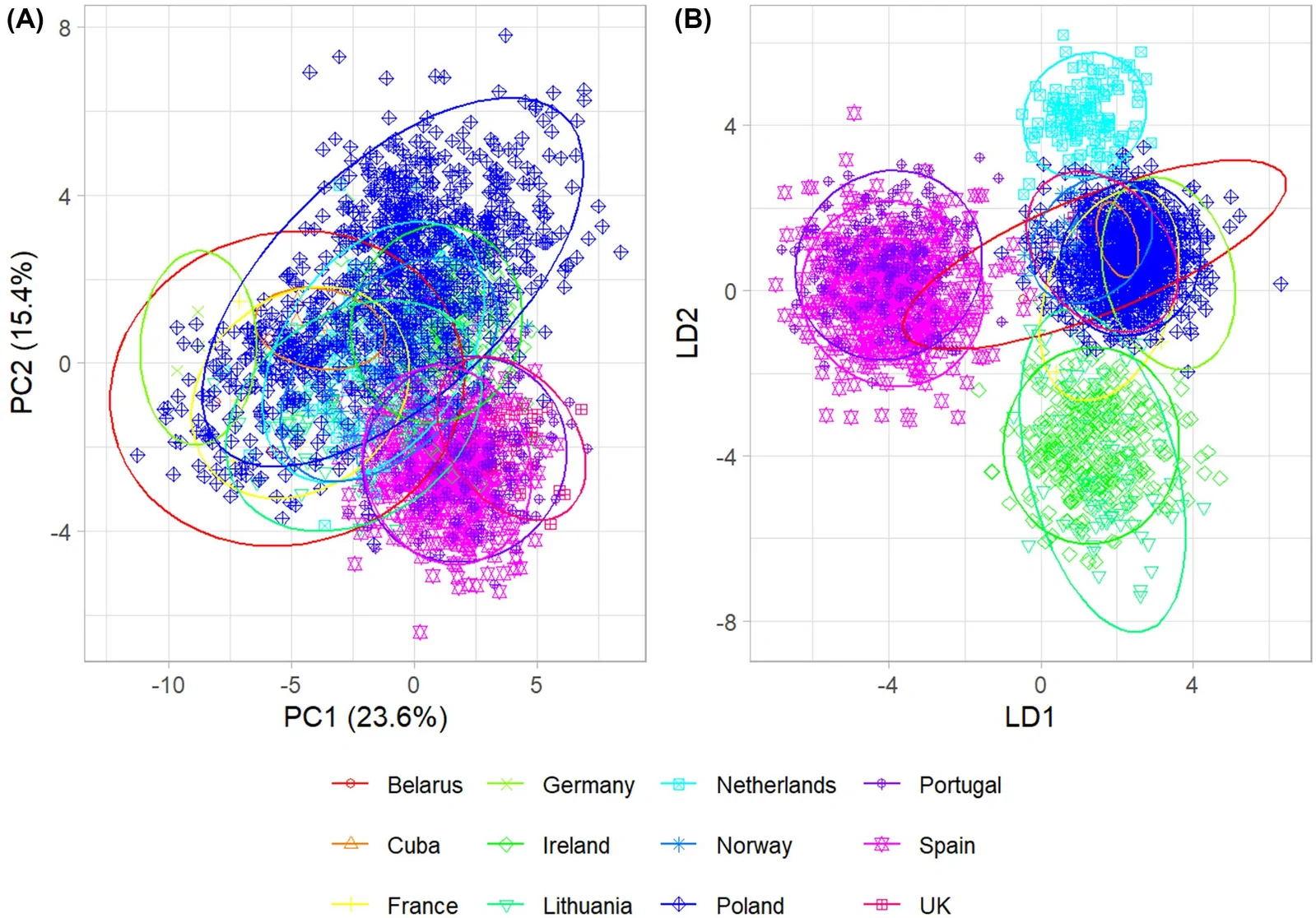
The first 2 principal components (A) and the first 2 linear discriminants (B) of honey bee wing shape in northwestern Europe. Each marker represents one colony. Ellipses indicate 95% confidence regions.

A PCA of wing shape revealed that honey bee colonies from northwestern Europe formed a single cluster. However, the 12 countries were somewhat separated along the first principal component, which explained 23.4% of the variance (Fig. 3A). The scatterplot showed considerable overlap among the groups, indicating a degree of morphological similarity. The LDA better separated the groups. The graph of the first 2 linear discriminants shows 3 clusters: the first consisted of Spain and Portugal; the second, of Ireland and Lithuania; and the third, of the remaining countries (Fig. 3B). Countries that overlapped in the graphs of the first 2 dimensions differed in other dimensions, and most countries differed markedly from each other in pairwise comparisons (Table 2). The largest Mahalanobis distance was found between samples from Cuba and the UK, and the smallest distance was found between samples from Portugal and Spain (Table 2). Using cross-validation for classification allowed us to correctly assign 89% of the samples to their respective countries. Most of the misclassifications occurred between neighboring countries, such as Portugal and Spain. However, misclassifications also occurred between distant countries, such as France and Belarus. For more details, see the supplementary document on GitHub.

**Table 2:** Differences between countries in wing shape (expressed as Mahalanobis distances, lower triangle) and significance of pairwise comparisons (upper triangle)

Country	BY	CU	DE	ES	FR	UK	IE	LT	NL	NO	PL	PT
BY		**	*	****	***	****	****	****	****	**	*	****
CU	6.617		****	****	****	****	****	****	****	****	****	****
DE	6.796	9.350		****	*	****	****	**	****	****	**	****
ES	6.832	8.722	9.241		****	****	****	****	****	****	****	****
FR	6.416	9.002	5.457	7.835		****	****	**	****	****	****	****
UK	8.406	10.059	9.826	8.897	9.487		****	****	****	****	****	****
IE	8.247	9.527	7.122	8.076	5.391	8.931		****	****	****	****	****
LT	7.187	9.641	5.724	7.742	3.648	8.188	3.943		****	****	****	****
NL	6.808	8.174	9.346	8.053	8.155	9.297	9.347	8.397		****	****	****
NO	5.451	7.346	8.134	6.837	6.253	7.230	7.363	6.750	6.623		****	****
PL	4.722	6.548	5.757	6.318	6.008	7.338	6.647	6.215	6.595	5.034		****
PT	7.190	8.925	9.601	1.701	8.518	8.957	8.653	8.304	7.964	7.102	6.425	

Country abbreviations: Belarus (BY), Cuba (CU), Germany (DE), Spain (ES), France (FR), the United Kingdom (UK), Ireland (IE), Lithuania (LT), the Netherlands (NL), Norway (NO), Poland (PL), and Portugal (PT). Asterisks denote statistical significance: *P* < 0.05 (*), *P* < 0.01 (**), *P* < 0.001 (***), and *P* < 0.0001 (****).

**Figure 4:**
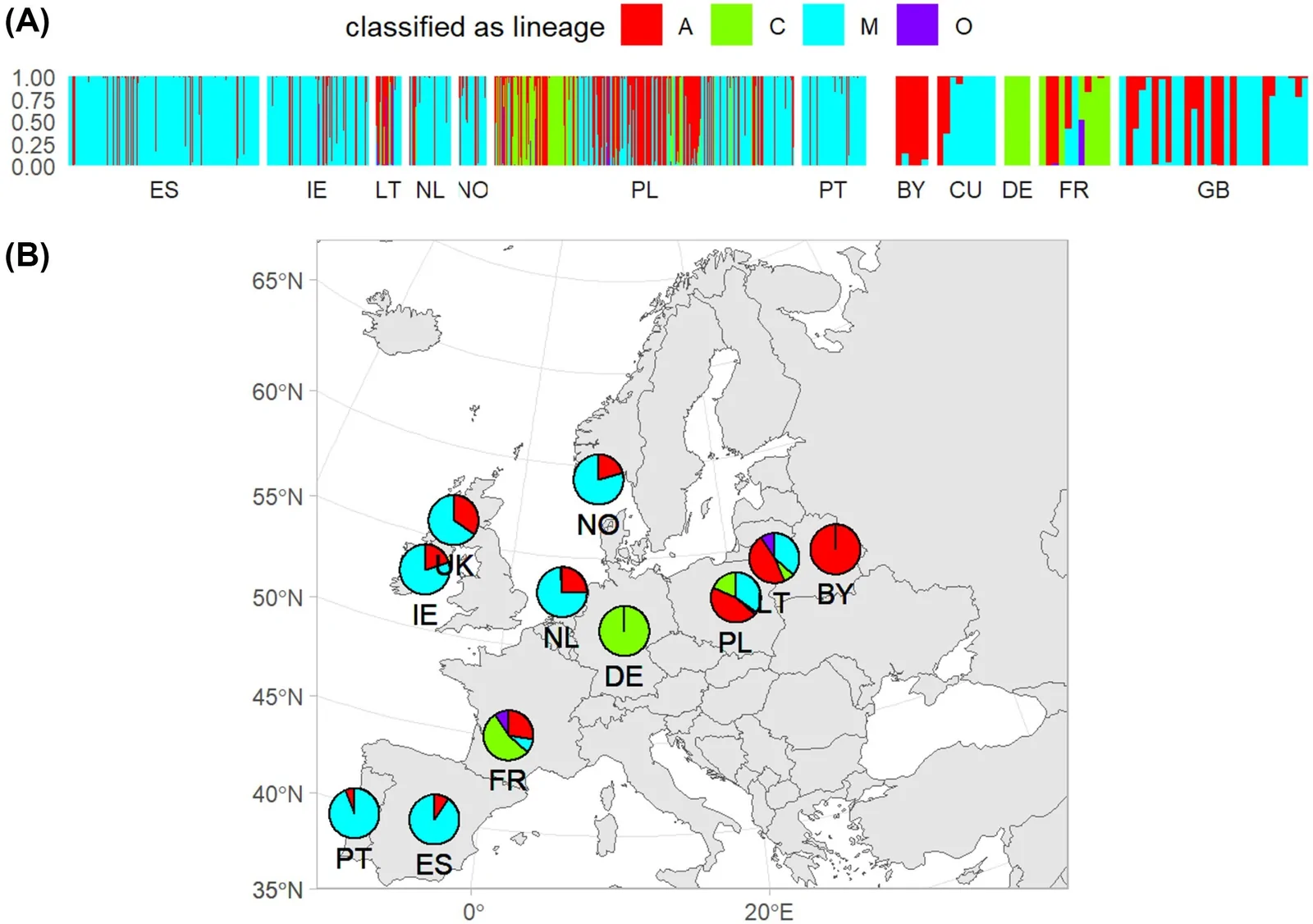
Classification of colonies into 4 evolutionary lineages, as shown in the admixture graph (A) and map (B). Country abbreviations are the same as in Table 2. Map created using the rnaturalearth R package.

Using reference samples from the Morphometric Bee Database in Oberursel, the colonies were classified into 4 evolutionary lineages: A, C, M, and O. Most colonies (63.7%) were classified as lineage M. Many were also classified as lineage A (27.5%), C (7.9%), or O (0.8%) (see Fig. 4). Lineage M, which is native to most of the study area, remains prevalent in Portugal (93.8%), Spain (90.8%), Norway (79.8%), Ireland (79.6%), and the Netherlands (73.6%) (Fig. 4). This indicates that conserving honey bee biodiversity within lineage M is feasible. We also found many signs of introgression. Lineage A, native to Africa, was dominant in Belarus and Poland. Lineage C and its hybrids, which are preferred by beekeepers, were dominant in Germany and France.

**Figure 5:**
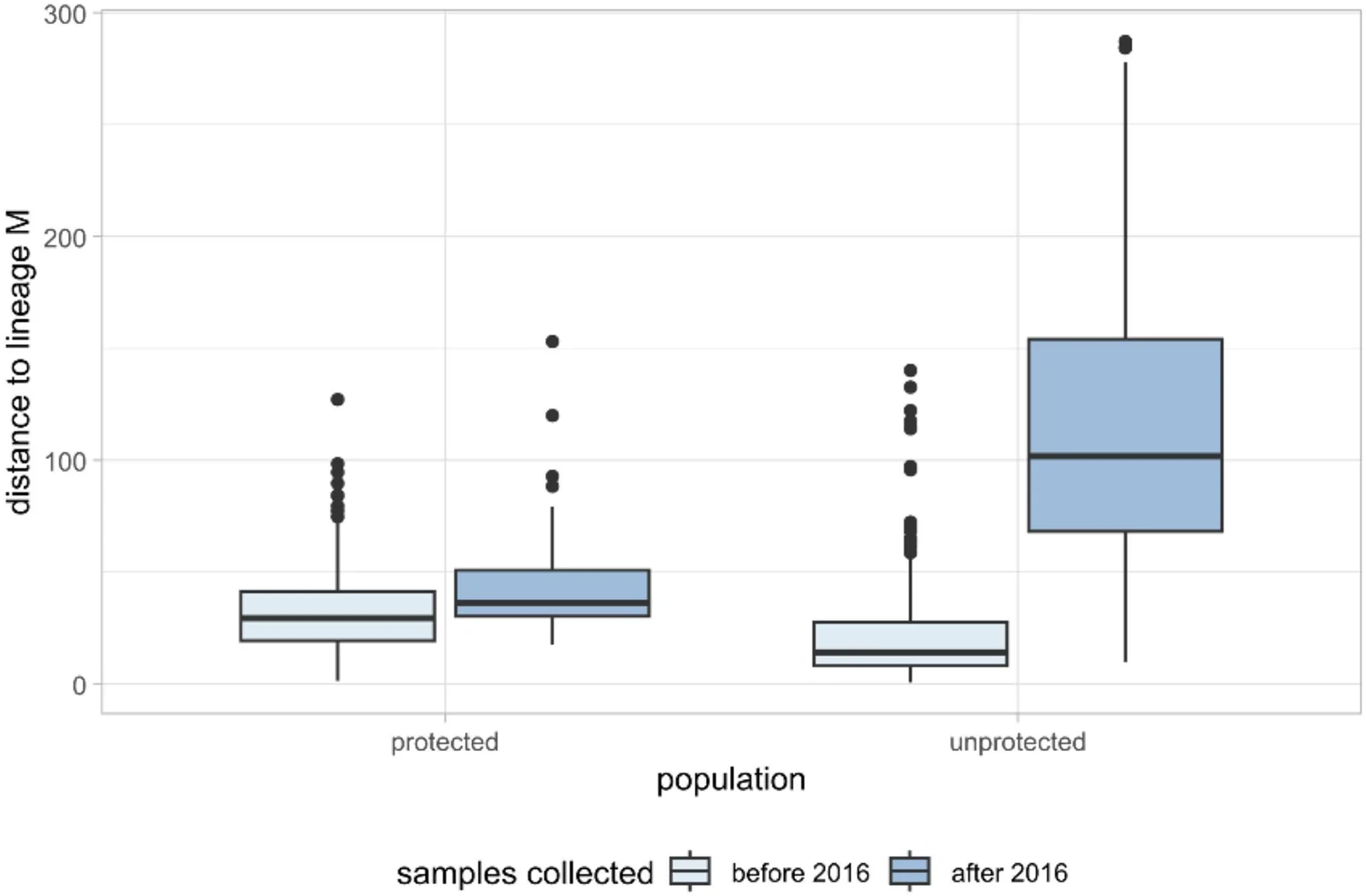
Mahalanobis distance to lineage M for honey bee samples collected in northeastern Poland before and after 2016. The box represents the interquartile range (25th–75th percentiles), the horizontal line indicates the median, the whiskers extend to the most extreme observations within 1.5× the interquartile range, and points denote outliers.

A large amount of data from both protected and unprotected populations in northeastern Poland allows us to investigate the temporal changes in the native honey bee lineage M. Since the beginning of the 21st century, the mean Mahalanobis distance to lineage M increased markedly in northeastern Poland (ANOVA; years: *F*_1,896_ = 652.87, *P* < 10^−15^; population: *F*_1,896_ = 41.51, *P* = 10^−10^; interaction: *F*_1,896_ = 197.69, *P* < 10^−15^). This increase was significant in both protected and unprotected populations, but particularly strong in the unprotected population (Fig. 5). The smaller the similarity to native bees, the larger the distance to lineage M. Therefore, honey bee samples collected after 2016 were significantly less similar to native bees than samples collected prior to that year (Fig. 5).

## Discussion

The data presented here show that the proportion of lineage M in northwestern Europe differed markedly between countries (Fig. 4). In Portugal and Spain, more than 90% of colonies are of the native lineage. Samples in this region were collected from unprotected populations, showing that beekeepers use native honey bees. This is the optimal solution for protecting honey bee diversity. When natural nesting sites for honey bee colonies become scarce, beekeepers provide hives for the bees without importing non-native bees. In this region, queen breeders do not use artificial insemination [75]. This allows them to maintain a large population of honey bees that are adapted to the local environment. At the same time, the beekeeping industry in this region is thriving, and honey production is the highest in Europe [76]. A high level of similarity to lineage M is also present in the unprotected population of Ireland [47–50, 77]. However, introgression from non-native lineages is larger in this country than in the Iberian Peninsula. In the Netherlands (Texel Island), the UK (Colonsay Island), and partly Norway, protected populations were sampled. In these populations, lineage M is well represented; however, in unprotected populations in these countries, lineage M is most likely much less prevalent [38]. In Poland and Lithuania, where both protected and unprotected populations were sampled, the proportion of lineage M is relatively low, but still present. In Lithuania, a relatively high proportion of colonies were classified as lineage O, which confirms earlier studies [78]. In the unprotected population of France that was sampled, only one of 11 colonies was classified as lineage M; lineage C dominates in this country, though all other lineages are present. An earlier molecular survey in France found a higher proportion of lineage M in both protected and unprotected populations [54]. However, another study revealed a relatively low percentage of lineage M in unprotected populations [79]. None of the colonies investigated in unprotected populations of Belarus were classified as lineage M, although admixture of lineage M was detected in some colonies (Fig. 5A). All 5 of the colonies investigated in Belarus were classified as lineage A, which occurs naturally in Africa. The mitochondrial DNA characteristic of lineage A is naturally present in the Iberian Peninsula [42], but not in northeastern Europe. A recent mitochondrial DNA survey in Central Europe detected African-ancestry haplotypes at a much lower frequency than reported here [37, 40, 80]. The high proportion of samples assigned to lineage A may be related to the fact that hybrids between lineages are more likely to be classified as lineage A [68]. These hybrids exhibit an intermediate phenotype similar to the mean shape of all lineages [81], and they are more effectively detected when molecular markers are used.

In Germany, all of the investigated colonies belonged to lineage C (Fig. 4). These bees originated from free-living colonies in a national park [63]. The process of replacing *A. m. mellifera* with *A. m. carnica* intensified in Germany in the 1940s [82]. By the end of the 20th century, this process had advanced markedly, with most colonies representing hybrids between the 2 subspecies [82]. Our data suggest that, in some parts of Germany, the replacement of the native subspecies by lineage C is now complete (Fig. 5A). A similar situation occurs in the Czech Republic, where all but a few colonies were classified as *A. m. carnica* [36, 37]. The data presented here indicate that *A. m. mellifera* is critically endangered in most of its native range. Surprisingly, lineage M is better preserved in Cuba, where *A. mellifera* is not native, than in most of its native range in northwestern Europe [66, 83].

There is significant geographic variability in the wing shape of honey bees in northwestern Europe. Within lineage M, 2 European subspecies were declared: *A. m. iberiensis* and *A. m. mellifera* [16]. The Pyrenees Mountains, a natural barrier to gene flow, lie between the 2 subspecies. Our results confirm the distinction between these 2 subspecies. Interestingly, mainly *A. m. mellifera* suffers from introgression with non-native bees. Significant differences in wing shape were also detected among countries within this subspecies, consistent with patterns previously identified using molecular markers [28]. This diversity may be related to natural variation shaped by natural selection [16] as well as beekeeping practices, including the introduction of commercial honey bees through breeding programs, importing queens, and transhumance movements [11]. Understanding which part of this variation is natural or human-induced is important for the adoption of optimal conservation strategies. Given the very large distribution area of *A. m. mellifera* and the various climatic conditions within it, it is highly probable that numerous ecotypes adapted to the local environment were present [27]. Some of these ecotypes, such as those occurring naturally in Germany and the Czech Republic, could be irreversibly lost. The best conservation strategy is probably to protect local bees, even if they differ from the historic populations of a particular area. According to this strategy, it is better to avoid the long-distance reintroduction of *A. m. mellifera*, as they most likely represent different ecotypes.

Some conservation strategies rely on artificial selection for phenotypic traits considered characteristic of native honey bee populations. For example, colonies may be selected based on the cubital index, a ratio of the lengths of 2 wing veins [84]. However, such an approach targets only the selected trait, while many other characteristics that contribute to local adaptation may not be maintained. This limitation is not unique to morphometric approaches. A similar problem arises when conservation is based on a limited number of genetic markers, as selection for those markers alone does not preserve the remainder of the genome that may contribute to adaptation to local environmental conditions.

A more effective strategy for conserving local *A. m. mellifera* ecotypes can be achieved by designating large areas where the introduction of non-local bees is prohibited [52]. Within such areas, natural selection can maintain genotypes that are adapted to local environmental conditions. Wing morphometry can support this strategy by providing an effective method for identifying colonies that have been recently introduced from other country. The selective removal of such colonies would help preserve the integrity of local populations without imposing artificial positive selection for a predefined set of morphological or genetic characteristics.

It is essential to describe the current variation and monitor how it changes. Originally, morphometry was used to describe the geographic variation of honey bees and differentiate subspecies. Later, molecular markers were introduced, improving subspecies discrimination. However, obtaining reference samples for molecular studies can be problematic in some parts of Europe [79] because populations are admixed, making it unclear which aspects of geographic variation are natural and which are related to beekeeping activities. In these cases, morphometric data can be used for cross-validation. Unlike molecular markers, morphometry is accessible to beekeepers; therefore, it can serve as a quick, inexpensive method for monitoring endangered populations. An important source of reference data is the Morphometric Bee Data Bank in Oberursel, Germany. Unfortunately, the currently available lineage data is limited in size [61], and further studies are needed to expand upon it. The data provided here can be used for future comparisons.

We used data from northeastern Poland to analyze changes in similarity to lineage M in recent times. This region was the last refuge of *A. m. mellifera* in Poland. The results are alarming, as the similarity to lineage M decreased markedly in the unprotected population (Fig. 5). The situation is much better in the protected population, which indicates that conservation programs are essential for the protection of the remaining *A. m. mellifera* populations. The rapid changes in recent years demonstrate the urgent need for action to prevent further erosion of honey bee genetic diversity in northwestern Europe. As has been shown, the introduction of bees maladapted to local environment into new areas causes increased mortality rates and contributes to bee losses [3]. The process of replacement of local populations of honey bees is supported within the current European Union Common Agricultural Policy giving funding to “rationalising transhumance” (Regulation (EU) 2021/2115, Article 55). A much more responsible action would enforce a moratorium on long distance bee movement between honey bees of different subspecies to minimize further loss of genetic diversity and avoid colony losses.

## Availability of source code and requirements

Project name: Apis-mellifera-NW-Europe

Project homepage: https://github.com/DrawWing/Apis-mellifera-NW-Europe, including the analysis scripts, statistics, and visualizations generated from the project

Operating system(s): Platform independent

Programming language: R

Other requirements: R (v. 4.5.0), numerous R packages described in the Methods section

License: GPL-3.0

WorkflowHub: https://workflowhub.eu/workflows/2226

Bio.tools: apis-wings-nw-europe

## List of abbreviations

BY: Belarus, CU: Cuba, DE: Germany, ES: Spain, FR: France, IE: Ireland, LDA: linear discriminant analysis, LT: Lithuania, NL: the Netherlands, NO: Norway, PCA: principal component analysis, PL: Poland, PT: Portugal; UK: the United Kingdom.

## Supplementary Material

giag093_Authors_Response_To_Reviewer_Comments_original_submission

giag093_GIGA-D-26-00106_original_submission

giag093_GIGA-D-26-00106_revision_1

giag093_Reviewer_1_Report_original_submission Reviewer 1 -- 4/27/2026

giag093_Reviewer_2_Report_original_submission Reviewer 2 -- 8/25/2026

giag093_Reviewer_2_Report_revision_1 Reviewer 1 -- 4/28/2026

## Data Availability

The complete dataset, including wing images, landmark coordinates, geographic coordinates of sampling locations, and other data, is available on the Zenodo website [71].
