# Supplementary material for: Country specific hybridization of honey bees from lineage M
Source: Gigascience. 2026 Sep 15;15:giag093. doi: 10.1093/gigascience/giag093 (PMC13615543; doi:10.1093/gigascience/giag093)
Supplement: Supplementary file 1 [file giag093_authors_response_to_reviewer_comments_original_submission.pdf]

Author's Response To Reviewer Comments

July 22, 2026
Dear Editor,
We are grateful to the reviewers for their constructive comments and corrections. We have revised the manuscript according to most of their suggestions. We believe these changes have substantially improved the manuscript.
Please see our point-by-point responses to the reviewers below. We have indicated these responses with "Response:"

Editor comments:
In addition, please register any new software application in the bio.tools and SciCrunch.org databases to receive RRID (Research Resource Identification Initiative ID) and biotoolsID identifiers, and include these in your manuscript. Computational workflows should be registered in workflowhub.eu and the DOIs cited in the relevant places in the manuscript. These will facilitate tracking, reproducibility and re-use of your tool.
Response: We added section "Availability of Source Code and Requirements" (lines: 376-384) with information about this project, including: bio.tools and WorkflowHub.
Our earlier attempt to obtain RRID from scicrunch.org was rejected because our project is not "biomedical" and is not related to "antibodies, organisms, chemicals, plasmids or cell lines"

Reviewer #1:
The manuscript GIGA-D-26-00106, entitled "Country-specific hybridization of honey bees from lineage M," analyzes Apis mellifera samples from 12 countries and applies geometric morphometry (GM) of the wing to classify honey bees for discrimination purposes.
This manuscript addresses an important and timely topic in bee discrimination. However, several issues should be considered before a final decision is made.
Response: We appreciate the positive feedback.

The use of geometric morphometry (GM) for lineage discrimination (A, M, etc.) is appropriate and well established. However, in the absence of genomic validation on the same specimens, the assignment of individuals to evolutionary lineages remains indirect. Reliance on previously published classifications introduces uncertainty, particularly in the context of hybridization. I recommend that the authors either provide genetic validation for the analyzed samples or explicitly discuss this limitation and its potential impact on classification accuracy.
Response: We agree that genetic material would be an important part of the dataset; however, most of the wing images were collected many years ago and it is now impossible to obtain genetic material. We used morphometry for identification of evolutionary lineages. This is justified because honey bee subspecies and lineages were described using morphometric measurements rather than genetic markers. Despite the fact that the original description of honey bee subspecies was morphometric, most data which are now considered as a reference for genetic markers were not validated using morphometric methods. We believe that the material we provide can be used to improve cross-validation between morphometric and molecular studies. We mention the problem of molecular validation in lines 351-357.
We admit that genetic markers can be more effective in detection of hybrids and mention this in lines 308-310.

2. Figure 5. How can the sampling time categories ("before 2016" and "after 2016") be interpreted? How can temporal changes in protected and unprotected samples be evaluated from this figure?
Response: We explained the categories in figure 5. We replaced "years" with "samples collected". Additionally, we explained figure 5 in lines 270-272.

3. The observed hybridization patterns may be influenced not only by classification outcomes but also by spatial structure, landscape connectivity, and beekeeping practices. Natural dispersal via swarming is typically limited in range, whereas long-distance gene flow is often human-mediated. It would be important to discuss potential geographic or ecological barriers to dispersal, as well as management practices (e.g., migratory beekeeping) that may affect hybridization. Without considering these factors, the classification

remains largely descriptive.
Response: We agree that the observed geographic variation of honey bees in Europe can be shaped by both natural and human-induced processes. We explain this in lines 324–330.

4. The conclusion is unclear. PCA indicates substantial overlap between groups, whereas the same data appear to show separation under a supervised model. This discrepancy requires clarification. Please explain how the supervised model achieves separation despite the overlap observed in PCA, and provide details on model validation (e.g., cross-validation, accuracy, or overfitting control) to support its reliability.
Response: We explained the procedure for analyzing wing shape variation in lines 208-218. We added results of classification with cross-validation in lines 235-239.

5. The Methods section lacks critical detail. It is not clear which wing was analyzed (right, left, or both). Additionally, the rationale for selecting the 12 countries is not provided. What is the history and intensity of beekeeping in these regions, and how might this influence the results?
Response: Measurements were taken from both the left and right forewings. If the side was known, it was indicated in the file name with the letters L and R for left and right wings, respectively. The differences between the left and right wings are relatively small, and their influence on geographic variation is negligible (Janczyk & Tofilski 2021). We explain this in lines 180-183.
We have attempted to collect as much material from lineage M as possible. The main factor affecting geographic variation of honey bees is intensity of queen replacement. Other beekeeping practices are less important. We provide information if the samples originated from protected or unprotected populations.

6. There is also a substantial imbalance in sample sizes (e.g., ~900 samples from Poland versus ~5 from Belarus). This raises concerns regarding statistical robustness, potential bias in classification performance, and the reliability of conclusions for underrepresented groups. Please clarify how these issues were addressed and discuss the associated limitations.
Response: We agree that the sample sizes differ markedly between countries. We would like to analyze more samples, however, they are not available for us. We hope the dataset will be enlarged in future. We mentioned this problem in lines 359-361.

7. Furthermore, although a COLOSS standard morphometric protocol is available (Meixner et al., 2012), it is not referenced or discussed. The numbering and ordering of landmarks also appear to differ from this standard. Please clarify which protocol was followed and justify any deviations.
Response: Unfortunately, the configuration of landmarks used for geometric morphometrics of honey bees differs between studies. Meixner et al. (2013) explained this problem well. The website referenced in the figure caption (http://apiclass.mnhn.fr/) is no longer accessible, and the landmark configuration depicted in the figure was employed in only three publications, all of which were co-authored by Michel Baylac. The last of these publications was published ten years ago. Currently, there is no generally accepted landmark configuration. We used a landmark configuration consistent with earlier studies related to lineage classification and available data from earlier studies. We explain this in lines 186-189. Fortunately, the landmarks can be reordered relatively easily. Furthermore, we provide all wing images, which can be remeasured if necessary.

8. Line 328: Why do the authors conclude that maladapted bees introduced into new areas carry “poor” genes? This statement appears speculative and requires clarification and supporting evidence.
Response: We removed the fragment related to “poor genes.” We explained in lines 368-369 that the bees introduced by beekeepers are “maladapted to the local environment.”

Overall, I recommend major revisions, as several key methodological and interpretative issues need to be addressed to support the conclusions.
Response: Thank you for the recommendation, as well as the constructive comments and corrections.

Reviewer #2:
This is an important paper, as it provides evidence to counter the argument that the M lineage of honey bees no longer exists in many European locations.

The data reported in the paper are not a randomised survey, but combine existing published material and

new previously unpublished data, but with a patchy coverage, although Poland is comprehensively covered. The paper reports on some 29,000 images of wings which represent over 1,000 colonies from twelve countries. The images are now placed in a public repository, a valuable resource, as there are limited numbers of reference samples from many countries.

The results show that M Lineage is still prevalent in general bee populations in Ireland, Portugal, and Spain, but only exists in populations under some kind of protection in most other countries of its former range.
Response: We appreciate the positive feedback.

The paper explains that both molecular and morphometric techniques can be used to discriminate between populations, but notes that molecular techniques are not usually available to beekeepers, for whom morphometric techniques are more appropriate. There could perhaps be a comment added about divergence between the two techniques. What happens in beekeepers select populations on the basis of just a few wing morphometric characters? Maybe comment on this…
Response: We agree that selecting for a small number of traits characteristic of native bees is problematic. We explain this in lines 336-350. We point out that this problem is not limited to morphometry, but also applies to a small number of genetic markers. Therefore, we suggest that the best conservation strategy for honey bees is to designate protected areas to which non-native bees cannot be imported.

Sadly in many parts of its former area, such as Germany and the Czech Republic, A.m.m. may be effectively extinct, but the paper makes a plea for local beekeepers to work with locally adapted bees rather than trying to reintroduce A.m.m. from elsewhere, where the ecotypes are probably different from the extinct former populations. This is an important point.
Response: Thank you for the positive response and constructive comments.

Editorial Manager®

Cookie Preference Center

We use cookies which are necessary to make our site work. We may also use additional cookies to analyze, improve and personalize our content and your digital experience. For more information, see our Cookie Policy.

You may choose not to allow some types of cookies. However, blocking some types may impact your experience of our site and the services we are able to offer. See the different category headings below to find out more or change your settings.

Allow all

Manage Consent Preferences

Strictly Necessary Cookies

Always active

These cookies are necessary for the website to function and cannot be switched off in our systems. They are usually only set in response to actions made by you which amount to a request for services, such as setting your privacy preferences, logging in or filling in forms. You can set your browser to block or alert you about these cookies, but some parts of the site will not then work.

Functional Cookies

These cookies enable the website to provide enhanced functionality and personalisation. They may be set by us or by third party providers whose services we have added to our pages. If you do not allow these cookies then some or all of these services may not function properly.

Performance Cookies

These cookies allow us to count visits and traffic sources so we can measure and improve the performance of our site. They help us to know which pages are the most and least popular and see how visitors move around the site.

Targeting Cookies

These cookies may be set through our site by our advertising partners. They may be used by those companies to build a profile of your interests and show you relevant adverts on other sites. If you do not allow these cookies, you will experience less targeted advertising.

Cookie List

Clear

Apply Cancel

Consent Leg.Interest

Confirm my choices

Powe
