# Supplementary material for: Country specific hybridization of honey bees from lineage M
Source: Gigascience. 2026 Sep 15;15:giag093. doi: 10.1093/gigascience/giag093 (PMC13615543; doi:10.1093/gigascience/giag093)
Supplement: Supplementary file 2 [file giag093_giga-d-26-00106_original_submission.pdf]

GigaScience

Country specific hybridization of honey bees from lineage M

--Manuscript Draft--

**Manuscript Number:**	GIGA-D-26-00106
**Full Title:**	Country specific hybridization of honey bees from lineage M
**Article Type:**	Data Note
**Funding Information:**	Narodowe Centrum Nauki (2021/41/B/NZ9/03153) — Prof Adam Tofilski
**Abstract:**	Background<br><br>Honey bees are essential pollinators supporting agricultural production and wild plant diversity. In evolutionary lineage M, some populations are threatened by genetic erosion caused by the widespread introduction of commercially bred queens. To assess this risk, wing images from existing and new datasets were used to assign them to four evolutionary lineages (A, C, M, and O). The new dataset consisted of 29,043 wing images representing 1,342 colony samples from ten countries.<br><br>Results<br><br>Overall, 63.7% of colonies belonged to lineage M, whereas 27.5% were classified as A, 7.9% as C, and 0.8% as O. Lineage M remains prevalent in unprotected populations in Portugal, Spain, and Ireland, as well as in protected populations elsewhere. In contrast, a pronounced decline was observed in unprotected populations in northeastern Poland.<br><br>Conclusions<br><br>These findings reveal strong regional differences in the persistence of lineage M and underscore the need for coordinated conservation efforts throughout Europe. The data provided in this study should allow for more accurate discrimination between native and introduced phenotypes.
**Corresponding Author:**	Adam Tofilski<br>University of Agriculture in Krakow: Uniwersytet Rolniczy im Hugona Kollataja w Krakowie<br>Krakow, POLAND
**Corresponding Author Secondary Information:**	
**Corresponding Author's Institution:**	University of Agriculture in Krakow: Uniwersytet Rolniczy im Hugona Kollataja w Krakowie
**Corresponding Author's Secondary Institution:**	
**First Author:**	Julita Machlowska
**First Author Secondary Information:**	
**Order of Authors:**	Julita Machlowska
	Dariusz Gerula
	Andrzej Oleksa
	Henk Kok
	Grace Patricia McCormack
	Alexandra Valentine
	Lars Kirkerud
	Per Kryger
	Laima Blažytė-Čereškienė

Powered by Editorial Manager® and ProduXion Manager® from Aries Systems Corporation

	Martin Hasselmann
	Teweldemedhn Gebretinsae Hailu
	Benjamin Rutschmann
	Adam Tofilski
**Order of Authors Secondary Information:**	
**Additional Information:**	
**Question**	**Response**
Are you submitting this manuscript to a special series or article collection?	No
**Experimental design and statistics**<br><br>Full details of the experimental design and statistical methods used should be given in the Methods section, as detailed in our Minimum Standards Reporting Checklist. Information essential to interpreting the data presented should be made available in the figure legends.<br><br>Have you included all the information requested in your manuscript?	Yes
**Resources**<br><br>A description of all resources used, including antibodies, cell lines, animals and software tools, with enough information to allow them to be uniquely identified, should be included in the Methods section. Authors are strongly encouraged to cite Research Resource Identifiers (RRIDs) for antibodies, model organisms and tools, where possible.<br><br>Have you included the information requested as detailed in our Minimum Standards Reporting Checklist?	Yes
**Availability of data and materials**<br><br>All datasets and code on which the conclusions of the paper rely must be either included in your submission or	Yes

deposited in publicly available repositories (where available and ethically appropriate), referencing such data using a unique identifier in the references and in the “Availability of Data and Materials” section of your manuscript.<br><br>Have you have met the above requirement as detailed in our Minimum Standards Reporting Checklist?	
GigaScience has policies and guidelines in place for the use of generative AI-writing tools such as ChatGPT. If you have used such writing tools to assist with writing the manuscript this must be declared and cited in the text. Authors should not list AI-writing tools and other AI-assisted technologies as an author or co-author and should acknowledge that they are fully responsible for text generated or refined by AI-writing tools.<p><br><br>A summary of use (particularly in the introduction or among methods) needs to be included at the end of the paper, and the outputs should also be included as a supplementary file hosted in GigaDB or other open repositories. Please read our guidelines for more information. <p><br><br>By submitting to GigaScience, you are aware of the journal's AI-writing tools policy, and if you have declared use of such tools below, you have acknowledged this where appropriate in your manuscript and have made a summary of use and outputs available. <p><br><b>AI-assisted writing tools have been used in the preparation of this manuscript?	Yes

Manuscript

Click here to access/download;Manuscript;Apis-mellifera-NW-Europe-GigaScience-v1.docx

Country specific hybridization of honey bees from lineage M

Julita Machlowska[1] orcid: 0000-0001-9951-9248

Dariusz Gerula[2] orcid: 0000-0003-3875-2657

Andrzej Oleksa[3] orcid: 0000-0002-0414-8075

Henk Kok[4]

Grace P. McCormack[5] orcid: 0000-0003-4960-5185

Alexandra Valentine[5] orcid: 0009-0002-4748-3735

Lars Kirkerud[6]

Per Kryger[7] orcid: 0000-0001-5306-3230

Laima Blažytė-Čereškienė[8] orcid: 0000-0002-0801-0196

Martin Hasselmann[9] orcid: 0000-0003-3305-4024

Teweldemedhn Gebretinsae Hailu[9] orcid: 0000-0002-3043-995X

Benjamin Rutschmann[10,11] orcid: 0000-0001-6589-6408

Adam Tofilski[1] orcid: 0000-0002-3898-7029

1. Department of Zoology and Animal Welfare, University of Agriculture in Krakow, 29 Listopada 56, 31-425 Kraków, Poland, julita.machlowska@urk.edu.pl, rotofils@cyf-kr.edu.pl
2. Apiculture Division, National Institute of Horticultural Research, 24-100 Pulawy, Poland, dariusz.gerula@inhort.pl
3. Department of Genetics, Kazimierz Wielki University, Powstańców Wielkopolskich 10, 85-090 Bydgoszcz, Poland, olek@ukw.edu.pl
4. Nederlandse Bijenhoudersvereniging, Stationsweg 94A, 6711 PW, Ede, Netherlands, h.s.kok.veenendaal@gmail.com
5. Molecular Evolution and Systematics Laboratory, University of Galway, University of Galway, University Rd., H91 TK33 Galway, Ireland, grace.mccormack@universityofgalway.ie, a.valentine2@universityofgalway.ie
6. Norsk Brunbielag, Dampsagveien 14, 2004 Lillestrøm, Norway, lkirkerd@online.no
7. Department of Agroecology - Agricultural biodiversity, Aarhus University, Forsøgsvej 1, 4200 Slagelse, Denmark, per.kryger@agro.au.dk
8. Nature Research Centre, Akademijos St. 2, 08412 Vilnius, Lithuania, laima.blazyte@gamtc.lt
9. Department of Population Genomics, Institute of Animal Science, University of Hohenheim, Garbenstr. 17, 70599 Stuttgart, Germany martin.hasselmann@uni-hohenheim.de, teweldeg2008@gmail.com
10. Agroecology and Environment, Agroscope, Reckenholzstrasse 191, 8046 Zurich, Switzerland
11. Department of Animal Ecology and Tropical Biology, Biocenter, University of Würzburg, Würzburg, Germany, benjamin.rutschmann@uni-wuerzburg.de

Corresponding author: Adam Tofilski, Department of Zoology and Animal Welfare, University of Agriculture in Krakow, 29 Listopada 56, 31-425 Kraków, Poland, rotofils@cyf-kr.edu.pl

Abstract

Background

Honey bees are essential pollinators supporting agricultural production and wild plant diversity. In evolutionary lineage M, some populations are threatened by genetic erosion caused by the widespread introduction of commercially bred queens. To assess this risk, wing images from existing and new datasets were used to assign them to four evolutionary lineages (A, C, M, and O). The new dataset consisted of 29,043 wing images representing 1,342 colony samples from ten countries.

Results

Overall, 63.7% of colonies belonged to lineage M, whereas 27.5% were classified as A, 7.9% as C, and 0.8% as O. Lineage M remains prevalent in unprotected populations in Portugal, Spain, and Ireland, as well as in protected populations elsewhere. In contrast, a pronounced decline was observed in unprotected populations in northeastern Poland.

Conclusions

These findings reveal strong regional differences in the persistence of lineage M and underscore the need for coordinated conservation efforts throughout Europe. The data provided in this study should allow for more accurate discrimination between native and introduced phenotypes.

Key words: Apis mellifera, wing, geometric morphometrics, M-lineage bees, northwestern Europe

Data description

We provide 29,043 fore wing images of honey bee workers. They represent 1,342 samples from 10 countries. The shape of the wings was described using the coordinates for 19 landmarks at wing veins' intersections. The whole dataset, including the wing images, landmark coordinates, geographic coordinates of sampling locations, and other data, is available on the Zenodo website https://zenodo.org/records/18845767 [1].

Introduction

Honey bees (*Apis mellifera*) are valuable pollinators that contribute significantly to agricultural productivity and the maintenance of wild plant diversity. In agriculture, their pollination services are essential for many fruit, vegetable, and nut crops. The global annual economic value directly attributable to animal pollination is estimated to be in the hundreds of billions of U.S. dollars [2]. Beyond agriculture, honey bees contribute to the pollination of a vast array of wild plants, supporting ecosystem function and the reproduction of non-crop flora, which is essential for broader biodiversity [3]. The resilience of these pollination services is crucially linked to genetic diversity within honey bee populations. Subspecies and ecotypes adapted to specific climatic and floral conditions often have superior survival rates in their native ranges compared to non-adapted, commercially imported stocks [4,5]. Managed honey bee populations in the USA and parts of Europe have experienced significant declines, often categorized as colony collapse disorder [6,7], and evidence also indicates declines in wild-living honey bee populations across much of Europe [8]. Apart from primary drivers such as pathogens and agrochemicals, the loss of population genetic variability and the erosion of adaptation to the local environment have been suggested as causes of the decline [9–11]. Therefore, preserving the intraspecific biodiversity of honey bees is a conservation imperative and a practical necessity for sustaining robust pollination services and agricultural security in the face of environmental change [12,13].

The natural distribution of *Apis mellifera* includes Africa, Europe, and Western Asia; however, this species is found worldwide due to multiple introductions [14,15]. Approximately 30 honey bee subspecies have been identified [16–19]. Four major evolutionary lineages are recognized within the species: the African lineage (A), the Central and Eastern European lineage (C), the Western and

Northern European lineage (M), and the Middle Eastern and Central Asian lineage (O) [17,20]. Recently, honey bees from Ethiopia, originally classified as lineage A, were reclassified as lineage Y using molecular and wing morphometric analyses [21]. Another lineage, lineage Z, was identified in Syria and Lebanon [22,23].

Lineage M's natural distribution extends from Ireland and Portugal in the west to the Ural Mountains in Russia in the east [17]. Recently, it was suggested that lineage M also occurs naturally in western China [24]. The northernmost limit of the natural distribution of *A. mellifera* and its M lineage is southern Scandinavia, around 60° N [25]. The southernmost limits of lineage M extend along the Mediterranean coast of Spain and France (including Corsica) and the northern slopes of the Alps. Further east, there was a hybrid zone between lineages M and C, which was less clearly defined. It was assumed that lineage M occurred in northern Austria, the Czech Republic, most of Poland, and the northern part of Ukraine [17,26]. Within this distribution, three subspecies were distinguished: *A. mellifera iberiensis* in the Iberian Peninsula; *A. mellifera mellifera* in the rest of the European distribution of lineage M; and *A. mellifera sinisxinyuan* in western China [24,27,28]. Geographic variation within the subspecies is represented by distinct ecotypes [5,12,26,28,29].

In some parts of Europe, native honey bee populations were significantly influenced by human management, including the importation of queens, transhumance movements, and breeding programs [10,12,30,31]. Since the early 20th century, commercial beekeeping has favored non-native subspecies from lineage C, particularly *A. mellifera carnica* and *A. mellifera ligustica*, as well as their hybrids. Beekeepers widely perceived these subspecies as more suitable for apiculture than the native *A. m. mellifera* or found that hybrids between their original M-lineage bees and introduced bees were more productive due to short-term heterosis effects [32,33]. The extensive gene flow between native populations and commercially propagated bees has led to the disappearance or significant genetic erosion of indigenous honey bees in many European regions [34]. In Germany, for instance, the large-scale introduction of non-native queens led to the widespread replacement of *A. m. mellifera* with *A. m. carnica* [35,36]. Similar patterns of replacement or strong introgression have been documented in the Czech Republic [37,38], Denmark [39], and parts of Poland [40,41]. Currently, in many regions of Europe, most professional and amateur beekeepers maintain *A. m. ligustica*, *A. m. carnica*, or their hybrids. Consequently, the natural distribution range of lineage M, particularly *A. m. mellifera*, has substantially decreased in recent decades [11,42], and it continues to be threatened by further introgression.

The degree of genetic introgression varies considerably across northwestern Europe. While many regions exhibit high levels of admixture with C-lineage bees, several areas with relatively low levels of introgression have been identified. These regions include the Iberian Peninsula [43–47], Ireland [48–51], the Inner Hebrides [52,53], northeastern Poland [54], and some protected areas in the Netherlands, Norway, France, Denmark, and Switzerland [26,52,53,55,56]. These regions are therefore considered important genetic refuges for lineage M.

Conserving honey bees is crucial for maintaining their genetic diversity and local adaptations to different environments. Global concern over the loss of honey bee genetic resources has grown in recent years [42], prompting the development of multiple conservation initiatives across Europe [12,57]. One of the most prominent efforts is the International Association for the Conservation of the European Dark Honey Bee (SICAMM), which was established in 1994. SICAMM unites beekeepers, researchers, and conservationists in support of preserving and promoting *A. m. mellifera* across its historical range [58]. Effective conservation management requires reliable, cost-effective, and practical tools to detect introgression between honey bee subspecies. These methods are essential for monitoring genetic integrity, guiding breeding programs, and preventing further introgression in conservation areas. Contemporary methodologies for identifying honey bee subspecies are based on two contrasting approaches: molecular markers and morphometry [28]. Molecular markers, such as

microsatellites [59] and, more recently, highly accurate single nucleotide polymorphisms [29,52,60], constitute a powerful identification toolkit. However, beekeepers' use of these methods is severely limited due to the need for specialized personnel, costly equipment and reagents, and the lack of rapid accessibility, which makes routine colony analysis unaffordable. In contrast, morphometric identification based on wing venation [17,28,61] is a highly cost-effective alternative that can be more easily implemented in apiaries. This approach requires only a microscope with an attached camera to deliver a practical, affordable tool for beekeepers. Morphometric identification of honey bee subspecies is relatively easy using freely available software [58,62]; however, it requires reference datasets for comparison. Unfortunately, access to publicly available reference data is limited. Paradoxically, there is much more reference data for *A. m. iberiensis* [63] — a well-protected subspecies — than for *A. m. mellifera* [62], an endangered subspecies.

We provide a large amount of data on wing variation in northwestern Europe that can be used to monitor local populations in the future. Furthermore, we demonstrate that the hybridization process of *A. m. mellifera* has accelerated recently, and urgent action is required to prevent the complete extinction of this subspecies.

Country	Number of wings	Number of samples
Belarus	171	5
Cuba	449	9
Germany	60	4
Spain	3482	573
France	164	11
United Kingdom	425	29
Ireland	3498	304
Lithuania	1358	76
Netherlands	3555	125
Norway	2274	84
Poland	22574	900
Portugal	960	192

Table 1. Sample size of honey bee wings used in this study.

Methods

Material

In this study, we provide 29,043 images of honey bee worker wings in a public repository, which is a new contribution. These images represent 1,342 samples from ten countries. Some of these samples were related to previous studies: samples 1–4 from Germany [64]; samples 517–537 from Spain [47]; samples 1–304 from Ireland [51]; samples 640–679 and 680–922 from Poland [65,66]. Material from Belarus, France, United Kingdom, Lithuania, Netherlands, Norway and most material from Poland was not presented in earlier publications. Samples from Belarus were collected in 2016 from log hives in an unprotected population. Samples from France were collected from both log hives and managed hives, which originated from an unprotected population. Samples from the United Kingdom were collected from the protected population on Colonsay island in 2014. Samples from Lithuania were collected from small, traditionally managed apiaries situated in forested regions and remote from apiaries practicing modern beekeeping. Samples from the Netherlands were collected in 2024 from the protected population on Texel island. Samples from Norway were collected from the Flekkefjord conservation area (29 colonies) and other unprotected parts of the country (55 colonies). Most samples from Poland originated from protected populations, which are maintained by honey bee breeders using instrumental insemination. The number of workers per sample in the new dataset ranged from 5 to 69, with an average of 21.6. We also remeasured 449 wings representing nine colonies from Cuba [67,68] and used data from earlier studies related to

Poland, Portugal, and Spain [63,69]. In total, we used 38,990 wing images, representing 2,312 samples and 12 countries, in the analysis (Table 1, Fig. 1).

The workers were stored until preparation in ethanol or in freezer. The wings were detached from the bees' bodies and most samples were dry mounted between two microscopic slides. Samples from Netherlands were mounted using Entellan mounting medium. We used a variety of photographic equipment to acquire the images. Each wing was saved as a separate PNG file. Using the freely available IdentiFly software (http://drawwing.org/identifly) [62], the coordinates of 19 landmarks were determined in each wing image (Fig. 2). These landmarks were saved within each wing image file and can be viewed and edited using IdentiFly. The complete dataset, including wing images, landmark coordinates, geographic coordinates of the sampling locations, and supplementary information, is available on the Zenodo website https://zenodo.org/records/18845767 [1].

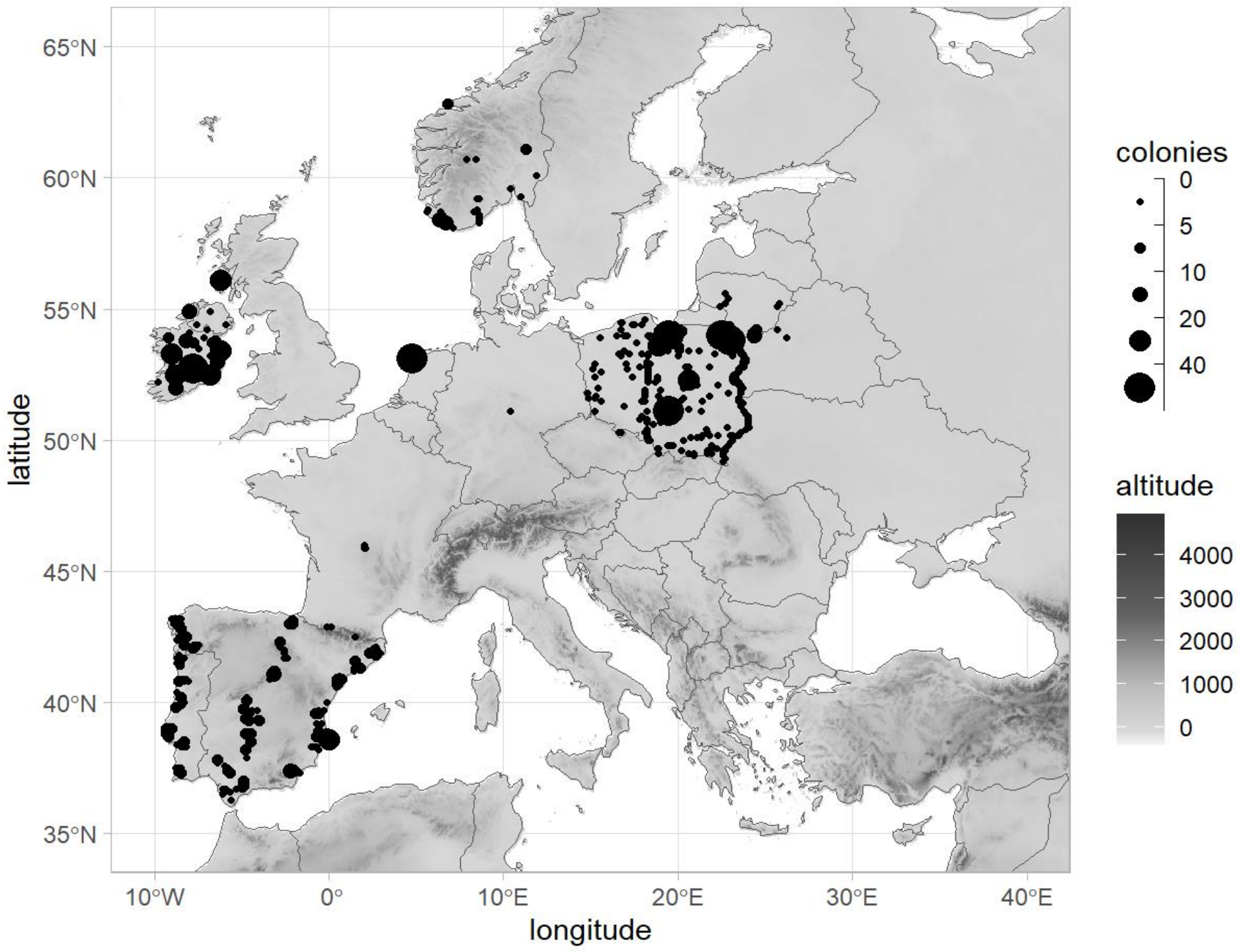

Figure 1. Locations from which honey bee samples were collected.

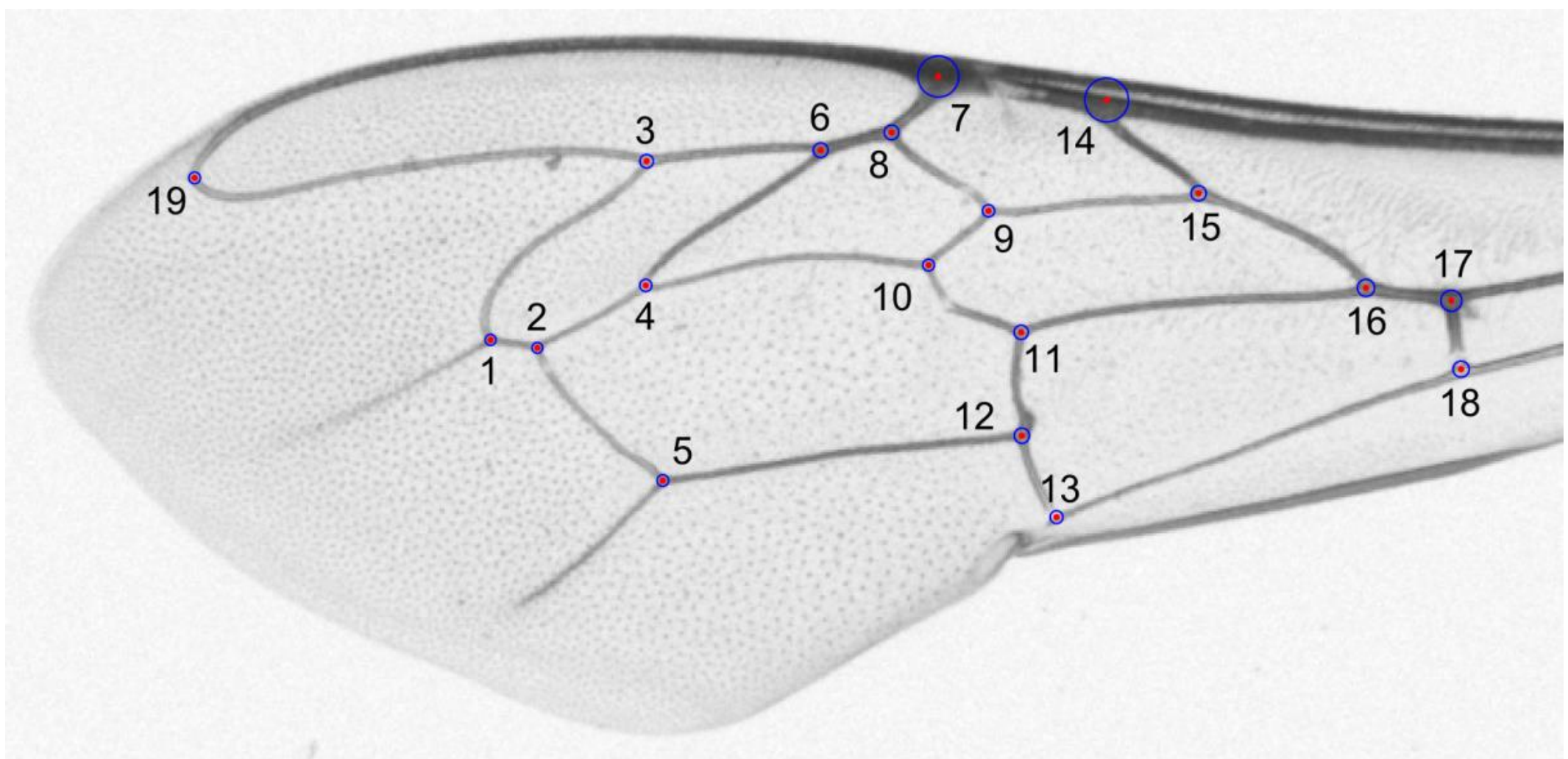

Figure 2. Position of landmarks on the forewing of a honey bee worker. The numbered red dots indicate the landmarks. Blue circles surrounding the dots should be tangent to edges of surrounding venation.

Statistical analysis

A statistical analysis was performed using the R statistical computing environment (version 4.5.0) and the RStudio integrated development environment (version 2025.04.11). The landmarks were analyzed using geometric morphometric methodology. The raw landmark coordinates were aligned using the generalized Procrustes method, which was implemented in the geomorph package (version 4.0.10; [70]. The aligned coordinates were averaged within colonies, and these averages were used in subsequent analyses. Principal component analysis was used to extract the first 34 principal components to describe wing shape. Linear discriminant analysis was used to calculate the differences between countries using the Morpho package (v. 2.13) [71]. The differences in wing shape between countries were described using Mahalanobis distance. The samples were classified as evolutionary lineages using the IdentiFlyR package (v. 0.1.1) [72]. Data from Nawrocka et al. [62] was used as reference samples for the lineage.

Results

A principal component analysis of wing shape revealed that honey bee colonies from northwestern Europe formed a single cluster. However, the twelve countries were somewhat separated along the first principal component, which explained 23.4% of the variance (Fig. 3a). The scatterplot showed considerable overlap among the groups, indicating a degree of morphological similarity. The linear discriminant analysis better separated the groups. The graph of the first two linear discriminants shows three clusters: the first consisted of Spain and Portugal; the second, of Ireland and Lithuania; and the third, of the remaining countries (Fig. 3b). Countries that overlapped in the graphs of the first two dimensions differed in other dimensions, and most countries differed markedly from each other in pairwise comparisons (Table 2). The largest Mahalanobis distance was found between samples from Cuba and the United Kingdom, and the smallest distance was found between samples from Portugal and Spain (Table 2).

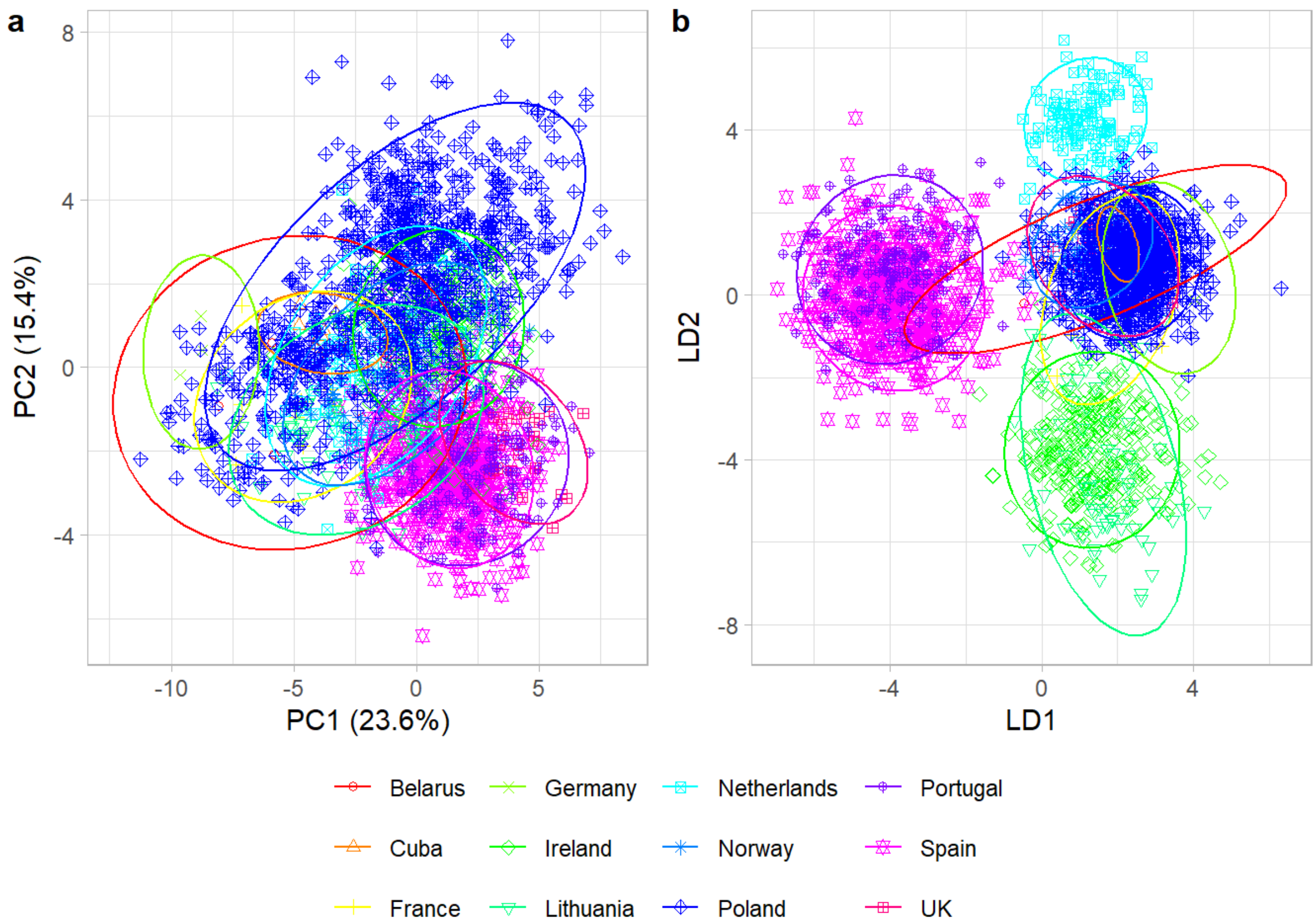

Figure 3. The first two principal components (a) and the first two linear discriminants (b) of honey bee wing shape in northwestern Europe. Each marker represents one colony. Ellipses indicate 95% confidence regions.

Country	BY	CU	DE	ES	FR	UK	IE	LT	NL	NO	PL	PT
BY	-	**	*	****	***	****	****	****	****	**	*	****
CU	6.617	-	****	****	****	****	****	****	****	****	****	****
DE	6.796	9.350	-	****	*	****	****	**	****	****	**	****
ES	6.832	8.722	9.241	-	****	****	****	****	****	****	****	****
FR	6.416	9.002	5.457	7.835	-	****	****	**	****	****	****	****
UK	8.406	10.059	9.826	8.897	9.487	-	****	****	****	****	****	****
IE	8.247	9.527	7.122	8.076	5.391	8.931	-	****	****	****	****	****
LT	7.187	9.641	5.724	7.742	3.648	8.188	3.943	-	****	****	****	****
NL	6.808	8.174	9.346	8.053	8.155	9.297	9.347	8.397	-	****	****	****
NO	5.451	7.346	8.134	6.837	6.253	7.230	7.363	6.750	6.623	-	****	****
PL	4.722	6.548	5.757	6.318	6.008	7.338	6.647	6.215	6.595	5.034	-	****
PT	7.190	8.925	9.601	1.701	8.518	8.957	8.653	8.304	7.964	7.102	6.425	-

Table 2. Differences between countries in wing shape (expressed as Mahalanobis distances, lower triangle) and significance of pairwise comparisons (upper triangle). Country abbreviations: Belarus (BY), Cuba (CU), Germany (DE), Spain (ES), France (FR), the United Kingdom (UK), Ireland (IE), Lithuania (LT), the Netherlands (NL), Norway (NO), Poland (PL), Portugal (PT). Asterisks denote statistical significance: $p < 0.05$ (*), $p < 0.01$ (**), $p < 0.001$ (***), and $p < 0.0001$ (****).

Using reference samples from the Morphometric Bee Database in Oberursel, the colonies were classified into four evolutionary lineages: A, C, M, and O. Most colonies (63.7%) were classified as lineage M. Many were also classified as lineage A (27.5%), C (7.9%), or O (0.8%) (see Fig. 4). Lineage M, which is native to most of the study area, remains prevalent in Portugal (93.8%), Spain (90.8%), Norway (79.8%), Ireland (79.6%), and the Netherlands (73.6%) (Fig. 4). This indicates that conserving honey bee biodiversity within lineage M is feasible. We also found many signs of introgression. Lineage A, native to Africa, was dominant in Belarus and Poland. Lineage C and its hybrids, which are preferred by beekeepers, were dominant in Germany and France.

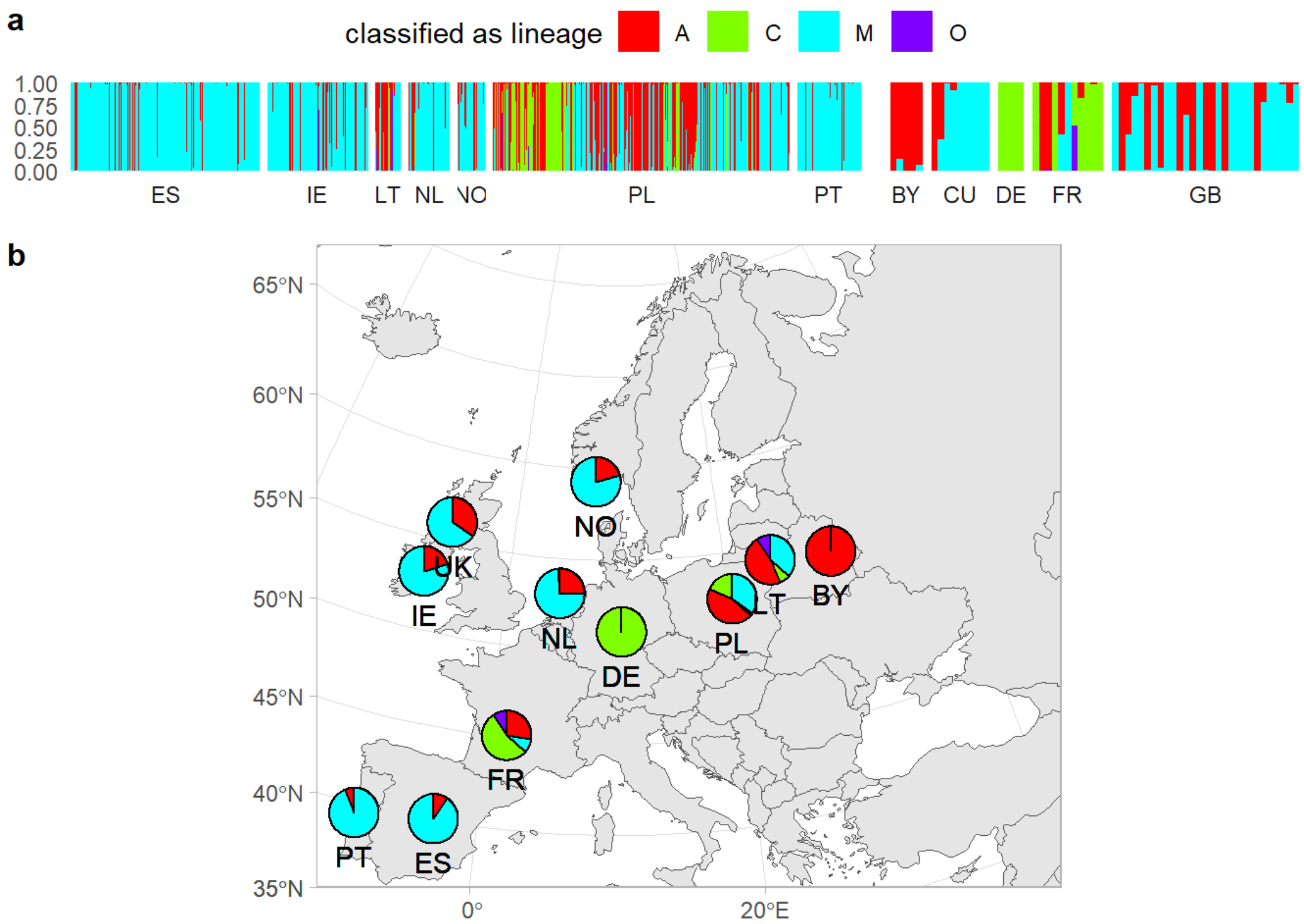

Figure 4. Classification of colonies into four evolutionary lineages, as shown in the admixture graph (a) and map (b). Country abbreviations are the same as in Table 2.

A large amount of data from both protected and unprotected populations in northeastern Poland allows us to investigate the temporal changes in the native honey bee lineage M. At the beginning of the 21st century, the mean Mahalanobis distance to lineage M increased markedly in northeastern Poland (ANOVA; years: $F_{1,896} = 652.87$, $p < 10^{-15}$; population: $F_{1,896} = 41.51$, $p = 10^{-10}$; interaction: $F_{1,896} = 197.69$, $p < 10^{-15}$). This increase was significant in both protected and unprotected populations, but particularly strong in the unprotected population (Fig. 5).

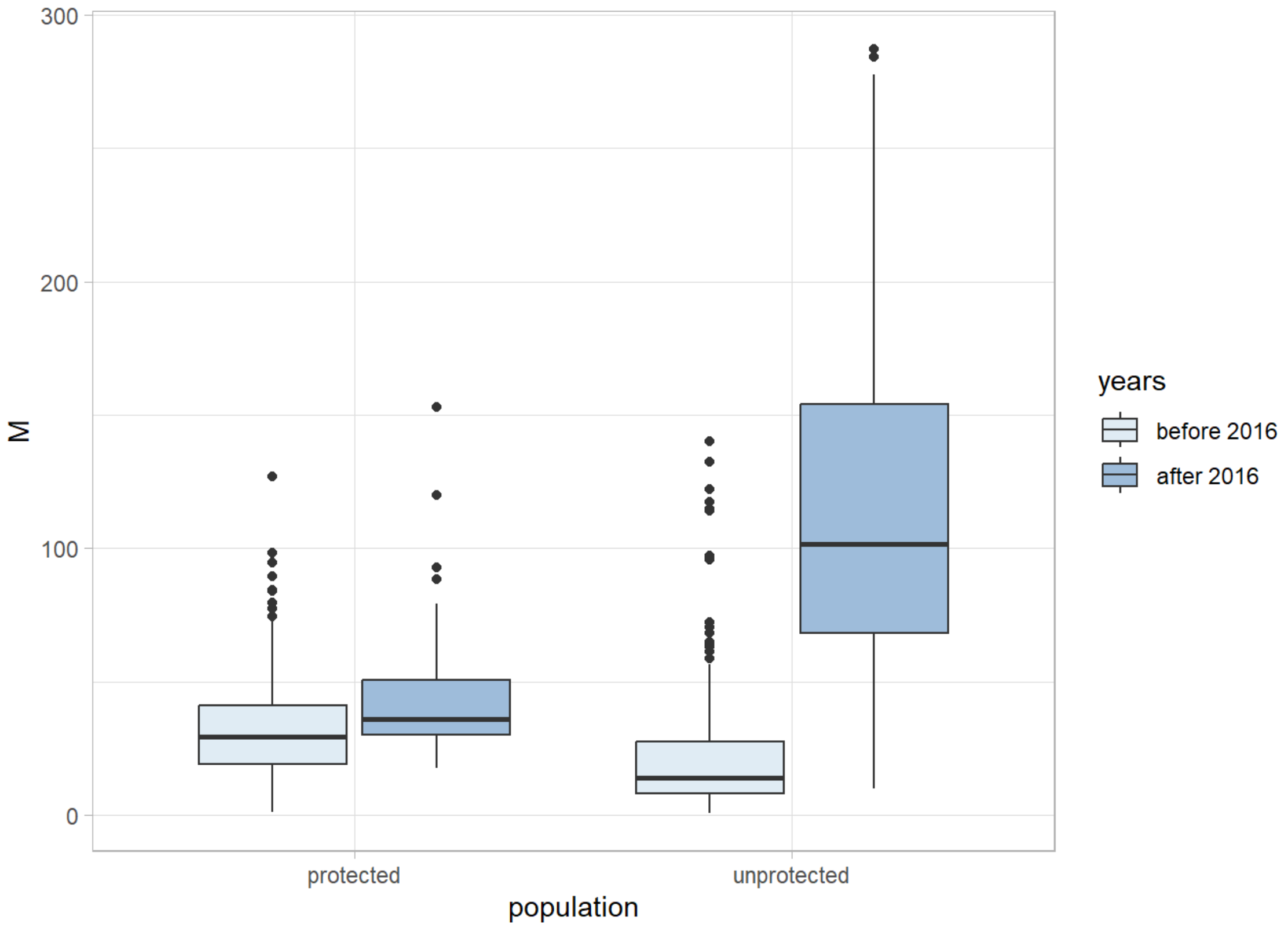

Figure 5. Mahalanobis distance of honey bee colonies to lineage M before and after 2016 in north-eastern Poland.

Discussion

The data presented here show that the proportion of lineage M in northwestern Europe differed markedly between countries (Fig. 4). In Portugal and Spain, more than 90% of colonies are of the native lineage. Samples in this region were collected from unprotected populations, showing that beekeepers use native honey bees. This is the optimal solution for protecting honey bee diversity. When natural nesting sites for honey bee colonies become scarce, beekeepers provide hives for the bees without importing non-native bees. In this region, queen breeders do not use artificial insemination [73]. This allows them to maintain a large population of honeybees that are adapted to the local environment. At the same time, the beekeeping industry in this region is thriving, and honey production is the highest in Europe [74]. A high level of similarity to lineage M is also present in the unprotected population of Ireland [48–51,75]. However, introgression from non-native lineages is larger in this country than in the Iberian Peninsula. In the Netherlands (Texel Island), and the UK (Colonsay Island), and partly Norway protected populations were sampled. In these populations, lineage M is well represented; however, in unprotected populations in these countries, lineage M is most likely much less prevalent [39]. In Poland and Lithuania, where both protected and unprotected populations were sampled, the proportion of lineage M is relatively low, but still present. In Lithuania, a relatively high proportion of colonies were classified as lineage O, which confirms earlier studies [76].

In the unprotected population of France that was sampled, only one of eleven colonies was classified as lineage M; lineage C dominates in this country, though all other lineages are present. An earlier molecular survey in France found a higher proportion of lineage M in both protected and unprotected populations [55]. However, another study revealed a relatively low percentage of lineage M in unprotected populations [77]. None of the colonies investigated in unprotected populations of Belarus were classified as lineage M, although admixture of lineage M was detected in some colonies (Fig. 5a). All five of the colonies investigated in Belarus were classified as lineage A, which occurs naturally in Africa. The mitochondrial DNA characteristic of lineage A is naturally present in the Iberian Peninsula [43], but not in northeastern Europe. A recent mitochondrial DNA survey in Central Europe detected African-ancestry haplotypes at a much lower frequency than reported here [38,41,78]. The high proportion of samples assigned to lineage A may be related to the fact that hybrids between lineages are more likely to be classified as lineage A [69]. Those hybrids exhibit an intermediate phenotype similar to the mean shape of all lineages [79].

In Germany, all of the investigated colonies belonged to lineage C (Fig. 4). These bees originated from free-living colonies in a national park [64]. The process of replacing *A. m. mellifera* with *A. m. carnica* intensified in Germany in the 1940s [80]. By the end of the 20th century, this process had advanced markedly, with most colonies representing hybrids between the two subspecies [80]. Our data suggest that, in some parts of Germany, the replacement of the native subspecies by lineage C is now complete (Fig. 5a). A similar situation occurs in the Czech Republic, where all but a few colonies were classified as *A. m. carnica* [37,38]. The data presented here indicate that *A. m. mellifera* is critically endangered in most of its native range. Surprisingly, lineage M is better preserved in Cuba, where *Apis mellifera* is not native, than in most of its native range in northwestern Europe [81,82].

There is significant geographic variability in the wing shape of honeybees in northwestern Europe. Within lineage M, two European subspecies were declared: *A. m. iberiensis* and *A. m. mellifera* [17]. The Pyrenees Mountains, a natural barrier to gene flow, lie between the two subspecies. Our data confirm the distinction between these two subspecies. Interestingly, mainly *A. m. mellifera* suffers from introgression with non-native bees. Our data show significant differences within *A. m. mellifera* between countries. Similar variation within this subspecies was detected using molecular markers [29]. This diversity may be related to natural variation shaped by natural selection [17] as well as beekeeping practices, including the introduction of commercial honeybees through breeding programs, importing queens, and transhumance movements [12]. Understanding which part of this variation is natural or human-induced is important for the adoption of optimal conservation strategies. Given the very large distribution area of *A. m. mellifera* and the various climatic conditions within it, it is highly probable that numerous ecotypes adapted to the local environment were present [28]. Some of these ecotypes, such as those occurring naturally in Germany and Czech Republic, could be irreversibly lost. The best conservation strategy is probably to protect local bees, even if they differ from the historic populations of a particular area. According to this strategy, it is better to avoid the long-distance reintroduction of *A. m. mellifera*, as they most likely represent different ecotypes. Conservation of local *A. m. mellifera* ecotypes can be achieved by designating large areas where the introduction of non-local bees is prohibited [53].

It is essential to describe the current variation and monitor how it changes. Originally, morphometry was used to describe the geographic variation of honeybees and differentiate subspecies. Later, molecular markers were introduced, improving subspecies discrimination. However, obtaining reference samples for molecular studies can be problematic in some parts of Europe [77] because populations are admixed, making it unclear which aspects of geographic variation are natural and which are related to beekeeping activities. In these cases, morphometric data can be used for cross-validation. Unlike molecular markers, morphometry is accessible to beekeepers; therefore, it can serve as a quick, inexpensive method for monitoring endangered populations. An important source of

reference data is the Morphometric Bee Data Bank in Oberursel, Germany. Unfortunately, the currently available lineage data is limited in size [62], and further studies are needed to expand upon it. The data provided here can be used for future comparisons.

We used data from northeastern Poland to analyze changes in similarity to lineage M in recent times. This region was the last refuge of *A. m. mellifera* in Poland. The results are alarming, as the similarity to lineage M decreased markedly in the unprotected population (Fig. 5). The situation is much better in the protected population, which indicates that conservation programs are essential for the protection of the remaining *A. m. mellifera* populations. The rapid changes in recent years demonstrate the urgent need for action to prevent further erosion of honey bee genetic diversity in northwestern Europe. As has been shown the introduction of maladapted bees into new areas causes increased mortality rates and contributes to bee losses [4]. Carrying poorly genes to new areas and spreading those, is even supported within the current European Union Common Agricultural Policy giving funding to “rationalising transhumance” (Regulation (EU) 2021/2115, Article 55(1):) A much more responsible action would enforce a moratorium on long distance bee movement between honey bees of different subspecies to minimize further loss of genetic diversity and avoid colony losses.

List of Abbreviations

BY: Belarus, CU: Cuba, DE: Germany, ES: Spain, FR: France, UK: the United Kingdom, IE: Ireland, LT: Lithuania, NL: the Netherlands, NO: Norway, PL: Poland, PT: Portugal.

Data Availability

The complete dataset, including wing images, landmark coordinates, geographic coordinates of sampling locations and other data, is available on the Zenodo website: https://zenodo.org/records/18845767. The R script containing all the details of the statistical analysis is available on the GitHub website: https://github.com/DrawWing/Apis-mellifera-NW-Europe.

Use of AI Writing Tools

DeepL software was used to correct English language. The AI tool was employed solely for language refinement and did not influence the empirical data, analyses, or conclusions of the study.

Author Contributions

Julita Machlowska (Writing—review & editing, Writing—original draft, Data Curation), Dariusz Gerula (Writing—review & editing, Resources, Data Curation), Andrzej Oleksa (Writing—review & editing, Resources, Data Curation), Henk Kok (Writing—review & editing, Resources, Data Curation), Grace P. McCormack (Writing—review & editing, Resources, Data Curation), Alexandra Valentine (Writing—review & editing, Resources, Data Curation), Lars Kirkerud (Writing—review & editing, Resources, Data Curation), Per Kryger (Writing—review & editing, Resources, Data Curation), Laima Blažytė-Čereškienė (Writing—review & editing, Resources, Data Curation), Martin Hasselmann (Writing—review & editing, Resources, Data Curation), Teweldemedhn Gebretinsae Hailu (Writing—review & editing, Resources, Data Curation), Benjamin Rutschmann (Writing—review & editing, Resources, Data Curation), Adam Tofilski (Writing—review & editing, Writing—original draft, Conceptualization, Formal Analysis, Methodology)

Funding

This research was funded by National Science Centre, Poland, grant number: 2021/41/B/NZ9/03153.

Acknowledgements

Andrew Abrahams provided bees from his apiary on Colonsay island. Patrick L. Kohl helped in collecting samples in Germany and Spain. We thank Adam Wright and Karin Maassen (Custos Apium)

and Lucy Tournadre (Propriétaire Courcelles project) for access to samples in France and assistance with wing sampling. Jarosław Prabucki, now deceased, provided some samples from Poland.

Conflicts of Interest

The authors declare no conflicts of interest.

References

1. Machlowska J, Gerula D, Oleksa A, Kok H, McCormack G, Valentine A, et al.. Fore wings of honey bees (Apis mellifera) from northwestern Europe. *Zenodo*. Zenodo; 2026; doi: 10.5281/zenodo.18845767.

2. Calderone NW. Insect Pollinated Crops, Insect Pollinators and US Agriculture: Trend Analysis of Aggregate Data for the Period 1992–2009. *PLOS ONE*. Public Library of Science; 2012; doi: 10.1371/journal.pone.0037235.

3. Hung K-LJ, Kingston JM, Albrecht M, Holway DA, Kohn JR. The worldwide importance of honey bees as pollinators in natural habitats. *Proc R Soc B Biol Sci*. Royal Society; 2018; doi: 10.1098/rspb.2017.2140.

4. Büchler R, Costa C, Hatjina F, Andonov S, Meixner MD, Conte YL, et al.. The influence of genetic origin and its interaction with environmental effects on the survival of *Apis mellifera* L. colonies in Europe. *J Apic Res*. 2014; doi: 10.3896/IBRA.1.53.2.03.

5. Strange JP, Garnery L, Sheppard WS. Persistence of the Landes ecotype of Apis mellifera mellifera in southwest France: confirmation of a locally adaptive annual brood cycle trait. *Apidologie*. EDP Sciences; 2007; doi: 10.1051/apido:2007012.

6. Potts SG, Roberts SPM, Dean R, Marris G, Brown MA, Jones R, et al.. Declines of managed honey bees and beekeepers in Europe. *J Apic Res*. 2010; doi: 10.3896/IBRA.1.49.1.02.

7. vanEngelsdorp D, Evans JD, Saegerman C, Mullin C, Haubruge E, Nguyen BK, et al.. Colony Collapse Disorder: A Descriptive Study. *PLOS ONE*. Public Library of Science; 2009; doi: 10.1371/journal.pone.0006481.

8. Kohl PL, Rutschmann B. European wild honeybee populations are endangered. EcoEvoRxiv; 2024;

9. Panziera D, Requier F, Chantawannakul P, Pirk CWW, Blacquiere T. The diversity decline in wild and managed honey bee populations urges for an integrated conservation approach. *Front Ecol Evol*. 2022; doi: 10.3389/fevo.2022.767950.

10. Themudo GE, Rey-Iglesia A, Robles Tascón L, Bruun Jensen A, da Fonseca RR, Campos PF. Declining genetic diversity of European honeybees along the twentieth century. *Sci Rep*. 2020; doi: 10.1038/s41598-020-67370-2.

11. vanEngelsdorp D, Meixner MD. A historical review of managed honey bee populations in Europe and the United States and the factors that may affect them. *J Invertebr Pathol*. 2010; doi: 10.1016/j.jip.2009.06.011.

12. De la Rúa P, Jaffé R, Dall'Olio R, Muñoz I, Serrano J. Biodiversity, conservation and current threats to European honeybees. *Apidologie*. 2009; doi: 10.1051/apido/2009027.

13. Meixner MD, Costa C, Kryger P, Hatjina F, Bouga M, Ivanova E, et al.. Conserving diversity and vitality for honey bee breeding. *J Apic Res*. Taylor & Francis; 2010; doi: 10.3896/IBRA.1.49.1.12.

14. Moritz RFA, Härtel S, Neumann P. Global invasions of the western honeybee (*Apis mellifera*) and the consequences for biodiversity. *Écoscience*. 2005; doi: 10.2980/i1195-6860-12-3-289.1.

15. Whitfield CW, Behura SK, Berlocher SH, Clark AG, Johnston JS, Sheppard WS, et al.. Thrice Out of Africa: Ancient and Recent Expansions of the Honey Bee, *Apis mellifera*. *Science*. 2006; doi: 10.1126/science.1132772.

16. Engel MS. The taxonomy of recent and fossil honey bees (Hymenoptera : Apidae ; *Apis*). *J Hymenopt Res*. 8:165–961999;

17. Ruttner F. Biogeography and taxonomy of honeybees. Berlin: Springer;

18. Sheppard WS, Arias MC, Grech A, Meixner MD. Apis mellifera ruttneri, a new honey bee subspecies from Malta. *Apidologie*. 1997; doi: 10.1051/apido:19970505.

19. Sheppard WS, Meixner MD. *Apis mellifera pomonella* , a new honey bee subspecies from Central Asia. *Apidologie*. 2003; doi: 10.1051/apido:2003037.

20. Franck P, Garnery L, Solignac M, Cornuet J-M. Molecular confirmation of a fourth lineage in honeybees from the Near East. *Apidologie*. 2000; doi: 10.1051/apido:2000114.

21. Hailu TG, D'Alvise P, Tofilski A, Fuchs S, Greiling J, Rosenkranz P, et al.. Insights into Ethiopian honey bee diversity based on wing geomorphometric and mitochondrial DNA analyses. *Apidologie*. 2020; doi: 10.1007/s13592-020-00796-9.

22. Alburaki M, Moulin S, Legout H, Alburaki A, Garnery L. Mitochondrial structure of Eastern honeybee populations from Syria, Lebanon and Iraq. *Apidologie*. 2011; doi: 10.1007/s13592-011-0062-4.

23. Franck P, Garnery L, Loiseau A, Oldroyd BP, Hepburn HR, Solignac M, et al.. Genetic diversity of the honeybee in Africa: microsatellite and mitochondrial data. *Heredity*. 2001; doi: 10.1046/j.1365-2540.2001.00842.x.

24. Chen C, Liu Z, Pan Q, Chen X, Wang H, Guo H, et al.. Genomic analyses reveal demographic history and temperate adaptation of the newly discovered honey bee subspecies *Apis* mellifera sinisxinyuan n. ssp. *Mol Biol Evol*. 2016; doi: 10.1093/molbev/msw017.

25. Crane E. The World History of Beekeeping and Honey Hunting. Routledge;

26. Francis RM, Kryger P, Meixner M, Bouga M, Ivanova E, Andonov S, et al.. The genetic origin of honey bee colonies used in the COLOSS Genotype-Environment Interactions Experiment: a comparison of methods. *J Apic Res*. Taylor & Francis; 2014; doi: 10.3896/IBRA.1.53.2.02.

27. Ilyasov RA, Lee M, Takahashi J, Kwon HW, Nikolenko AG. A revision of subspecies structure of western honey bee Apis mellifera. *Saudi J Biol Sci*. 2020; doi: 10.1016/j.sjbs.2020.08.001.

28. Meixner MD, Pinto MA, Bouga M, Kryger P, Ivanova E, Fuchs S. Standard methods for characterising subspecies and ecotypes of *Apis mellifera*. *J Apic Res*. 2013; doi: 10.3896/IBRA.1.52.4.05.

29. Momeni J, Parejo M, Nielsen RO, Langa J, Montes I, Papoutsis L, et al.. Authoritative subspecies diagnosis tool for European honey bees based on ancestry informative SNPs. *BMC Genomics*. 2021; doi: 10.1186/s12864-021-07379-7.

30. Leroy T, Faux P, Basso B, Eynard S, Wragg D, Vignal A. Inferring Long-Term and Short-Term Determinants of Genetic Diversity in Honey Bees: Beekeeping Impact and Conservation Strategies. *Mol Biol Evol*. 2024; doi: 10.1093/molbev/msae249.

31. Soland-Reckeweg G, Heckel G, Neumann P, Fluri P, Excoffier L. Gene flow in admixed populations and implications for the conservation of the Western honeybee, Apis mellifera. *J Insect Conserv*. 2009; doi: 10.1007/s10841-008-9175-0.

32. Cale GH Jr, Gowen JW. Heterosis in the honey bee (*Apis mellifera* L.). *Genetics*. 1956; doi: 10.1093/genetics/41.2.292.

33. Fresnaye J, Lavie P, Boesiger E. La variabilité de la production du miel chez l'abeille de race noire (*Apis mellifica* L.) et chez quelques hybrides interraciaux. *Apidologie*. EDP Sciences; 5:1–201974;

34. Peer DF. Further Studies on the Mating Range of the Honey Bee, *Apis mellifera* L. *Can Entomol*. 1957; doi: 10.4039/Ent89108-3.

35. Kauhausen-Keller D, Keller R. Morphometrical control of pure race breeding in the honeybee (Apis mellifera L). *Apidologie*. 1994; doi: 10.1051/apido:19940202.

36. Maul V, Hähnle A. Morphometric studies with pure bred stock of Apis mellifera carnica Pollmann from Hessen. *Apidologie*. 1994; doi: 10.1051/apido:19940201.

37. Knoll A, Šotek M, Prouza J, Langová L, Přidal A, Urban T, et al.. Assessing Genetic Diversity and Population Structure of Western Honey Bees in the Czech Republic Using 22 Microsatellite Loci. *Insects*. publisher; 2025; doi: 10.3390/insects16010055.

38. Knoll A, Langová L, Přidal A, Urban T, Knoll A, Langová L, et al.. Haplotype Diversity in mtDNA of Honeybee in the Czech Republic Confirms Complete Replacement of Autochthonous Population with the C Lineage. *Insects*. publisher; 2024; doi: 10.3390/insects15070495.

39. Nielsdatter MG, Larsen M, Nielsen LG, Nielsen MM, Rasmussen C. History of the displacement of the European dark bee (*Apis mellifera mellifera*) in Denmark. *J Apic Res*. 2021; doi: 10.1080/00218839.2020.1826111.

40. Bieńkowska M, Splitt A, Węgrzynowicz P, Maciorowski R. The Buzz Changes within Time: Native Apis mellifera mellifera Honeybee Subspecies Less and Less Popular among Polish Beekeepers Since 1980. *Agriculture*. 2021; doi: 10.3390/agriculture11070652.

41. Oleksa A, Kusza S, Tofilski A. Mitochondrial DNA suggests the introduction of honeybees of African ancestry to east-central Europe. *Insects*. 2021; doi: 10.3390/insects12050410.

42. Requier F, Garnery L, Kohl PL, Njovu HK, Pirk CWW, Crewe RM, et al.. The conservation of native honey bees is crucial. *Trends Ecol Evol*. 2019; doi: 10.1016/j.tree.2019.04.008.

43. Cánovas F, De la Rúa P, Serrano J, Galián J. Geographical patterns of mitochondrial DNA variation in *Apis mellifera iberiensis* (Hymenoptera: Apidae). *J Zool Syst Evol Res*. 2008; doi: 10.1111/j.1439-0469.2007.00435.x.

44. Chávez-Galarza J, Henriques D, Johnston JS, Carneiro M, Rufino J, Patton JC, et al.. Revisiting the Iberian honey bee (*Apis mellifera iberiensis*) contact zone: maternal and genome-wide nuclear variations provide support for secondary contact from historical refugia. *Mol Ecol*. 2015; doi: 10.1111/mec.13223.

45. De la Rúa P, Jiménez Y, Galián J, Serrano J. Evaluation of the biodiversity of honey bee (Apis mellifera) populations from eastern Spain. *J Apic Res*. 43:162–62004;

46. Pinto MA, Henriques D, Neto M, Guedes H, Muñoz I, Azevedo JC, et al.. Maternal diversity patterns of Ibero-Atlantic populations reveal further complexity of Iberian honeybees. *Apidologie*. 2013; doi: 10.1007/s13592-013-0192-y.

47. Rutschmann B, Kohl PL, Machado A, Steffan-Dewenter I. Semi-natural habitats promote winter survival of wild-living honeybees in an agricultural landscape. *Biol Conserv*. 2022; doi: 10.1016/j.biocon.2022.109450.

48. Browne KA, Hassett J, Geary M, Moore E, Henriques D, Soland-Reckeweg G, et al.. Investigation of free-living honey bee colonies in Ireland. *J Apic Res*. Taylor & Francis; 2020; doi: 10.1080/00218839.2020.1837530.

49. Hassett J, Browne KA, McCormack GP, Moore E, Society NIHB, Soland G, et al.. A significant pure population of the dark European honey bee (*Apis mellifera mellifera*) remains in Ireland. *J Apic Res*. 2018; doi: 10.1080/00218839.2018.1433949.

50. Jensen AB, Palmer KA, Boomsma JJ, Pedersen BV. Varying degrees of *Apis mellifera ligustica* introgression in protected populations of the black honeybee, *Apis mellifera mellifera* , in northwest Europe. *Mol Ecol*. 2005; doi: 10.1111/j.1365-294X.2004.02399.x.

51. Valentine A, Moro A, Briggs E, Collier B, Sandoval K, Binetti C, et al.. Introgressive hybridisation puts the distinctive population of Apis mellifera mellifera in Ireland at risk: evidence from a multidisciplinary approach. *J Apic Res*. Taylor & Francis; 2025; doi: 10.1080/00218839.2024.2404297.

52. Henriques D, Browne KA, Barnett MW, Parejo M, Kryger P, Freeman TC, et al.. High sample throughput genotyping for estimating C-lineage introgression in the dark honeybee: an accurate and cost-effective SNP-based tool. *Sci Rep*. 2018; doi: 10.1038/s41598-018-26932-1.

53. Pinto MA, Henriques D, Chávez-Galarza J, Kryger P, Garnery L, Zee R van der, et al.. Genetic integrity of the Dark European honey bee (*Apis mellifera mellifera*) from protected populations: a genome-wide assessment using SNPs and mtDNA sequence data. *J Apic Res*. 2014; doi: 10.3896/IBRA.1.53.2.08.

54. Oleksa A, Chybicki I, Tofilski A, Burczyk J. Nuclear and mitochondrial patterns of introgression into native dark bees (*Apis mellifera mellifera*) in Poland. *J Apic Res*. 2011; doi: 10.3896/IBRA.1.50.2.03.

55. Bertrand B, Alburaki M, Legout H, Moulin S, Mougel F, Garnery L. MtDNA COI-COII marker and drone congregation area: An efficient method to establish and monitor honeybee (*Apis mellifera* L.) conservation centres. *Mol Ecol Resour*. 2015; doi: 10.1111/1755-0998.12339.

56. Parejo M, Henriques D, Pinto MA, Soland-Reckeweg G, Neuditschko M. Empirical comparison of microsatellite and SNP markers to estimate introgression in *Apis mellifera mellifera*. *J Apic Res*. 2018; doi: 10.1080/00218839.2018.1494894.

57. Muñoz I, Henriques D, Johnston JS, Chávez-Galarza J, Kryger P, Pinto MA. Reduced SNP Panels for Genetic Identification and Introgression Analysis in the Dark Honey Bee (Apis mellifera mellifera). Blenau W, editor. *PLOS ONE*. 2015; doi: 10.1371/journal.pone.0124365.

58. García CAY, Rodrigues PJ, Tofilski A, Elen D, McCormak GP, Oleksa A, et al.. Using the Software DeepWings© to Classify Honey Bees across Europe through Wing Geometric Morphometrics. *Insects*. 2022; doi: 10.3390/insects13121132.

59. Muñoz I, Henriques D, Jara L, Johnston JS, Chávez-Galarza J, De La Rúa P, et al.. SNPs selected by information content outperform randomly selected microsatellite loci for delineating genetic identification and introgression in the endangered dark European honeybee (*Apis mellifera mellifera*). *Mol Ecol Resour*. 2017; doi: 10.1111/1755-0998.12637.

60. Chen C, Parejo M, Momeni J, Langa J, Nielsen RO, Shi W, et al.. Population Structure and Diversity in European Honey Bees (*Apis mellifera* L.) - An Empirical Comparison of Pool and Individual Whole-Genome Sequencing. *Genes*. Multidisciplinary Digital Publishing Institute; 2022; doi: 10.3390/genes13020182.

61. Kandemir İ, Özkan A, Fuchs S. Reevaluation of honeybee (*Apis mellifera*) microtaxonomy: a geometric morphometric approach. *Apidologie*. 2011; doi: 10.1007/s13592-011-0063-3.

62. Nawrocka A, Kandemir İ, Fuchs S, Tofilski A. Computer software for identification of honey bee subspecies and evolutionary lineages. *Apidologie*. 2017; doi: 10.1007/s13592-017-0538-y.

63. Oleksa A, Căuia E, Siceanu A, Puškadija Z, Kovačić M, Pinto MA, et al.. Collection of wing images for conservation of honey bees (*Apis mellifera*) biodiversity in Europe. *Zenodo*. 2022; doi: 10.5281/zenodo.7244070.

64. Kohl PL, Rutschmann B. The neglected bee trees: European beech forests as a home for feral honey bee colonies. *PeerJ*. 2018; doi: 10.7717/peerj.4602.

65. Oleksa A, Gawroński R, Tofilski A. Rural avenues as a refuge for feral honey bee population. *J Insect Conserv*. 2013; doi: 10.1007/s10841-012-9528-6.

66. Tofilski A. Using geometric morphometrics and standard morphometry to discriminate three honeybee subspecies. *Apidologie*. 2008; doi: 10.1051/apido:2008037.

67. Masaquiza D, Ferrán MO, Guamán S, Naranjo E, Vaca M, Curbelo LM, et al.. Geometric Morphometric Analysis of Wing Shape to Identify Populations of Apis mellifera in Camagüey, Cuba. *Insects*. 2023; doi: 10.3390/insects14030306.

68. Masaquiza D, Ferran MO, Arenal CA. Collection of images of honey bee wings (Apis mellifera) from Cuba. Zenodo; 2023; doi: 10.5281/zenodo.7662013.

69. Oleksa A, Căuia E, Siceanu A, Puškadija Z, Kovačić M, Pinto MA, et al.. Honey bee (*Apis mellifera*) wing images: a tool for identification and conservation. *GigaScience*. 2023; doi: 10.1093/gigascience/giad019.

70. Baken EK, Collyer ML, Kaliontzopoulou A, Adams DC. geomorph v4.0 and gmShiny: Enhanced analytics and a new graphical interface for a comprehensive morphometric experience. *Methods Ecol Evol*. 2021; doi: 10.1111/2041-210X.13723.

71. Schlager S. Morpho and Rvcg – Shape Analysis in R. *Stat Shape Deform Anal*. Elsevier;

72. Kaur H, Ganie SA, Tofilski A. Morphometric identification of an unknown honey bee colony: an example from north India. *J Apic Sci*. 2024; doi: 10.2478/JAS-2024-0013.

73. Lodesani M, Costa C. Bee breeding and genetics in Europe. *Bee World*. 84:69–852003;

74. Jones R. European beekeeping in the 21st century: strengths, weaknesses, opportunities, threats. *Bee World*. 2004; doi: 10.1080/0005772X.2004.11099637.

75. Garcia CAY, Henriques D, Cilia G, Rufino J, Vella C, Aglagane A, et al.. Contrasting whole-genome diversity patterns and adaptation in honeybees from two southern European glacial refugia. *iScience*. Elsevier; 2026; doi: 10.1016/j.isci.2025.114497.

76. Ceksteryte V, Paplauskiene V, Tamasauskiene D, Pasakinskiene I, Mazeikiene I. Genetic characterization of Lithuanian honeybee lines based on ISSR polymorphism. *Apidologie*. 2012; doi: 10.1007/s13592-012-0140-2.

77. Wragg D, Marti-Marimon M, Basso B, Bidanel J-P, Labarthe E, Bouchez O, et al.. Whole-genome resequencing of honeybee drones to detect genomic selection in a population managed for royal jelly. *Sci Rep*. Nature Publishing Group; 2016; doi: 10.1038/srep27168.

78. Orlovskytė S, Budrys E, Skrodenytė-Arbačiauskienė V, Blažytė-Čereškienė L. The dark European honey bee Apis mellifera mellifera in Lithuania: data on mitotype diversity of native bee population. *J Apic Res*. Taylor & Francis; 2025; doi: 10.1080/00218839.2024.2327125.

79. Węgrzynowicz P, Gerula D, Tofilski A, Panasiuk B, Bieńkowska M. Maternal Inheritance in Hybrids of Three Honey Bee Subspecies. *J Apic Sci*. 2019; doi: 10.2478/jas-2019-0010.

80. Moritz RFA. The limitations of biometric control on pure race breeding in Apis mellifera. *J Apic Res*. 1991; doi: 10.1080/00218839.1991.11101234.

81. García CAY, Luis AR, Pérez Piñeiro A, Pérez Morfi A, Invernizzi C, Tomasco IH. Cuban honey bees: significant differentiation from European honey bees in incomplete isolation. *J Apic Res*. Taylor & Francis; 2021; doi: 10.1080/00218839.2020.1841460.

82. Masaquiza D, Ferrán MO, Guamán S, Naranjo E, Vaca M, Curbelo LM, et al.. Geometric morphometric analysis of wing shape to identify populations of *Apis mellifera* in Camagüey, Cuba. *Insects*. 2023; doi: 10.3390/insects14030306.
