# Supplementary material for: Country specific hybridization of honey bees from lineage M
Source: Gigascience. 2026 Sep 15;15:giag093. doi: 10.1093/gigascience/giag093 (PMC13615543; doi:10.1093/gigascience/giag093)
Supplement: Supplementary file 3 [file giag093_giga-d-26-00106_revision_1.pdf]

GigaScience

Country specific hybridization of honey bees from lineage M

--Manuscript Draft--

**Manuscript Number:**	GIGA-D-26-00106R1
**Full Title:**	Country specific hybridization of honey bees from lineage M
**Article Type:**	Data Note
**Funding Information:**	Narodowe Centrum Nauki (2021/41/B/NZ9/03153) — Prof Adam Tofilski
**Abstract:**	Background<br><br>Honey bees are essential pollinators supporting agricultural production and wild plant diversity. In evolutionary lineage M, some populations are threatened by genetic erosion caused by the widespread introduction of commercially bred queens. To assess this risk, wing images from existing and new datasets were used to assign them to four evolutionary lineages (A, C, M, and O). The new dataset consisted of 29,043 wing images representing 1,342 colony samples from ten countries.<br><br>Results<br><br>Overall, 63.7% of colonies belonged to lineage M, whereas 27.5% were classified as A, 7.9% as C, and 0.8% as O. Lineage M remains prevalent in unprotected populations in Portugal, Spain, and Ireland, as well as in protected populations elsewhere. In contrast, a pronounced decline was observed in unprotected populations in northeastern Poland.<br><br>Conclusions<br><br>These findings reveal strong regional differences in the persistence of lineage M and underscore the need for coordinated conservation efforts throughout Europe. The data provided in this study should allow for more accurate discrimination between native and introduced phenotypes.
**Corresponding Author:**	Adam Tofilski<br>University of Agriculture in Krakow: Uniwersytet Rolniczy im Hugona Kollataja w Krakowie<br>Krakow, POLAND
**Corresponding Author Secondary Information:**	
**Corresponding Author's Institution:**	University of Agriculture in Krakow: Uniwersytet Rolniczy im Hugona Kollataja w Krakowie
**Corresponding Author's Secondary Institution:**	
**First Author:**	Julita Machlowska
**First Author Secondary Information:**	
**Order of Authors:**	Julita Machlowska
	Dariusz Gerula
	Andrzej Oleksa
	Henk Kok
	Grace Patricia McCormack
	Alexandra Valentine
	Lars Kirkerud
	Per Kryger
	Laima Blažytė-Čereškienė

Powered by Editorial Manager® and ProduXion Manager® from Aries Systems Corporation

	Martin Hasselmann
	Teweldemedhn Gebretinsae Hailu
	Benjamin Rutschmann
	Adam Tofilski
**Order of Authors Secondary Information:**	
**Response to Reviewers:**	July 22, 2026<br>Dear Editor,<br>We are grateful to the reviewers for their constructive comments and corrections. We have revised the manuscript according to most of their suggestions. We believe these changes have substantially improved the manuscript.<br>Please see our point-by-point responses to the reviewers below. We have indicated these responses with "Response:"<br><br>Editor comments:<br>In addition, please register any new software application in the bio.tools and SciCrunch.org databases to receive RRID (Research Resource Identification Initiative ID) and biotoolsID identifiers, and include these in your manuscript. Computational workflows should be registered in workflowhub.eu and the DOIs cited in the relevant places in the manuscript. These will facilitate tracking, reproducibility and re-use of your tool.<br>Response: We added section “Availability of Source Code and Requirements” (lines: 376-384) with information about this project, including: bio.tools and WorkflowHub. Our earlier attempt to obtain RRID from scicrunch.org was rejected because our project is not “biomedical” and is not related to “antibodies, organisms, chemicals, plasmids or cell lines”<br><br>Reviewer #1:<br>The manuscript GIGA-D-26-00106, entitled “Country-specific hybridization of honey bees from lineage M,” analyzes Apis mellifera samples from 12 countries and applies geometric morphometry (GM) of the wing to classify honey bees for discrimination purposes.<br>This manuscript addresses an important and timely topic in bee discrimination. However, several issues should be considered before a final decision is made.<br>Response: We appreciate the positive feedback.<br><br>The use of geometric morphometry (GM) for lineage discrimination (A, M, etc.) is appropriate and well established. However, in the absence of genomic validation on the same specimens, the assignment of individuals to evolutionary lineages remains indirect. Reliance on previously published classifications introduces uncertainty, particularly in the context of hybridization. I recommend that the authors either provide genetic validation for the analyzed samples or explicitly discuss this limitation and its potential impact on classification accuracy.<br>Response: We agree that genetic material would be an important part of the dataset; however, most of the wing images were collected many years ago and it is now impossible to obtain genetic material. We used morphometry for identification of evolutionary lineages. This is justified because honey bee subspecies and lineages were described using morphometric measurements rather than genetic markers. Despite the fact that the original description of honey bee subspecies was morphometric, most data which are now considered as a reference for genetic markers were not validated using morphometric methods. We believe that the material we provide can be used to improve cross-validation between morphometric and molecular studies. We mention the problem of molecular validation in lines 351-357.<br>We admit that genetic markers can be more effective in detection of hybrids and mention this in lines 308-310.<br><br>2. Figure 5. How can the sampling time categories (“before 2016” and “after 2016”) be interpreted? How can temporal changes in protected and unprotected samples be evaluated from this figure?<br>Response: We explained the categories in figure 5. We replaced “years” with “samples collected”. Additionally, we explained figure 5 in lines 270-272.

3. The observed hybridization patterns may be influenced not only by classification outcomes but also by spatial structure, landscape connectivity, and beekeeping practices. Natural dispersal via swarming is typically limited in range, whereas long-distance gene flow is often human-mediated. It would be important to discuss potential geographic or ecological barriers to dispersal, as well as management practices (e.g., migratory beekeeping) that may affect hybridization. Without considering these factors, the classification remains largely descriptive.
Response: We agree that the observed geographic variation of honey bees in Europe can be shaped by both natural and human-induced processes. We explain this in lines 324–330.

4. The conclusion is unclear. PCA indicates substantial overlap between groups, whereas the same data appear to show separation under a supervised model. This discrepancy requires clarification. Please explain how the supervised model achieves separation despite the overlap observed in PCA, and provide details on model validation (e.g., cross-validation, accuracy, or overfitting control) to support its reliability.
Response: We explained the procedure for analyzing wing shape variation in lines 208-218. We added results of classification with cross-validation in lines 235-239.

5. The Methods section lacks critical detail. It is not clear which wing was analyzed (right, left, or both). Additionally, the rationale for selecting the 12 countries is not provided. What is the history and intensity of beekeeping in these regions, and how might this influence the results?
Response: Measurements were taken from both the left and right forewings. If the side was known, it was indicated in the file name with the letters L and R for left and right wings, respectively. The differences between the left and right wings are relatively small, and their influence on geographic variation is negligible (Janczyk & Tofilski 2021). We explain this in lines 180-183.
We have attempted to collect as much material from lineage M as possible. The main factor affecting geographic variation of honey bees is intensity of queen replacement. Other beekeeping practices are less important. We provide information if the samples originated from protected or unprotected populations.

6. There is also a substantial imbalance in sample sizes (e.g., ~900 samples from Poland versus ~5 from Belarus). This raises concerns regarding statistical robustness, potential bias in classification performance, and the reliability of conclusions for underrepresented groups. Please clarify how these issues were addressed and discuss the associated limitations.
Response: We agree that the sample sizes differ markedly between countries. We would like to analyze more samples, however, they are not available for us. We hope the dataset will be enlarged in future. We mentioned this problem in lines 359-361.

7. Furthermore, although a COLOSS standard morphometric protocol is available (Meixner et al., 2012), it is not referenced or discussed. The numbering and ordering of landmarks also appear to differ from this standard. Please clarify which protocol was followed and justify any deviations.
Response: Unfortunately, the configuration of landmarks used for geometric morphometrics of honey bees differs between studies. Meixner et al. (2013) explained this problem well. The website referenced in the figure caption (http://apiclass.mnhn.fr/) is no longer accessible, and the landmark configuration depicted in the figure was employed in only three publications, all of which were co-authored by Michel Baylac. The last of these publications was published ten years ago. Currently, there is no generally accepted landmark configuration. We used a landmark configuration consistent with earlier studies related to lineage classification and available data from earlier studies. We explain this in lines 186-189. Fortunately, the landmarks can be reordered relatively easily. Furthermore, we provide all wing images, which can be remeasured if necessary.

8. Line 328: Why do the authors conclude that maladapted bees introduced into new areas carry “poor” genes? This statement appears speculative and requires clarification and supporting evidence.
Response: We removed the fragment related to “poor genes.” We explained in lines 368-369 that the bees introduced by beekeepers are “maladapted to the local

	environment."<br><br>Overall, I recommend major revisions, as several key methodological and interpretative issues need to be addressed to support the conclusions.<br>Response: Thank you for the recommendation, as well as the constructive comments and corrections.<br><br>Reviewer #2:<br>This is an important paper, as it provides evidence to counter the argument that the M lineage of honey bees no longer exists in many European locations.<br><br>The data reported in the paper are not a randomised survey, but combine existing published material and new previously unpublished data, but with a patchy coverage, although Poland is comprehensively covered. The paper reports on some 29,000 images of wings which represent over 1,000 colonies from twelve countries. The images are now placed in a public repository, a valuable resource, as there are limited numbers of reference samples from many countries.<br><br>The results show that M Lineage is still prevalent in general bee populations in Ireland, Portugal, and Spain, but only exists in populations under some kind of protection in most other countries of its former range.<br>Response: We appreciate the positive feedback.<br><br>The paper explains that both molecular and morphometric techniques can be used to discriminate between populations, but notes that molecular techniques are not usually available to beekeepers, for whom morphometric techniques are more appropriate. There could perhaps be a comment added about divergence between the two techniques. What happens in beekeepers select populations on the basis of just a few wing morphometric characters? Maybe comment on this…<br>Response: We agree that selecting for a small number of traits characteristic of native bees is problematic. We explain this in lines 336-350. We point out that this problem is not limited to morphometry, but also applies to a small number of genetic markers. Therefore, we suggest that the best conservation strategy for honey bees is to designate protected areas to which non-native bees cannot be imported.<br><br>Sadly in many parts of its former area, such as Germany and the Czech Republic, A.m.m. may be effectively extinct, but the paper makes a plea for local beekeepers to work with locally adapted bees rather than trying to reintroduce A.m.m. from elsewhere, where the ecotypes are probably different from the extinct former populations. This is an important point.<br>Response: Thank you for the positive response and constructive comments.
**Additional Information:**	
**Question**	**Response**
Are you submitting this manuscript to a special series or article collection?	No
**Experimental design and statistics**<br><br>Full details of the experimental design and statistical methods used should be given in the Methods section, as detailed in our Minimum Standards Reporting Checklist. Information essential to interpreting the data presented should be made available in the figure legends.<br><br>Have you included all the information	Yes

requested in your manuscript?	
**Resources**<br><br>A description of all resources used, including antibodies, cell lines, animals and software tools, with enough information to allow them to be uniquely identified, should be included in the Methods section. Authors are strongly encouraged to cite Research Resource Identifiers (RRIDs) for antibodies, model organisms and tools, where possible.<br><br>Have you included the information requested as detailed in our Minimum Standards Reporting Checklist?	Yes
**Availability of data and materials**<br><br>All datasets and code on which the conclusions of the paper rely must be either included in your submission or deposited in publicly available repositories (where available and ethically appropriate), referencing such data using a unique identifier in the references and in the "Availability of Data and Materials" section of your manuscript.<br><br>Have you have met the above requirement as detailed in our Minimum Standards Reporting Checklist?	Yes
GigaScience has policies and guidelines in place for the use of generative AI-writing tools such as ChatGPT. If you have used such writing tools to assist with writing the manuscript this must be declared and cited in the text. Authors should not list AI-writing tools and other AI-assisted technologies as an author or co-author and should acknowledge that they are fully responsible for text generated or refined by AI-writing tools.<p><br><br>A summary of use (particularly in the introduction or among methods) needs to be included at the end of the paper, and	Yes

the outputs should also be included as a supplementary file hosted in GigaDB or other open repositories. Please read our guidelines for more information. <p><br><br>By submitting to GigaScience, you are aware of the journal's AI-writing tools policy, and if you have declared use of such tools below, you have acknowledged this where appropriate in your manuscript and have made a summary of use and outputs available. <p><br><b>AI-assisted writing tools have been used in the preparation of this manuscript?	

Manuscript

Click here to access/download;Manuscript;Apis-mellifera-NW-Europe-GigaScience-revision2 (1).docx

Country specific hybridization of honey bees from lineage M

Julita Machlowska[1], Dariusz Gerula[2], Andrzej Oleksa[3], Henk Kok[4], Grace P. McCormack[5], Alexandra Valentine[5], Lars Kirkerud[6],Per Kryger[7], Laima Blažytė-Čereškienė[8], Martin Hasselmann[9],, Teweldemedhn Gebretinsae Hailu[10], Benjamin Rutschmann[11,12], Adam Tofilski[1,*]

1. Department of Zoology and Animal Welfare, University of Agriculture in Krakow, 29 Listopada 56, 31-425 Kraków, Poland, julita.machlowska@gmail.com, rotofils@cyf-kr.edu.pl
2. Apiculture Division, National Institute of Horticultural Research, 24-100 Pulawy, Poland, dariusz.gerula@inhort.pl
3. Department of Genetics, Kazimierz Wielki University, Powstańców Wielkopolskich 10, 85-090 Bydgoszcz, Poland, olek@ukw.edu.pl
4. Nederlandse Bijenhoudersvereniging, Stationsweg 94A, 6711 PW, Ede, Netherlands, h.s.kok.veenendaal@gmail.com
5. Molecular Evolution and Systematics Laboratory, University of Galway, University Rd., H91 TK33 Galway, Ireland, grace.mccormack@universityofgalway.ie, a.valentine2@universityofgalway.ie
6. Norsk Brunbielag, Dampsagveien 14, 2004 Lillestrøm, Norway, lkirkerd@online.no
7. Department of Agroecology - Agricultural biodiversity, Aarhus University, Forsøgsvej 1, 4200 Slagelse, Denmark, per.kryger@agro.au.dk
8. Nature Research Centre, Akademijos St. 2, 08412 Vilnius, Lithuania, laima.blazyte@gamtc.lt
9. Department of Population Genomics, Institute of Animal Science, University of Hohenheim, Garbenstr. 17, 70599 Stuttgart, Germany, martin.hasselmann@uni-hohenheim.de
10. Department of Animal Breeding and Husbandry in the Tropics and Subtropics, University of Hohenheim, Garbenstr. 17, 70599 Stuttgart, Germany, teweldemedhng.hailu@uni-hohenheim.de
11. Agroecology and Environment, Agroscope, Reckenholzstrasse 191, 8046 Zurich, Switzerland
12. Department of Animal Ecology and Tropical Biology, Biocenter, University of Würzburg, Würzburg, Germany, benjamin.rutschmann@uni-wuerzburg.de

Corresponding author: Adam Tofilski, Department of Zoology and Animal Welfare, University of Agriculture in Krakow, 29 Listopada 56, 31-425 Kraków, Poland, rotofils@cyf-kr.edu.pl

ORCIDs:

Julita Machlowska[1] orcid: 0000-0001-9951-9248

Dariusz Gerula[2] orcid: 0000-0003-3875-2657

Andrzej Oleksa[3] orcid: 0000-0002-0414-8075

Henk Kok[4]

Grace P. McCormack[5] orcid: 0000-0003-4960-5185

Alexandra Valentine[5] orcid: 0009-0002-4748-3735

Lars Kirkerud[6]

Per Kryger[7] orcid: 0000-0001-5306-3230

Laima Blažytė-Čereškienė[8] orcid: 0000-0002-0801-0196

Martin Hasselmann[9] orcid: 0000-0003-3305-4024

Teweldemedhn Gebretinsae Hailu[10] orcid: 0000-0002-3043-995X
Benjamin Rutschmann[11,12] orcid: 0000-0001-6589-6408
Adam Tofilski[1] orcid: 0000-0002-3898-7029

Abstract

Background

Honey bees are essential pollinators supporting agricultural production and wild plant diversity. In evolutionary lineage M, some populations are threatened by genetic erosion caused by the widespread introduction of commercially bred queens. To assess this risk, wing images from existing and new datasets were used to assign them to four evolutionary lineages (A, C, M, and O). The new dataset consisted of 29,043 wing images representing 1,342 colony samples from ten countries.

Results

Overall, 63.7% of colonies belonged to lineage M, whereas 27.5% were classified as A, 7.9% as C, and 0.8% as O. Lineage M remains prevalent in unprotected populations in Portugal, Spain, and Ireland, as well as in protected populations elsewhere. In contrast, a pronounced decline was observed in unprotected populations in northeastern Poland.

Conclusions

These findings reveal strong regional differences in the persistence of lineage M and underscore the need for coordinated conservation efforts throughout Europe. The data provided in this study should allow for more accurate discrimination between native and introduced phenotypes.

Key words: Apis mellifera, wing, geometric morphometrics, M-lineage bees, northwestern Europe

Introduction

Honey bees (*Apis mellifera*, NCBI:txid7460) are valuable pollinators that contribute significantly to agricultural productivity and the maintenance of wild plant diversity. In agriculture, their pollination services are essential for many fruit, vegetable, and nut crops. The global annual economic value directly attributable to animal pollination is estimated to be in the hundreds of billions of U.S. dollars [1]. Beyond agriculture, honey bees contribute to the pollination of a vast array of wild plants, supporting ecosystem function and the reproduction of non-crop flora, which is essential for broader biodiversity [2]. The resilience of these pollination services is crucially linked to genetic diversity within honey bee populations. Subspecies and ecotypes adapted to specific climatic and floral conditions often have superior survival rates in their native ranges compared to non-adapted, commercially imported stocks [3,4]. Managed honey bee populations in the USA and parts of Europe have experienced significant declines, often categorized as colony collapse disorder [5,6], and evidence also indicates declines in wild-living honey bee populations across much of Europe [7]. Apart from primary drivers such as pathogens and agrochemicals, the loss of population genetic variability and the erosion of adaptation to the local environment have been suggested as causes of the decline [8–10]. Therefore, preserving the intraspecific biodiversity of honey bees is a conservation imperative and a practical necessity for sustaining robust pollination services and agricultural security in the face of environmental change [11,12].

The natural distribution of *Apis mellifera* includes Africa, Europe, and Western Asia; however, this species is found worldwide due to multiple introductions [13,14]. Approximately 30 honey bee subspecies have been identified [15–18]. Four major evolutionary lineages are recognized within the species: the African lineage (A), the Central and Eastern European lineage (C), the Western and Northern European lineage (M), and the Middle Eastern and Central Asian lineage (O) [16,19]. Recently, honey bees from Ethiopia, originally classified as lineage A, were reclassified as lineage Y using molecular and wing morphometric analyses [20]. Another lineage, lineage Z, was identified in Syria and Lebanon [21,22].

Lineage M's natural distribution extends from Ireland and Portugal in the west to the Ural Mountains in Russia in the east [16]. Recently, it was suggested that lineage M also occurs naturally in

western China [23]. The northernmost limit of the natural distribution of *A. mellifera* and its M lineage is southern Scandinavia, around 60° N [24]. The southernmost limits of lineage M extend along the Mediterranean coast of Spain and France (including Corsica) and the northern slopes of the Alps. Further east, there was a hybrid zone between lineages M and C, which was less clearly defined. It was assumed that lineage M occurred in northern Austria, the Czech Republic, most of Poland, and the northern part of Ukraine [16,25]. Within this distribution, three subspecies were distinguished: *A. mellifera iberiensis* in the Iberian Peninsula; *A. mellifera mellifera* in the rest of the European distribution of lineage M; and *A. mellifera sinisxinyuan* in western China [23,26,27]. Geographic variation within the subspecies is represented by distinct ecotypes [4,11,25,27,28].

In some parts of Europe, native honey bee populations were significantly influenced by human management, including the importation of queens, transhumance movements, and breeding programs [9,11,29,30]. Since the early 20th century, commercial beekeeping has favored non-native subspecies from lineage C, particularly *A. mellifera carnica* and *A. mellifera ligustica*, as well as their hybrids. Beekeepers widely perceived these subspecies as more suitable for apiculture than the native *A. m. mellifera* or found that hybrids between their original M-lineage bees and introduced bees were more productive due to short-term heterosis effects [31,32]. The extensive gene flow between native populations and commercially propagated bees has led to the disappearance or significant genetic erosion of indigenous honey bees in many European regions [33]. In Germany, for instance, the large-scale introduction of non-native queens led to the widespread replacement of *A. m. mellifera* with *A. m. carnica* [34,35]. Similar patterns of replacement or strong introgression have been documented in the Czech Republic [36,37], Denmark [38], and parts of Poland [39,40]. Currently, in many regions of Europe, most professional and amateur beekeepers maintain *A. m. ligustica*, *A. m. carnica*, or their hybrids. Consequently, the natural distribution range of lineage M, particularly *A. m. mellifera*, has substantially decreased in recent decades [10,41], and it continues to be threatened by further introgression.

The degree of genetic introgression varies considerably across northwestern Europe. While many regions exhibit high levels of admixture with C-lineage bees, several areas with relatively low levels of introgression have been identified. These regions include the Iberian Peninsula [42–46], Ireland [47–50], the Inner Hebrides [51,52], northeastern Poland [53], and some protected areas in the Netherlands, Norway, France, Denmark, and Switzerland [25,51,52,54,55]. These regions are therefore considered important genetic refuges for lineage M.

Conserving honey bees is crucial for maintaining their genetic diversity and local adaptations to different environments. Global concern over the loss of honey bee genetic resources has grown in recent years [41], prompting the development of multiple conservation initiatives across Europe [11,56]. One of the most prominent efforts is the International Association for the Conservation of the European Dark Honey Bee (SICAMM), which was established in 1994. SICAMM unites beekeepers, researchers, and conservationists in support of preserving and promoting *A. m. mellifera* across its historical range [57]. Effective conservation management requires reliable, cost-effective, and practical tools to detect introgression between honey bee subspecies. These methods are essential for monitoring genetic integrity, guiding breeding programs, and preventing further introgression in conservation areas. Contemporary methodologies for identifying honey bee subspecies are based on two contrasting approaches: molecular markers and morphometry [27]. Molecular markers, such as microsatellites [58] and, more recently, single nucleotide polymorphisms [28,51,59], constitute a powerful identification toolkit. However, beekeepers' use of these methods is severely limited due to the need for specialized personnel, costly equipment and reagents, and the lack of rapid accessibility, which makes routine colony analysis unaffordable. In contrast, morphometric identification based on wing venation [16,27,60] is a highly cost-effective alternative that can be more easily implemented in apiaries. This approach requires only a microscope with an attached camera to deliver a practical,

affordable tool for beekeepers. Morphometric identification of honey bee subspecies is relatively easy using freely available software [57,61]; however, it requires reference datasets for comparison. Unfortunately, access to publicly available reference data is limited. Paradoxically, there is much more reference data for *A. m. iberiensis* [62] — a well-protected subspecies — than for *A. m. mellifera* [61], an endangered subspecies.

We provide a large amount of data on wing variation in northwestern Europe that can be used to monitor local populations in the future. Furthermore, we demonstrate that the hybridization process of *A. m. mellifera* has accelerated recently, and urgent action is required to prevent the complete extinction of this subspecies.

Country	Number of wings	Number of samples
Belarus	171	5
Cuba	449	9
Germany	60	4
Spain	3482	573
France	164	11
United Kingdom	425	29
Ireland	3498	304
Lithuania	1358	76
Netherlands	3555	125
Norway	2274	84
Poland	22574	900
Portugal	960	192

Table 1. Sample size of honey bee wings used in this study.

Methods

Material

In this study, we provide 29,043 images of honey bee worker wings in a public repository, which is a new contribution. These images represent 1,342 samples from ten countries. Some of these samples were related to previous studies: samples 1–4 from Germany [63]; samples 517–537 from Spain [46]; samples 1–304 from Ireland [50]; samples 640–679 and 680–922 from Poland [64,65]. Material from Belarus, France, United Kingdom, Lithuania, Netherlands, Norway and most material from Poland was not presented in earlier publications. Samples from Belarus were collected in 2016 from log hives in an unprotected population. Samples from France were collected from both log hives and managed hives, which originated from an unprotected population. Samples from the United Kingdom were collected from the protected population on Colonsay island in 2014. Samples from Lithuania were collected from small, traditionally managed apiaries situated in forested regions and remote from apiaries practicing modern beekeeping. Samples from the Netherlands were collected in 2024 from the protected population on Texel island. Samples from Norway were collected from the Flekkefjord conservation area (29 colonies) and other unprotected parts of the country (55 colonies). Most samples from Poland originated from protected populations, which are maintained by honey bee breeders using instrumental insemination. The number of workers per sample in the new dataset ranged from 5 to 69, with an average of 21.6. We also remeasured 449 wings representing nine colonies from Cuba [66,67] and used data from earlier studies related to Poland, Portugal, and Spain [62,68]. In total, we used 38,990 wing images, representing 2,312 samples and 12 countries, in the analysis (Table 1, Fig. 1).

The workers were stored until preparation in ethanol or in freezer. The wings were detached from the bees' bodies and most samples were dry mounted between two microscopic slides. Samples from Netherlands were mounted using Entellan mounting medium. Measurements were taken from both the left and right forewings. If the side was known, it was indicated in the file name with the letters L and R for left and right wings, respectively. The differences between the left and

right wings are relatively small, and their influence on geographic variation is negligible [69]. We used a variety of photographic equipment to acquire the images. Each wing was saved as a separate PNG file. Using the freely available IdentiFly software [61], the coordinates of 19 landmarks were determined in each wing image (Fig. 2). The configuration of landmarks is consistent with that used in earlier studies related to the identification of honey bee lineages and geographic variation in Europe and Asia [68,70]. This enabled us to classify the samples as lineages and extend our dataset with existing data. These landmarks were saved within each wing image file and can be viewed and edited using IdentiFly. The complete dataset, including wing images, landmark coordinates, geographic coordinates of the sampling locations, and supplementary information, is available on the Zenodo website [71].

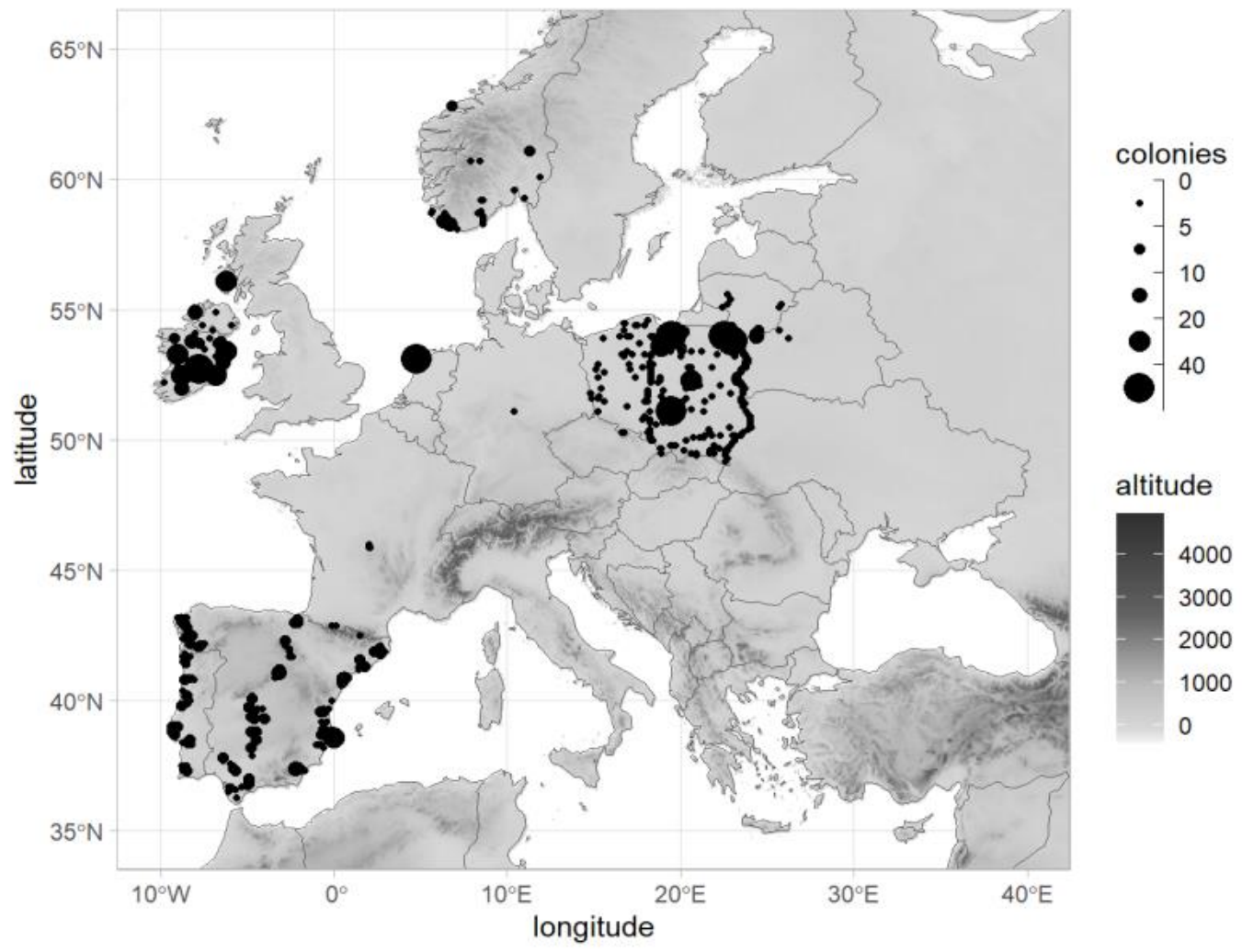

Figure 1. Locations from which honey bee samples were collected.

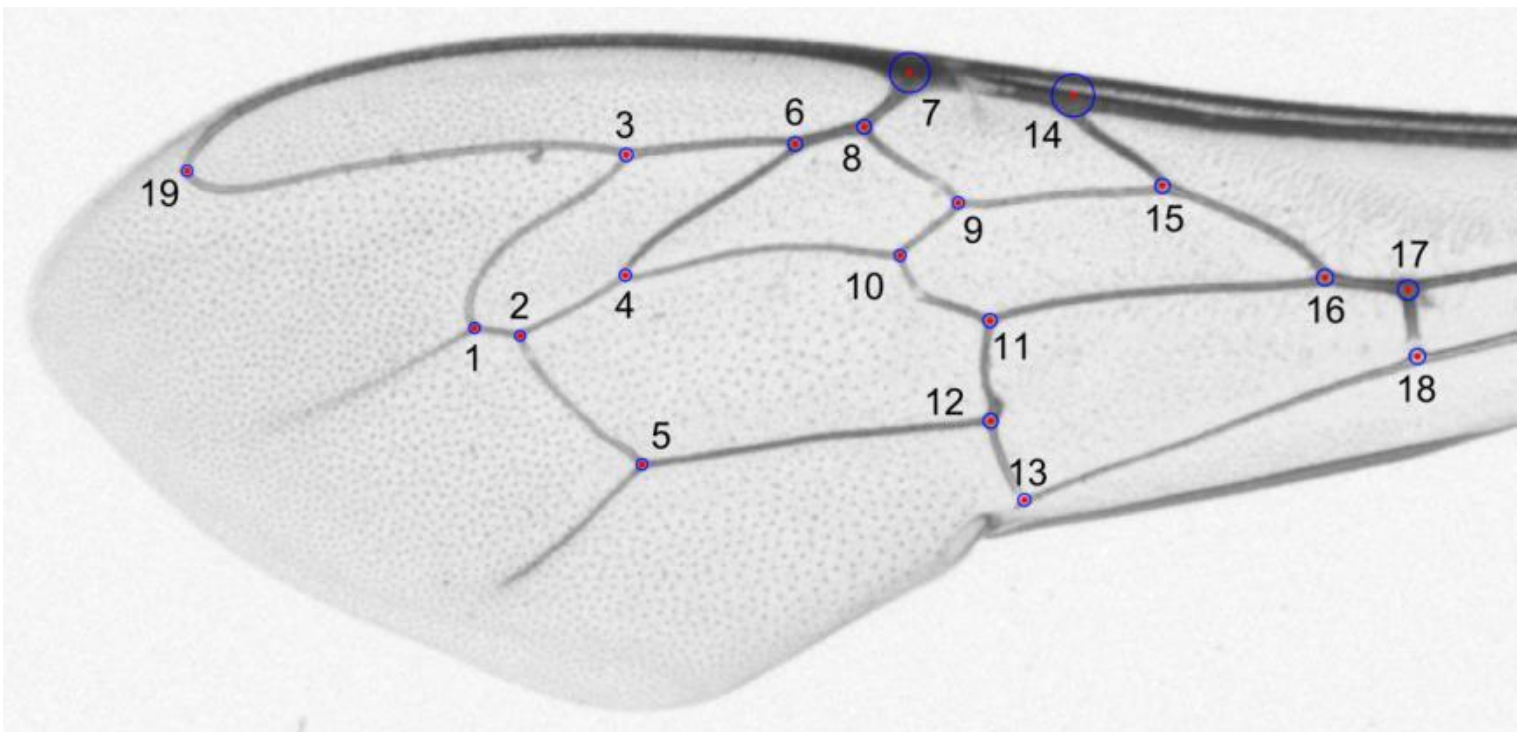

Figure 2. Position of landmarks on the forewing of a honey bee worker. The numbered red dots indicate the landmarks. Blue circles surrounding the dots should be tangent to edges of surrounding venation.

Statistical analysis

A statistical analysis was performed using the R statistical computing environment (version 4.5.0) and the RStudio integrated development environment (version 2025.04.11). The landmarks were analyzed using geometric morphometric methodology. The raw landmark coordinates were aligned using the generalized Procrustes method, which was implemented in the geomorph package (version 4.0.10; [72]. The aligned coordinates were averaged within colonies, and these averages were used in subsequent analyses. Principal component analysis (PCA) was used to reduce the dimensionality of the wing shape data by extracting the first 34 principal components. Because PCA is an unsupervised method, it identifies directions of maximum overall variance without considering group membership. The principal component scores were subsequently used as input for linear

discriminant analysis (LDA) implemented in the Morpho package (v. 2.13) [73]. In contrast to PCA, LDA is a supervised method that uses predefined group labels and identifies linear combinations of variables that reveal differences among groups. To evaluate the ability of the model to discriminate among groups while minimizing the risk of overfitting, classification performance was assessed using leave-one-out cross-validation. In this procedure, each specimen was excluded from the dataset in turn, the linear discriminant model was fitted using all remaining specimens, and the excluded specimen was subsequently classified using the resulting model. The differences in wing shape between countries were described using Mahalanobis distance. The samples were classified as evolutionary lineages using the IdentiFlyR package (v. 0.1.1) [74]. Data from Nawrocka et al. [61] was used as reference samples for the lineage. A supplementary document containing the full details of the statistical analysis is available on the GitHub website.

Results

A principal component analysis of wing shape revealed that honey bee colonies from northwestern Europe formed a single cluster. However, the twelve countries were somewhat separated along the first principal component, which explained 23.4% of the variance (Fig. 3a). The scatterplot showed considerable overlap among the groups, indicating a degree of morphological similarity. The linear discriminant analysis better separated the groups. The graph of the first two linear discriminants shows three clusters: the first consisted of Spain and Portugal; the second, of Ireland and Lithuania; and the third, of the remaining countries (Fig. 3b). Countries that overlapped in the graphs of the first two dimensions differed in other dimensions, and most countries differed markedly from each other in pairwise comparisons (Table 2). The largest Mahalanobis distance was found between samples from Cuba and the United Kingdom, and the smallest distance was found between samples from Portugal and Spain (Table 2). Using cross-validation for classification allowed us to correctly assign 89% of the samples to their respective countries. Most of the misclassifications occurred between neighboring countries, such as Portugal and Spain. However, misclassifications also occurred between distant countries, such as France and Belarus. For more details, see the supplementary document on GitHub.

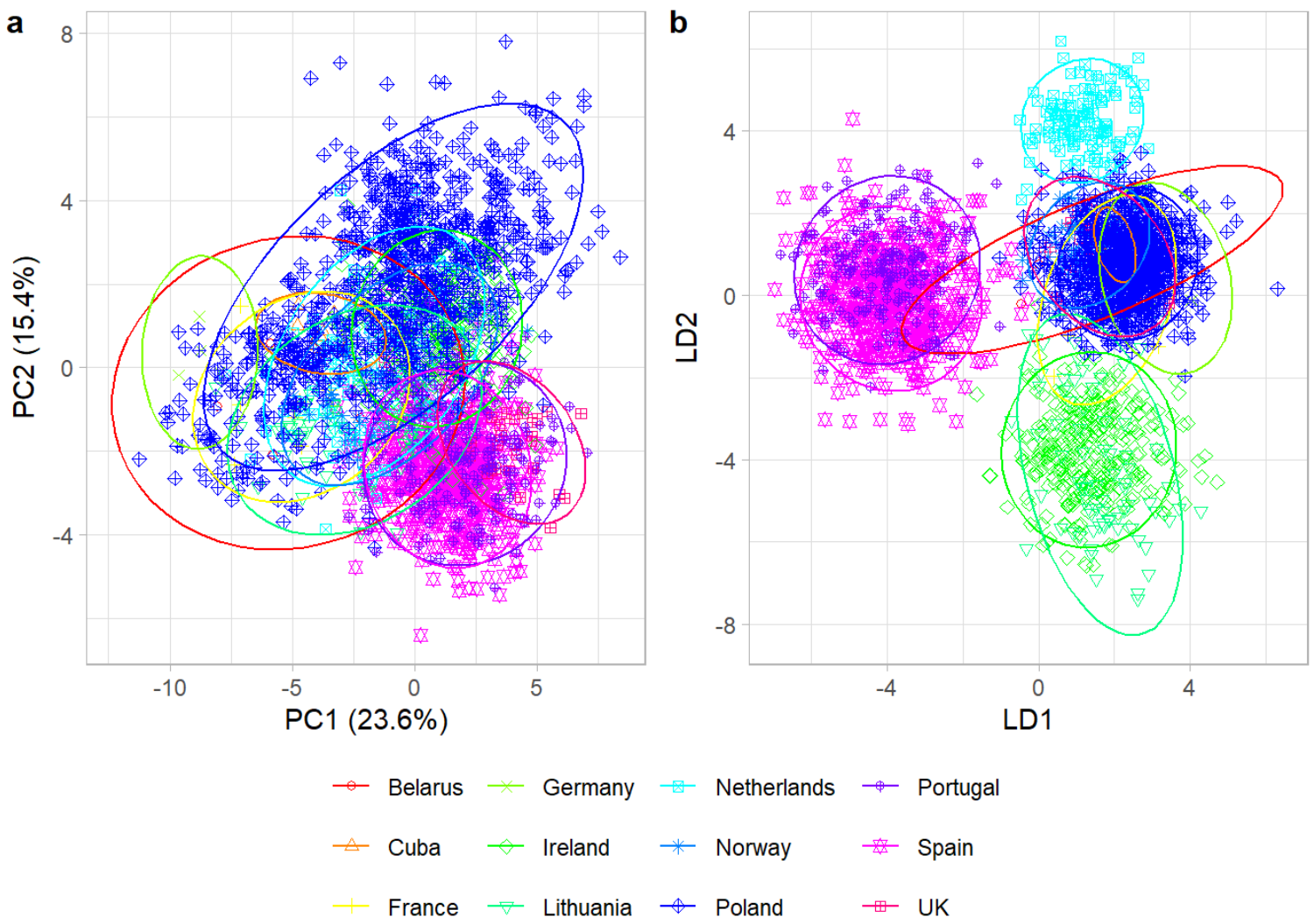

Figure 3. The first two principal components (a) and the first two linear discriminants (b) of honey bee wing shape in northwestern Europe. Each marker represents one colony. Ellipses indicate 95% confidence regions.

Country	BY	CU	DE	ES	FR	UK	IE	LT	NL	NO	PL	PT
BY	-	**	*	****	***	****	****	****	****	**	*	****
CU	6.617	-	****	****	****	****	****	****	****	****	****	****

DE	6.796	9.350	-	****	*	****	****	**	****	****	**	****
ES	6.832	8.722	9.241	-	****	****	****	****	****	****	****	****
FR	6.416	9.002	5.457	7.835	-	****	****	**	****	****	****	****
UK	8.406	10.059	9.826	8.897	9.487	-	****	****	****	****	****	****
IE	8.247	9.527	7.122	8.076	5.391	8.931	-	****	****	****	****	****
LT	7.187	9.641	5.724	7.742	3.648	8.188	3.943	-	****	****	****	****
NL	6.808	8.174	9.346	8.053	8.155	9.297	9.347	8.397	-	****	****	****
NO	5.451	7.346	8.134	6.837	6.253	7.230	7.363	6.750	6.623	-	****	****
PL	4.722	6.548	5.757	6.318	6.008	7.338	6.647	6.215	6.595	5.034	-	****
PT	7.190	8.925	9.601	1.701	8.518	8.957	8.653	8.304	7.964	7.102	6.425	-

Table 2. Differences between countries in wing shape (expressed as Mahalanobis distances, lower triangle) and significance of pairwise comparisons (upper triangle). Country abbreviations: Belarus (BY), Cuba (CU), Germany (DE), Spain (ES), France (FR), the United Kingdom (UK), Ireland (IE), Lithuania (LT), the Netherlands (NL), Norway (NO), Poland (PL), Portugal (PT). Asterisks denote statistical significance: $p < 0.05$ (*), $p < 0.01$ (**), $p < 0.001$ (***), and $p < 0.0001$ (****).

Using reference samples from the Morphometric Bee Database in Oberursel, the colonies were classified into four evolutionary lineages: A, C, M, and O. Most colonies (63.7%) were classified as lineage M. Many were also classified as lineage A (27.5%), C (7.9%), or O (0.8%) (see Fig. 4). Lineage M, which is native to most of the study area, remains prevalent in Portugal (93.8%), Spain (90.8%), Norway (79.8%), Ireland (79.6%), and the Netherlands (73.6%) (Fig. 4). This indicates that conserving honey bee biodiversity within lineage M is feasible. We also found many signs of introgression. Lineage A, native to Africa, was dominant in Belarus and Poland. Lineage C and its hybrids, which are preferred by beekeepers, were dominant in Germany and France.

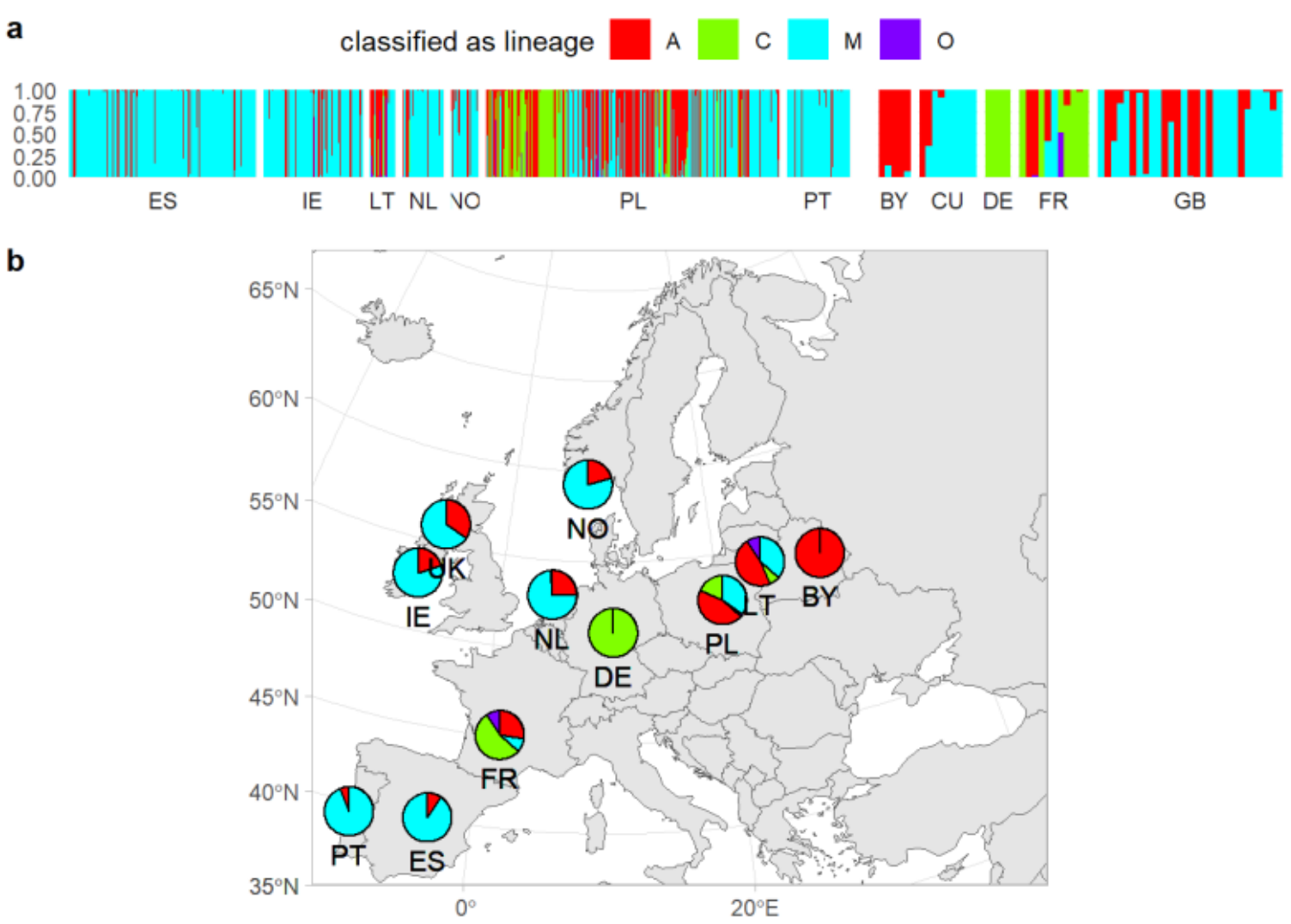

Figure 4. Classification of colonies into four evolutionary lineages, as shown in the admixture graph (a) and map (b). Country abbreviations are the same as in Table 2.

A large amount of data from both protected and unprotected populations in northeastern Poland allows us to investigate the temporal changes in the native honey bee lineage M. Since the beginning of the 21st century, the mean Mahalanobis distance to lineage M increased markedly in northeastern Poland (ANOVA; years: $F_{1,896} = 652.87$, $p < 10^{-15}$; population: $F_{1,896} = 41.51$, $p = 10^{-10}$; interaction: $F_{1,896} = 197.69$, $p < 10^{-15}$). This increase was significant in both protected and unprotected populations, but particularly strong in the unprotected population (Fig. 5). The smaller the similarity to native bees, the larger the distance to lineage M. Therefore, honey bee samples collected after 2016 were significantly less similar to native bees than samples collected prior to that year (Fig. 5).

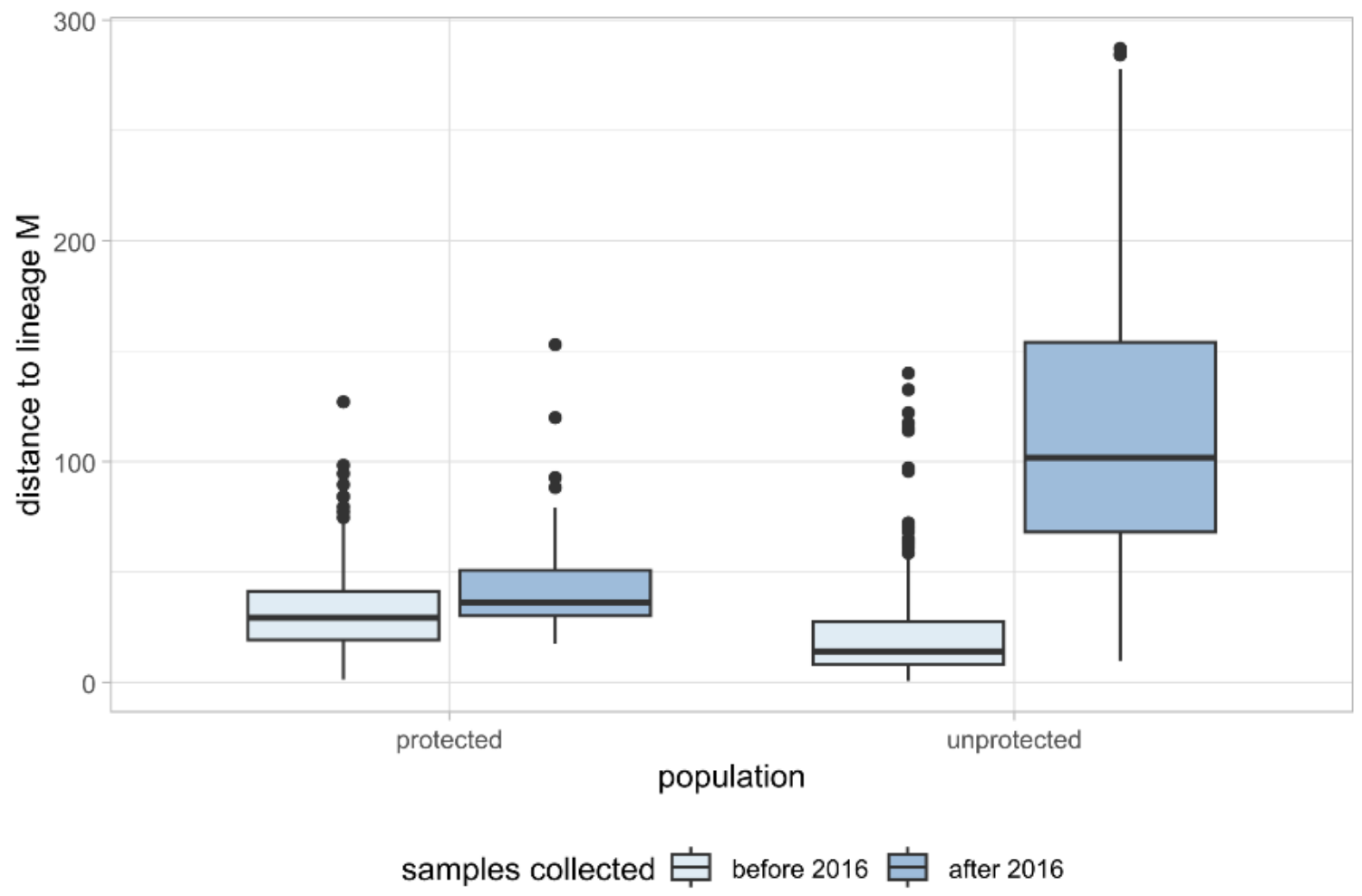

Figure 5. Mahalanobis distance to lineage M for honey bee samples collected in northeastern Poland before and after 2016. The box represents the interquartile range (25th–75th percentiles), the horizontal line indicates the median, the whiskers extend to the most extreme observations within 1.5 × the interquartile range, and points denote outliers.

Discussion

The data presented here show that the proportion of lineage M in northwestern Europe differed markedly between countries (Fig. 4). In Portugal and Spain, more than 90% of colonies are of the native lineage. Samples in this region were collected from unprotected populations, showing that beekeepers use native honey bees. This is the optimal solution for protecting honey bee diversity. When natural nesting sites for honey bee colonies become scarce, beekeepers provide hives for the bees without importing non-native bees. In this region, queen breeders do not use artificial insemination [75]. This allows them to maintain a large population of honey bees that are adapted to the local environment. At the same time, the beekeeping industry in this region is thriving, and honey production is the highest in Europe [76]. A high level of similarity to lineage M is also present in the unprotected population of Ireland [47–50,77]. However, introgression from non-native lineages is larger in this country than in the Iberian Peninsula. In the Netherlands (Texel Island), and the UK (Colonsay Island), and partly Norway protected populations were sampled. In these populations, lineage M is well represented; however, in unprotected populations in these countries, lineage M is most likely much less prevalent [38]. In Poland and Lithuania, where both protected and unprotected populations were sampled, the proportion of lineage M is relatively low, but still present. In Lithuania, a relatively high proportion of colonies were classified as lineage O, which confirms earlier studies [78]. In the unprotected population of France that was sampled, only one of eleven colonies was classified as lineage M; lineage C dominates in this country, though all other lineages are present. An earlier molecular survey in France found a higher proportion of lineage M in both protected and unprotected populations [54]. However, another study revealed a relatively low percentage of lineage M in unprotected populations [79]. None of the colonies investigated in unprotected populations of Belarus were classified as lineage M, although admixture of lineage M was detected in some colonies (Fig. 5a). All five of the colonies investigated in Belarus were classified as lineage A, which occurs naturally in Africa. The mitochondrial DNA characteristic of lineage A is naturally present in the Iberian Peninsula [42], but not in northeastern Europe. A recent mitochondrial DNA survey in Central Europe detected African-ancestry haplotypes at a much lower frequency than reported here [37,40,80]. The high proportion of samples assigned to lineage A may be related to the fact that hybrids between lineages are more likely to be classified as lineage A [68]. These hybrids exhibit an intermediate phenotype

similar to the mean shape of all lineages [81], and they are more effectively detected when molecular markers are used.

In Germany, all of the investigated colonies belonged to lineage C (Fig. 4). These bees originated from free-living colonies in a national park [63]. The process of replacing *A. m. mellifera* with *A. m. carnica* intensified in Germany in the 1940s [82]. By the end of the 20th century, this process had advanced markedly, with most colonies representing hybrids between the two subspecies [82]. Our data suggest that, in some parts of Germany, the replacement of the native subspecies by lineage C is now complete (Fig. 5a). A similar situation occurs in the Czech Republic, where all but a few colonies were classified as *A. m. carnica* [36,37]. The data presented here indicate that *A. m. mellifera* is critically endangered in most of its native range. Surprisingly, lineage M is better preserved in Cuba, where *Apis mellifera* is not native, than in most of its native range in northwestern Europe [66,83].

There is significant geographic variability in the wing shape of honey bees in northwestern Europe. Within lineage M, two European subspecies were declared: *A. m. iberiensis* and *A. m. mellifera* [16]. The Pyrenees Mountains, a natural barrier to gene flow, lie between the two subspecies. Our results confirm the distinction between these two subspecies. Interestingly, mainly *A. m. mellifera* suffers from introgression with non-native bees. Significant differences in wing shape were also detected among countries within this subspecies, consistent with patterns previously identified using molecular markers [28]. This diversity may be related to natural variation shaped by natural selection [16] as well as beekeeping practices, including the introduction of commercial honey bees through breeding programs, importing queens, and transhumance movements [11]. Understanding which part of this variation is natural or human-induced is important for the adoption of optimal conservation strategies. Given the very large distribution area of *A. m. mellifera* and the various climatic conditions within it, it is highly probable that numerous ecotypes adapted to the local environment were present [27]. Some of these ecotypes, such as those occurring naturally in Germany and Czech Republic, could be irreversibly lost. The best conservation strategy is probably to protect local bees, even if they differ from the historic populations of a particular area. According to this strategy, it is better to avoid the long-distance reintroduction of *A. m. mellifera*, as they most likely represent different ecotypes.

Some conservation strategies rely on artificial selection for phenotypic traits considered characteristic of native honey bee populations. For example, colonies may be selected based on the cubital index, a ratio of the lengths of two wing veins [84]. However, such an approach targets only the selected trait, while many other characteristics that contribute to local adaptation may not be maintained. This limitation is not unique to morphometric approaches. A similar problem arises when conservation is based on a limited number of genetic markers, as selection for those markers alone does not preserve the remainder of the genome that may contribute to adaptation to local environmental conditions.

A more effective strategy for conserving local *A. m. mellifera* ecotypes can be achieved by designating large areas where the introduction of non-local bees is prohibited [52]. Within such areas, natural selection can maintain genotypes that are adapted to local environmental conditions. Wing morphometry can support this strategy by providing an effective method for identifying colonies that have been recently introduced from other country. The selective removal of such colonies would help preserve the integrity of local populations without imposing artificial positive selection for a predefined set of morphological or genetic characteristics.

It is essential to describe the current variation and monitor how it changes. Originally, morphometry was used to describe the geographic variation of honey bees and differentiate subspecies. Later, molecular markers were introduced, improving subspecies discrimination. However, obtaining reference samples for molecular studies can be problematic in some parts of Europe [79] because populations are admixed, making it unclear which aspects of geographic variation are natural and which are related to beekeeping activities. In these cases, morphometric data can be used for

cross-validation. Unlike molecular markers, morphometry is accessible to beekeepers; therefore, it can serve as a quick, inexpensive method for monitoring endangered populations. An important source of reference data is the Morphometric Bee Data Bank in Oberursel, Germany. Unfortunately, the currently available lineage data is limited in size [61], and further studies are needed to expand upon it. The data provided here can be used for future comparisons.

We used data from northeastern Poland to analyze changes in similarity to lineage M in recent times. This region was the last refuge of *A. m. mellifera* in Poland. The results are alarming, as the similarity to lineage M decreased markedly in the unprotected population (Fig. 5). The situation is much better in the protected population, which indicates that conservation programs are essential for the protection of the remaining *A. m. mellifera* populations. The rapid changes in recent years demonstrate the urgent need for action to prevent further erosion of honey bee genetic diversity in northwestern Europe. As has been shown, the introduction of bees maladapted to local environment into new areas causes increased mortality rates and contributes to bee losses [3]. The process of replacement of local populations of honey bees is supported within the current European Union Common Agricultural Policy giving funding to “rationalising transhumance” (Regulation (EU) 2021/2115, Article 55). A much more responsible action would enforce a moratorium on long distance bee movement between honey bees of different subspecies to minimize further loss of genetic diversity and avoid colony losses.

Availability of Source Code and Requirements

Project name: Apis-mellifera-NW-Europe
Project homepage: https://github.com/DrawWing/Apis-mellifera-NW-Europe, including the analysis scripts, statistics, and visualizations generated from the project
Operating system(s): Platform independent
Programming language: R
Other requirements: R (v. 4.5.0), numerous R packages described in the Methods section
License: GPL-3.0
WorkflowHub: https://workflowhub.eu/workflows/2226
Bio.tools: apis-wings-nw-europe

List of Abbreviations

BY: Belarus, CU: Cuba, DE: Germany, ES: Spain, FR: France, UK: the United Kingdom, IE: Ireland, LDA: linear discriminant analysis, LT: Lithuania, NL: the Netherlands, NO: Norway, PCA: principal component analysis, PL: Poland, PT: Portugal.

Data Availability

The complete dataset, including wing images, landmark coordinates, geographic coordinates of sampling locations and other data, is available on the Zenodo website [71].

Author Contributions

Julita Machlowska (Writing—review & editing, Writing—original draft, Data Curation), Dariusz Gerula (Writing—review & editing, Resources, Data Curation), Andrzej Oleksa (Writing—review & editing, Resources, Data Curation), Henk Kok (Writing—review & editing, Resources, Data Curation), Grace P. McCormack (Writing—review & editing, Resources, Data Curation), Alexandra Valentine (Writing—review & editing, Resources, Data Curation), Lars Kirkerud (Writing—review & editing, Resources, Data Curation), Per Kryger (Writing—review & editing, Resources, Data Curation), Laima Blažytė-Čereškienė (Writing—review & editing, Resources, Data Curation), Martin Hasselmann (Writing—review & editing, Resources, Data Curation), Teweldemedhn Gebretinsae Hailu (Writing—review & editing, Resources, Data Curation), Benjamin Rutschmann (Writing—review & editing, Resources, Data Curation), Adam Tofilski (Writing—review & editing, Writing—original draft, Conceptualization, Formal Analysis, Methodology)

Funding

This research was funded by National Science Centre, Poland, grant numbers: 2021/41/B/NZ9/03153 and 2024/06/Y/NZ9/00137.

Acknowledgements

Andrew Abrahams provided bees from his apiary on Colonsay island. Patrick L. Kohl helped in collecting samples in Germany and Spain. We thank Adam Wright and Karin Maassen (Custos Apium) and Lucy Tournadre (Propriétaire Courcelles project) for access to samples in France and assistance with wing sampling. Jarosław Prabucki, now deceased, provided some samples from Poland. DeepL software was used to correct English language. The AI tool was employed solely for language refinement and did not influence the empirical data, analyses, or conclusions of the study.

Conflicts of Interest

The authors declare no conflicts of interest.

References

1. Calderone NW. Insect pollinated crops, insect pollinators and US agriculture: trend analysis of aggregate data for the period 1992–2009. *PLOS ONE*. 2012;7:e37235. 10.1371/journal.pone.0037235

2. Hung K-LJ, Kingston JM, Albrecht M, Holway DA, Kohn JR. The worldwide importance of honey bees as pollinators in natural habitats. *Proc R Soc B Biol Sci*. 2018;285:20172140. 10.1098/rspb.2017.2140

3. Büchler R, Costa C, Hatjina F, Andonov S, Meixner MD, Conte YL, et al. The influence of genetic origin and its interaction with environmental effects on the survival of *Apis mellifera* L. colonies in Europe. *J Apic Res*. 2014;53:205–14. 10.3896/IBRA.1.53.2.03

4. Strange JP, Garnery L, Sheppard WS. Persistence of the Landes ecotype of *Apis mellifera mellifera* in southwest France: confirmation of a locally adaptive annual brood cycle trait. *Apidologie*. 2007;38:259–67. 10.1051/apido:2007012

5. Potts SG, Roberts SPM, Dean R, Marris G, Brown MA, Jones R, et al. Declines of managed honey bees and beekeepers in Europe. *J Apic Res*. 2010;49:15–22. 10.3896/IBRA.1.49.1.02

6. vanEngelsdorp D, Evans JD, Saegerman C, Mullin C, Haubruge E, Nguyen BK, et al. Colony collapse disorder: a descriptive study. *PLOS ONE*. 2009;4:e6481. 10.1371/journal.pone.0006481

7. Kohl PL, Rutschmann B. The wild honeybee population of Europe is in decline: evidence from monitoring studies. *Conserv Sci Pract*. 2026;8:e70345. 10.1111/csp2.70345

8. Panziera D, Requier F, Chantawannakul P, Pirk CWW, Blacquiere T. The diversity decline in wild and managed honey bee populations urges for an integrated conservation approach. *Front Ecol Evol*. 2022; 10.3389/fevo.2022.767950

9. Themudo GE, Rey-Iglesia A, Robles Tascón L, Bruun Jensen A, da Fonseca RR, Campos PF. Declining genetic diversity of European honeybees along the twentieth century. *Sci Rep*. 2020;10:10520. 10.1038/s41598-020-67370-2

10. vanEngelsdorp D, Meixner MD. A historical review of managed honey bee populations in Europe and the United States and the factors that may affect them. *J Invertebr Pathol*. 2010;103:S80–95. 10.1016/j.jip.2009.06.011

11. De la Rúa P, Jaffé R, Dall'Olio R, Muñoz I, Serrano J. Biodiversity, conservation and current threats to European honeybees. *Apidologie*. 2009;40:263–84. 10.1051/apido/2009027

12. Meixner MD, Costa C, Kryger P, Hatjina F, Bouga M, Ivanova E, et al. Conserving diversity and vitality for honey bee breeding. *J Apic Res*. 2010;49:85–92. 10.3896/IBRA.1.49.1.12

13. Moritz RFA, Härtel S, Neumann P. Global invasions of the western honeybee (*Apis mellifera*) and the consequences for biodiversity. *Écoscience*. 2005;12:289–301. 10.2980/i1195-6860-12-3-289.1

14. Whitfield CW, Behura SK, Berlocher SH, Clark AG, Johnston JS, Sheppard WS, et al. Thrice out of Africa: ancient and recent expansions of the honey bee, *Apis mellifera*. *Science*. 2006;314:642–5. 10.1126/science.1132772

15. Engel MS. The taxonomy of recent and fossil honey bees (Hymenoptera : Apidae ; *Apis*). *J Hymenopt Res*. 1999;8:165–96. http://www.pensoft.net/journals/jhr/

16. Ruttner F. Biogeography and taxonomy of honeybees. Berlin: Springer; 1988. 10.1007/978-3-642-72649-1

17. Sheppard WS, Arias MC, Grech A, Meixner MD. *Apis mellifera ruttneri*, a new honey bee subspecies from Malta. *Apidologie*. 1997;28:287–93. 10.1051/apido:19970505

18. Sheppard WS, Meixner MD. *Apis mellifera pomonella*, a new honey bee subspecies from Central Asia. *Apidologie*. 2003;34:367–75. 10.1051/apido:2003037

19. Franck P, Garnery L, Solignac M, Cornuet J-M. Molecular confirmation of a fourth lineage in honeybees from the Near East. *Apidologie*. 2000;31:167–80. 10.1051/apido:2000114

20. Hailu TG, D'Alvise P, Tofilski A, Fuchs S, Greiling J, Rosenkranz P, et al. Insights into Ethiopian honey bee diversity based on wing geomorphometric and mitochondrial DNA analyses. *Apidologie*. 2020; 10.1007/s13592-020-00796-9

21. Alburaki M, Moulin S, Legout H, Alburaki A, Garnery L. Mitochondrial structure of Eastern honeybee populations from Syria, Lebanon and Iraq. *Apidologie*. 2011;42:628–41. 10.1007/s13592-011-0062-4

22. Franck P, Garnery L, Loiseau A, Oldroyd BP, Hepburn HR, Solignac M, et al. Genetic diversity of the honeybee in Africa: microsatellite and mitochondrial data. *Heredity*. 2001;86:420–30. 10.1046/j.1365-2540.2001.00842.x

23. Chen C, Liu Z, Pan Q, Chen X, Wang H, Guo H, et al. Genomic analyses reveal demographic history and temperate adaptation of the newly discovered honey bee subspecies *Apis mellifera sinisxinyuan* n. ssp. *Mol Biol Evol*. 2016;33:1337–48. 10.1093/molbev/msw017

24. Crane E. The World History of Beekeeping and Honey Hunting. New York: Routledge; 1999. 10.4324/9780203819937

25. Francis RM, Kryger P, Meixner M, Bouga M, Ivanova E, Andonov S, et al. The genetic origin of honey bee colonies used in the COLOSS genotype-environment interactions experiment: a comparison of methods. *J Apic Res*. 2014;53:188–204. 10.3896/IBRA.1.53.2.02

26. Ilyasov RA, Lee M, Takahashi J, Kwon HW, Nikolenko AG. A revision of subspecies structure of western honey bee *Apis mellifera*. *Saudi J Biol Sci*. 2020;27:3615–21. 10.1016/j.sjbs.2020.08.001

27. Meixner MD, Pinto MA, Bouga M, Kryger P, Ivanova E, Fuchs S. Standard methods for characterising subspecies and ecotypes of *Apis mellifera*. *J Apic Res*. 2013;52:1–28. 10.3896/IBRA.1.52.4.05

28. Momeni J, Parejo M, Nielsen RO, Langa J, Montes I, Papoutsis L, et al. Authoritative subspecies diagnosis tool for European honey bees based on ancestry informative SNPs. *BMC Genomics*. 2021;22:101. 10.1186/s12864-021-07379-7

29. Leroy T, Faux P, Basso B, Eynard S, Wragg D, Vignal A. Inferring long-term and short-term determinants of genetic diversity in honey bees: beekeeping impact and conservation strategies. *Mol Biol Evol*. 2024;41:msae249. 10.1093/molbev/msae249

30. Soland-Reckeweg G, Heckel G, Neumann P, Fluri P, Excoffier L. Gene flow in admixed populations and implications for the conservation of the western honeybee, *Apis mellifera*. *J Insect Conserv*. 2009;13:317–28. 10.1007/s10841-008-9175-0

31. Cale GH Jr, Gowen JW. Heterosis in the honey bee (*Apis mellifera* L.). *Genetics*. 1956;41:292–303. 10.1093/genetics/41.2.292

32. Fresnaye J, Lavie P, Boesiger E. La variabilité de la production du miel chez l'abeille de race noire (*Apis mellifica* L.) et chez quelques hybrides interraciaux. *Apidologie*. 1974;5:1–20. 10.1051/apido:19740101

33. Peer DF. Further studies on the mating range of the honey bee, *Apis mellifera* L. *Can Entomol*. 1957;89:108–10. 10.4039/Ent89108-3

34. Kauhausen-Keller D, Keller R. Morphometrical control of pure race breeding in the honeybee (*Apis mellifera* L). *Apidologie*. 1994;25:133–43. 10.1051/apido:19940202

35. Maul V, Hähnle A. Morphometric studies with pure bred stock of *Apis mellifera carnica* Pollmann from Hessen. *Apidologie*. 1994;25:119–32. 10.1051/apido:19940201

36. Knoll A, Šotek M, Prouza J, Langová L, Přidal A, Urban T, et al. Assessing genetic diversity and population structure of western honey bees in the Czech Republic using 22 microsatellite loci. *Insects*. 2025;16. 10.3390/insects16010055

37. Knoll A, Langová L, Přidal A, Urban T, Knoll A, Langová L, et al. Haplotype diversity in mtDNA of honeybee in the Czech Republic confirms complete replacement of autochthonous population with the C lineage. *Insects*. 2024;15. 10.3390/insects15070495

38. Nielsdatter MG, Larsen M, Nielsen LG, Nielsen MM, Rasmussen C. History of the displacement of the European dark bee (*Apis mellifera mellifera*) in Denmark. *J Apic Res*. 2021;60:13–8. 10.1080/00218839.2020.1826111

39. Bieńkowska M, Splitt A, Węgrzynowicz P, Maciorowski R. The buzz changes within time: native *Apis mellifera mellifera* honeybee subspecies less and less popular among Polish beekeepers since 1980. *Agriculture*. 2021;11:652. 10.3390/agriculture11070652

40. Oleksa A, Kusza S, Tofilski A. Mitochondrial DNA suggests the introduction of honeybees of African ancestry to east-central Europe. *Insects*. 2021;12:410. 10.3390/insects12050410

41. Requier F, Garnery L, Kohl PL, Njovu HK, Pirk CWW, Crewe RM, et al. The conservation of native honey bees is crucial. *Trends Ecol Evol*. 2019;34:789–98. 10.1016/j.tree.2019.04.008

42. Cánovas F, De la Rúa P, Serrano J, Galián J. Geographical patterns of mitochondrial DNA variation in *Apis mellifera iberiensis* (Hymenoptera: Apidae). *J Zool Syst Evol Res*. 2008;46:24–30. 10.1111/j.1439-0469.2007.00435.x

43. Chávez-Galarza J, Henriques D, Johnston JS, Carneiro M, Rufino J, Patton JC, et al. Revisiting the Iberian honey bee (*Apis mellifera iberiensis*) contact zone: maternal and genome-wide nuclear variations provide support for secondary contact from historical refugia. *Mol Ecol*. 2015;24:2973–92. 10.1111/mec.13223

44. De la Rúa P, Jiménez Y, Galián J, Serrano J. Evaluation of the biodiversity of honey bee (*Apis mellifera*) populations from eastern Spain. *J Apic Res*. 2004;43:162–6. 10.1080/00218839.2004.11101130

45. Pinto MA, Henriques D, Neto M, Guedes H, Muñoz I, Azevedo JC, et al. Maternal diversity patterns of Ibero-Atlantic populations reveal further complexity of Iberian honeybees. *Apidologie*. 2013;44:430–9. 10.1007/s13592-013-0192-y

46. Rutschmann B, Kohl PL, Machado A, Steffan-Dewenter I. Semi-natural habitats promote winter survival of wild-living honeybees in an agricultural landscape. *Biol Conserv*. 2022;266:109450. 10.1016/j.biocon.2022.109450

47. Browne KA, Hassett J, Geary M, Moore E, Henriques D, Soland-Reckeweg G, et al. Investigation of free-living honey bee colonies in Ireland. *J Apic Res*. 2020;60:229–40. 10.1080/00218839.2020.1837530

48. Hassett J, Browne KA, McCormack GP, Moore E, Society NIHB, Soland G, et al. A significant pure population of the dark European honey bee (*Apis mellifera mellifera*) remains in Ireland. *J Apic Res*. 2018;57:337–50. 10.1080/00218839.2018.1433949

49. Jensen AB, Palmer KA, Boomsma JJ, Pedersen BV. Varying degrees of *Apis mellifera ligustica* introgression in protected populations of the black honeybee, *Apis mellifera mellifera*, in northwest Europe. *Mol Ecol*. 2005;14:93–106. 10.1111/j.1365-294X.2004.02399.x

50. Valentine A, Moro A, Briggs E, Collier B, Sandoval K, Binetti C, et al. Introgressive hybridisation puts the distinctive population of Apis mellifera mellifera in Ireland at risk: evidence from a multidisciplinary approach. *J Apic Res*. 2025;64:202–16. 10.1080/00218839.2024.2404297

51. Henriques D, Browne KA, Barnett MW, Parejo M, Kryger P, Freeman TC, et al. High sample throughput genotyping for estimating C-lineage introgression in the dark honeybee: an accurate and cost-effective SNP-based tool. *Sci Rep*. 2018;8:8552. 10.1038/s41598-018-26932-1

52. Pinto MA, Henriques D, Chávez-Galarza J, Kryger P, Garnery L, Zee R van der, et al. Genetic integrity of the Dark European honey bee (*Apis mellifera mellifera*) from protected populations: a genome-wide assessment using SNPs and mtDNA sequence data. *J Apic Res*. 2014;53:269–78. 10.3896/IBRA.1.53.2.08

53. Oleksa A, Chybicki I, Tofilski A, Burczyk J. Nuclear and mitochondrial patterns of introgression into native dark bees (*Apis mellifera mellifera*) in Poland. *J Apic Res*. 2011;50:116–29. 10.3896/IBRA.1.50.2.03

54. Bertrand B, Alburaki M, Legout H, Moulin S, Mougel F, Garnery L. MtDNA COI-COII marker and drone congregation area: an efficient method to establish and monitor honeybee (*Apis mellifera* L.) conservation centres. *Mol Ecol Resour*. 2015;15:673–83. 10.1111/1755-0998.12339

55. Parejo M, Henriques D, Pinto MA, Soland-Reckeweg G, Neuditschko M. Empirical comparison of microsatellite and SNP markers to estimate introgression in *Apis mellifera mellifera*. *J Apic Res*. 2018;57:504–6. 10.1080/00218839.2018.1494894

56. Muñoz I, Henriques D, Johnston JS, Chávez-Galarza J, Kryger P, Pinto MA. Reduced SNP panels for genetic identification and introgression analysis in the dark honey bee (*Apis mellifera mellifera*). *PLOS ONE*. 2015;10:e0124365. 10.1371/journal.pone.0124365

57. García CAY, Rodrigues PJ, Tofilski A, Elen D, McCormak GP, Oleksa A, et al. Using the software DeepWings© to classify honey bees across Europe through wing geometric morphometrics. *Insects*. 2022;13:1132. 10.3390/insects13121132

58. Muñoz I, Henriques D, Jara L, Johnston JS, Chávez-Galarza J, De La Rúa P, et al. SNPs selected by information content outperform randomly selected microsatellite loci for delineating genetic identification and introgression in the endangered dark European honeybee (*Apis mellifera mellifera*). *Mol Ecol Resour*. 2017;17:783–95. 10.1111/1755-0998.12637

59. Chen C, Parejo M, Momeni J, Langa J, Nielsen RO, Shi W, et al. Population structure and diversity in European honey bees (*Apis mellifera* L.) - an empirical comparison of pool and individual whole-genome sequencing. *Genes*. 2022;13:182. 10.3390/genes13020182

60. Kandemir İ, Özkan A, Fuchs S. Reevaluation of honeybee (*Apis mellifera*) microtaxonomy: a geometric morphometric approach. *Apidologie*. 2011;42:618–27. 10.1007/s13592-011-0063-3

61. Nawrocka A, Kandemir İ, Fuchs S, Tofilski A. Computer software for identification of honey bee subspecies and evolutionary lineages. *Apidologie*. 2018; 10.1007/s13592-017-0538-y

62. Oleksa A, Căuia E, Siceanu A, Puškadija Z, Kovačić M, Pinto MA, et al. Collection of wing images for conservation of honey bees (*Apis mellifera*) biodiversity in Europe. *Zenodo*. 2022; http://doi.org/10.5281/zenodo.7244070

63. Kohl PL, Rutschmann B. The neglected bee trees: European beech forests as a home for feral honey bee colonies. *PeerJ*. 2018;6:e4602. 10.7717/peerj.4602

64. Oleksa A, Gawroński R, Tofilski A. Rural avenues as a refuge for feral honey bee population. *J Insect Conserv*. 2013;17:465–72. 10.1007/s10841-012-9528-6

65. Tofilski A. Using geometric morphometrics and standard morphometry to discriminate three honeybee subspecies. *Apidologie*. 2008;39:558–63. 10.1051/apido:2008037

66. Masaquiza D, Ferrán MO, Guamán S, Naranjo E, Vaca M, Curbelo LM, et al. Geometric morphometric analysis of wing shape to identify populations of *Apis mellifera* in Camagüey, Cuba. *Insects*. 2023;14:306. 10.3390/insects14030306

67. Masaquiza D, Ferran MO, Arenal CA. Collection of images of honey bee wings (*Apis mellifera*) from Cuba. 2023; http://doi.org/10.5281/zenodo.7662013

68. Oleksa A, Căuia E, Siceanu A, Puškadija Z, Kovačić M, Pinto MA, et al. Honey bee (*Apis mellifera*) wing images: a tool for identification and conservation. *GigaScience*. 2023;12:giad019. 10.1093/gigascience/giad019

69. Janczyk A, Tofilski A. Monthly changes in honey bee forewings estimated using geometric morphometrics. *J Apic Sci*. 2021;65:139–46. 10.2478/jas-2021-0002

70. Machlowska J, Kandemir I, Özkan Koca A, et al (2025) Variation of honey bee wings in southwestern Asia. Sci Data 12:1979. 10.1038/s41597-025-06234-8

71. Machlowska J, Gerula D, Oleksa A, Kok H, McCormack G, Valentine A, et al. Fore wings of honey bees (*Apis mellifera*) from northwestern Europe. *Zenodo*. 2026; http://doi.org/10.5281/zenodo.18845767

72. Baken EK, Collyer ML, Kaliontzopoulou A, Adams DC. geomorph v4.0 and gmShiny: enhanced analytics and a new graphical interface for a comprehensive morphometric experience. *Methods Ecol Evol*. 2021;12:2355–63. 10.1111/2041-210X.13723

73. Schlager S. Morpho and Rvcg – shape analysis in R. In: Zheng G, Li S, Székely G, editors. Statistical shape and deformation analysis. London: Academic Press; 2017. p. 217–256. 10.1016/C2015-0-06799-5

74. Kaur H, Ganie SA, Tofilski A. Morphometric identification of an unknown honey bee colony: an example from north India. *J Apic Sci*. 2024;155–66. 10.2478/JAS-2024-0013

75. Lodesani M, Costa C. Bee breeding and genetics in Europe. *Bee World*. 2003;84:69–85. 10.1080/0005772X.2003.11099579

76. Jones R. European beekeeping in the 21st century: strengths, weaknesses, opportunities, threats. *Bee World*. 2004;85:77–80. 10.1080/0005772X.2004.11099637

77. Garcia CAY, Henriques D, Cilia G, Rufino J, Vella C, Aglagane A, et al. Contrasting whole-genome diversity patterns and adaptation in honeybees from two southern European glacial refugia. *iScience*. 2026;29. 10.1016/j.isci.2025.114497

78. Ceksteryte V, Paplauskiene V, Tamasauskiene D, Pasakinskiene I, Mazeikiene I. Genetic characterization of Lithuanian honeybee lines based on ISSR polymorphism. *Apidologie*. 2012;43:652–62. 10.1007/s13592-012-0140-2

79. Wragg D, Marti-Marimon M, Basso B, Bidanel J-P, Labarthe E, Bouchez O, et al. Whole-genome resequencing of honeybee drones to detect genomic selection in a population managed for royal jelly. *Sci Rep*. 2016;6:27168. 10.1038/srep27168

80. Orlovskytė S, Budrys E, Skrodenytė-Arbačiauskienė V, Blažytė-Čereškienė L. The dark European honey bee *Apis mellifera mellifera* in Lithuania: data on mitotype diversity of native bee population. *J Apic Res*. 2025;64:959–62. 10.1080/00218839.2024.2327125

81. Węgrzynowicz P, Gerula D, Tofilski A, Panasiuk B, Bieńkowska M. Maternal inheritance in hybrids of three honey bee subspecies. *J Apic Sci*. 2019;63:131–8. 10.2478/jas-2019-0010

82. Moritz RFA. The limitations of biometric control on pure race breeding in *Apis mellifera*. *J Apic Res*. 1991;30:54–9. 10.1080/00218839.1991.11101234

83. García CAY, Luis AR, Pérez Piñeiro A, Pérez Morfi A, Invernizzi C, Tomasco IH. Cuban honey bees: significant differentiation from European honey bees in incomplete isolation. *J Apic Res*. 2021;60:375–84. 10.1080/00218839.2020.1841460

84. Ruttner F. Breeding techniques and selection for breeding of the honeybee. Derby: British Isles Bee Breeders' Association, 1988.
