# Supplementary material for: Country specific hybridization of honey bees from lineage M
Source: Gigascience. 2026 Sep 15;15:giag093. doi: 10.1093/gigascience/giag093 (PMC13615543; doi:10.1093/gigascience/giag093)
Supplement: Supplementary file 4 [file giag093_reviewer_1_report_original_submission.pdf]

Reviewer Report

Title: Country specific hybridization of honey bees from lineage M

Version: Original Submission **Date:** 4/27/2026

Reviewer name: Reviewer 1

Reviewer Comments to Author:

The manuscript GIGA-D-26-00106, entitled "Country-specific hybridization of honey bees from lineage M," analyzes Apis mellifera samples from 12 countries and applies geometric morphometry (GM) of the wing to classify honey bees for discrimination purposes.

This manuscript addresses an important and timely topic in bee discrimination. However, several issues should be considered before a final decision is made.

1. The use of geometric morphometry (GM) for lineage discrimination (A, M, etc.) is appropriate and well established. However, in the absence of genomic validation on the same specimens, the assignment of individuals to evolutionary lineages remains indirect. Reliance on previously published classifications introduces uncertainty, particularly in the context of hybridization. I recommend that the authors either provide genetic validation for the analyzed samples or explicitly discuss this limitation and its potential impact on classification accuracy.
2. Figure 5. How can the sampling time categories ("before 2016" and "after 2016") be interpreted? How can temporal changes in protected and unprotected samples be evaluated from this figure?
3. The observed hybridization patterns may be influenced not only by classification outcomes but also by spatial structure, landscape connectivity, and beekeeping practices. Natural dispersal via swarming is typically limited in range, whereas long-distance gene flow is often human-mediated. It would be important to discuss potential geographic or ecological barriers to dispersal, as well as management practices (e.g., migratory beekeeping) that may affect hybridization. Without considering these factors, the classification remains largely descriptive.
4. The conclusion is unclear. PCA indicates substantial overlap between groups, whereas the same data appear to show separation under a supervised model. This discrepancy requires clarification. Please explain how the supervised model achieves separation despite the overlap observed in PCA, and provide details on model validation (e.g., cross-validation, accuracy, or overfitting control) to support its reliability.
5. The Methods section lacks critical detail. It is not clear which wing was analyzed (right, left, or both). Additionally, the rationale for selecting the 12 countries is not provided. What is the history and intensity of beekeeping in these regions, and how might this influence the results?
6. There is also a substantial imbalance in sample sizes (e.g., ~900 samples from Poland versus ~5 from Belarus). This raises concerns regarding statistical robustness, potential bias in classification performance, and the reliability of conclusions for underrepresented groups. Please clarify how these issues were addressed and discuss the associated limitations.
7. Furthermore, although a COLOSS standard morphometric protocol is available (Meixner et al., 2012), it is not referenced or discussed. The numbering and ordering of landmarks also appear to differ from

this standard. Please clarify which protocol was followed and justify any deviations.
Meixner et al., 2012:

.

8. Line 328: Why do the authors conclude that maladapted bees introduced into new areas carry "poor" genes? This statement appears speculative and requires clarification and supporting evidence.
Overall, I recommend major revisions, as several key methodological and interpretative issues need to be addressed to support the conclusions.

Methods

Are the methods appropriate to the aims of the study, are they well described, and are necessary controls included? Choose an item.

Conclusions

Are the conclusions adequately supported by the data shown? Choose an item.

Reporting Standards

Does the manuscript adhere to the journal's guidelines on minimum standards of reporting? Choose an item.

Choose an item.

Statistics

Are you able to assess all statistics in the manuscript, including the appropriateness of statistical tests used? Choose an item.

Quality of Written English

Please indicate the quality of language in the manuscript: Choose an item.

Declaration of Competing Interests

Please complete a declaration of competing interests, considering the following questions:

- Have you in the past five years received reimbursements, fees, funding, or salary from an organisation that may in any way gain or lose financially from the publication of this manuscript, either now or in the future?
- Do you hold any stocks or shares in an organisation that may in any way gain or lose financially from the publication of this manuscript, either now or in the future?
- Do you hold or are you currently applying for any patents relating to the content of the manuscript?

- Have you received reimbursements, fees, funding, or salary from an organization that holds or has applied for patents relating to the content of the manuscript?
- Do you have any other financial competing interests?
- Do you have any non-financial competing interests in relation to this paper?

If you can answer no to all of the above, write 'I declare that I have no competing interests' below. If your reply is yes to any, please give details below.

I declare that I have no competing interests'

I agree to the open peer review policy of the journal. I understand that my name will be included on my report to the authors and, if the manuscript is accepted for publication, my named report including any attachments I upload will be posted on the website along with the authors' responses. I agree for my report to be made available under an Open Access Creative Commons CC-BY license (http://creativecommons.org/licenses/by/4.0/). I understand that any comments which I do not wish to be included in my named report can be included as confidential comments to the editors, which will not be published.

I agree to the open peer review policy of the journal

To further support our reviewers, we have joined with Publons, where you can gain additional credit to further highlight your hard work (see: https://publons.com/journal/530/gigascience). On publication of this paper, your review will be automatically added to Publons, you can then choose whether or not to claim your Publons credit. I understand this statement.

Yes Choose an item.
