# Supplementary material for: Country specific hybridization of honey bees from lineage M
Source: Gigascience. 2026 Sep 15;15:giag093. doi: 10.1093/gigascience/giag093 (PMC13615543; doi:10.1093/gigascience/giag093)
Supplement: Supplementary file 5 [file giag093_reviewer_2_report_original_submission.pdf]

**Reviewer Report**

**Title: Country specific hybridization of honey bees from lineage M**

**Version: Original Submission** **Date:** 4/28/2026

**Reviewer name: Reviewer 2**

**Reviewer Comments to Author:**

This is an important paper, as it provides evidence to counter the argument that the M lineage of honey bees no longer exists in many European locations.
The data reported in the paper are not a randomised survey, but combine existing published material and new previously unpublished data, but with a patchy coverage, although Poland is comprehensively covered. The paper reports on some 29,000 images of wings which represent over 1,000 colonies from twelve countries. The images are now placed in a public repository, a valuable resource, as there are limited numbers of reference samples from many countries.
The results show that M Lineage is still prevalent in general bee populations in Ireland, Portugal, and Spain, but only exists in populations under some kind of protection in most other countries of its former range.
The paper explains that both molecular and morphometric techniques can be used to discriminate between populations, but notes that molecular techniques are not usually available to beekeepers, for whom morphometric techniques are more appropriate. There could perhaps be a comment added about divergence between the two techniques. What happens in beekeepers select populations on the basis of just a few wing morphometric characters? Maybe comment on this…
Sadly in many parts of its former area, such as Germany and the Czech Republic, A.m.m. may be effectively extinct, but the paper makes a plea for local beekeepers to work with locally adapted bees rather than trying to reintroduce A.m.m. from elsewhere, where the ecotypes are probably different from the extinct former populations. This is an important point.

**Methods**

Are the methods appropriate to the aims of the study, are they well described, and are necessary controls included? Choose an item.

**Conclusions**

Are the conclusions adequately supported by the data shown? Choose an item.

**Reporting Standards**

Does the manuscript adhere to the journal's guidelines on minimum standards of reporting? Choose an item.

Choose an item.

**Statistics**

Are you able to assess all statistics in the manuscript, including the appropriateness of statistical tests used? Choose an item.

**Quality of Written English**

Please indicate the quality of language in the manuscript: Choose an item.

**Declaration of Competing Interests**

Please complete a declaration of competing interests, considering the following questions:

- Have you in the past five years received reimbursements, fees, funding, or salary from an organisation that may in any way gain or lose financially from the publication of this manuscript, either now or in the future?
- Do you hold any stocks or shares in an organisation that may in any way gain or lose financially from the publication of this manuscript, either now or in the future?
- Do you hold or are you currently applying for any patents relating to the content of the manuscript?
- Have you received reimbursements, fees, funding, or salary from an organization that holds or has applied for patents relating to the content of the manuscript?
- Do you have any other financial competing interests?
- Do you have any non-financial competing interests in relation to this paper?

If you can answer no to all of the above, write 'I declare that I have no competing interests' below. If your reply is yes to any, please give details below.

I declare that I have no competing interests

I agree to the open peer review policy of the journal. I understand that my name will be included on my report to the authors and, if the manuscript is accepted for publication, my named report including any attachments I upload will be posted on the website along with the authors' responses. I agree for my report to be made available under an Open Access Creative Commons CC-BY license (http://creativecommons.org/licenses/by/4.0/). I understand that any comments which I do not wish to be included in my named report can be included as confidential comments to the editors, which will not be published.

I agree to the open peer review policy of the journal

To further support our reviewers, we have joined with Publons, where you can gain additional credit to further highlight your hard work (see: https://publons.com/journal/530/gigascience). On publication of this paper, your review will be automatically added to Publons, you can then choose whether or not to claim your Publons credit. I understand this statement.

Yes Choose an item.
